# Palladium-Catalyzed Esterification of Aryl Fluorosulfates with Aryl Formates

**DOI:** 10.3390/molecules29091991

**Published:** 2024-04-26

**Authors:** Xue Chen, Yuan Liang, Wen-Wen Wang, Chengping Miao, Xue-Qiang Chu, Weidong Rao, Hao Xu, Xiaocong Zhou, Zhi-Liang Shen

**Affiliations:** 1 Technical Institute of Fluorochemistry (TIF), School of Chemistry and Molecular Engineering, Nanjing Tech University, Nanjing 211816, China; cx202261105061@njtech.edu.cn (X.C.); 202361105065@njtech.edu.cn (Y.L.); 202361105069@njtech.edu.cn (W.-W.W.); xueqiangchu@njtech.edu.cn (X.-Q.C.); 2 College of Biological, Chemical Science and Engineering, Jiaxing University, 118 Jiahang Road, Jiaxing 314001, China; mcpschs@163.com; 3 Jiangsu Provincial Key Lab for the Chemistry and Utilization of Agro-Forest Biomass, College of Chemical Engineering, Nanjing Forestry University, Nanjing 210037, China; weidong@njfu.edu.cn

**Keywords:** palladium catalysis, esterification, ester, aryl fluorosulfates, aryl formates

## Abstract

An efficient palladium-catalyzed carbonylation of aryl fluorosulfates with aryl formates for the facile synthesis of esters was developed. The cross-coupling reactions proceeded effectively in the presence of a palladium catalyst, phosphine ligand, and triethylamine in DMF to produce the corresponding esters in moderate to good yields. Of note, functionalities or substituents, such as nitro, cyano, methoxycarbonyl, trifluoromethyl, methylsulfonyl, trifluoromethoxy, fluoro, chloro, bromo, methyl, methoxy, *N*,*N*-dimethyl, and [1,3]dioxolyl, were well-tolerated in the reactions, which could be kept for late-stage modification. The reactions employing readily available and relatively robust aryl fluorosulfates as coupling electrophiles could potentially serve as an attractive alternative to traditional cross-couplings with the use of aryl halides and pseudohalides as substrates.

## 1. Introduction

**Scheme 1:**
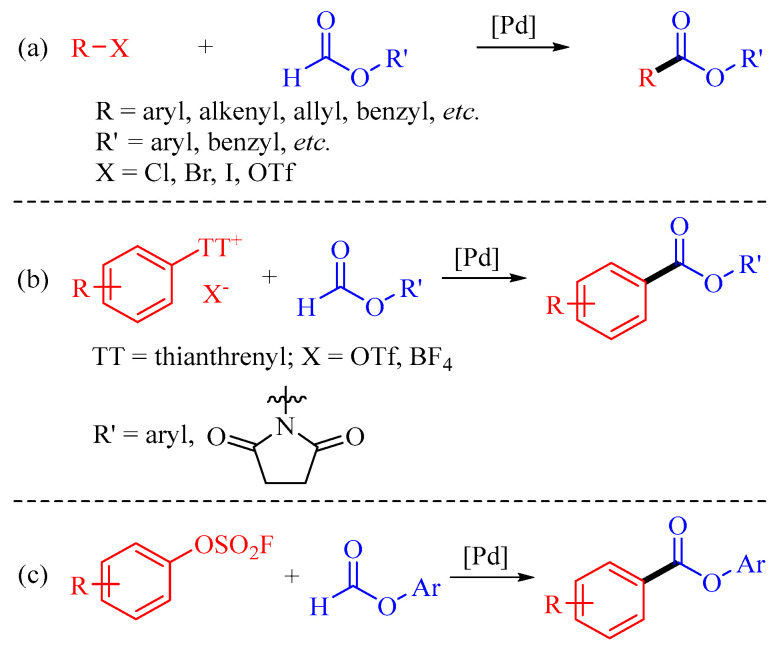
Carbonylation using formate as CO surrogate. (**a**) Coupling of organohalides with formates; (**b**) Coupling of aryl thianthrenium salt with formates; (**c**) Coupling of aryl fluorosulfates with formates (this work).

Esters not only widely exist in natural products, biologically/medicinally active molecules, and functional materials, but also serve as versatile and pivotal building blocks in organic synthesis [1,2,3,4]. In spite of many methods that have been well-developed for ester synthesis [1,2,3,4], the transition metal-catalyzed carbonylation has also been demonstrated to be a powerful platform for the construction of esters [5,6,7,8,9,10,11,12]. Among the various well-established carbonylative reactions, the use of formates as surrogates for toxic, flammable, and difficult-to-handle CO gas has also been proven to be an efficient and attractive approach for ester preparations [13,14,15,16,17,18,19,20,21,22,23,24,25,26,27,28,29,30,31,32,33,34,35,36,37,38,39,40,41,42,43,44,45,46,47]. For instance, the groups of Manabe, Wu, and others reported that palladium could efficiently catalyze the cross-coupling of organohalides with formates for entry to esters (Scheme 1a) [16,17,18,19,20,21,22,23,24,25,26,27,28]. In recent decades, developing alternative electrophiles as surrogates for conventional organohalides in carbonylation have also attracted much attention from the synthetic community. In this regard, in 2022, the Zhao group reported that aryl thianthrenium salt served as efficient electrophile to participate in palladium-catalyzed carbonylation with formates for the synthesis of esters (Scheme 1b) [47].

Apart from the above-mentioned coupling electrophiles (e.g., aryl halides and thianthrenium salt), aryl fluorosulfates as readily available and shelf-stable reagents have been demonstrated to be efficient electrophiles for undergoing a wide variety of organic transformations [48,49,50,51,52,53,54,55,56,57,58,59,60]. Generally, aryl fluorosulfates could be conveniently synthesized from the reactions of phenols with very cheap SO_2_F_2_ under weakly basic conditions (using Et_3_N as a base) with almost quantitative yields, and they showed higher reactivity than the corresponding aryl triflates, mesylates, and tosylates, serving as an attractive alternative to conventional aryl halides and pseudohalides [48,49]. Although several cases with the use of aryl fluorosulfates as substrates for palladium-catalyzed carbonylation have been developed, in most cases, dangerous CO gas was employed as a carbonyl source [61,62,63,64,65,66]. Thus, the development of an alternative method for the carbonylation of aryl fluorosulfate is still highly demanded. In the continuation of our efforts in developing efficient organic reactions with the use of alternative electrophiles [67,68,69,70,71,72,73,74,75,76,77,78,79], herein we report a palladium-catalyzed carbonylation of aryl fluorosulfate with aryl formate for the efficient access to ester (Scheme 1c).

## 2. Results

Our investigation began with the optimization of reaction conditions by using phenyl sulfurofluoridate (**1a**) and phenyl formate (**2a**) as substrates (Table 1). Initially, the model reaction was performed by employing various transition metal salts as a catalyst in the presence of 1,3-bis(diphenylphosphino)propane (dppp) and Et_3_N in DMF at 80 °C for 12 h. Among the different catalysts (including Fe(III), Co(II), Mn(III), Cr(III), Ni(II), and Pd(II)) screened (entries 1–6), Pd(acac)_2_ was found to be the catalyst of choice, leading to the corresponding ester **3a** in 73% NMR yield (entry 6). A subsequent survey of other palladium catalysts (entries 7–11) showed that the NMR yield of product **3a** could be slightly improved to 75% by using either Pd_2_(dba)_3_ or Pd(OAc)_2_ as a catalyst (entries 10–11). To further improve the reaction efficiency, a variety of ligands, bases, and solvents were investigated by utilizing Pd(OAc)_2_ as a catalyst (see Tables S1–S3 in the Supporting Materials for details). It was observed that the use of 4,5-bis(diphenylphosphino)-9,9-dimethylxanthene (XantPhos) to replace dppp as a ligand could deliver the desired product **3a** in 82% NMR yield (84% isolated yield, entry 12; also see entry 13 in Table S1). Evaluation of reaction temperatures showed that comparable yields could be obtained by performing the reactions at 60 °C or 100 °C (entries 13–14), while conducting the reaction at room temperature resulted in considerably eroded efficiency (entry 15). In addition, of the various bases and solvents examined, Et_3_N and DMF were found to be the optimal base and solvent for the present organic transformation (see Tables S2 and S3). Finally, control experiments (entries 16–18) indicated that palladium catalyst, base, and ligand were all essential for the efficient progress of the coupling reactions; without any of them, no expected reaction occurred.

**Figure 1:**
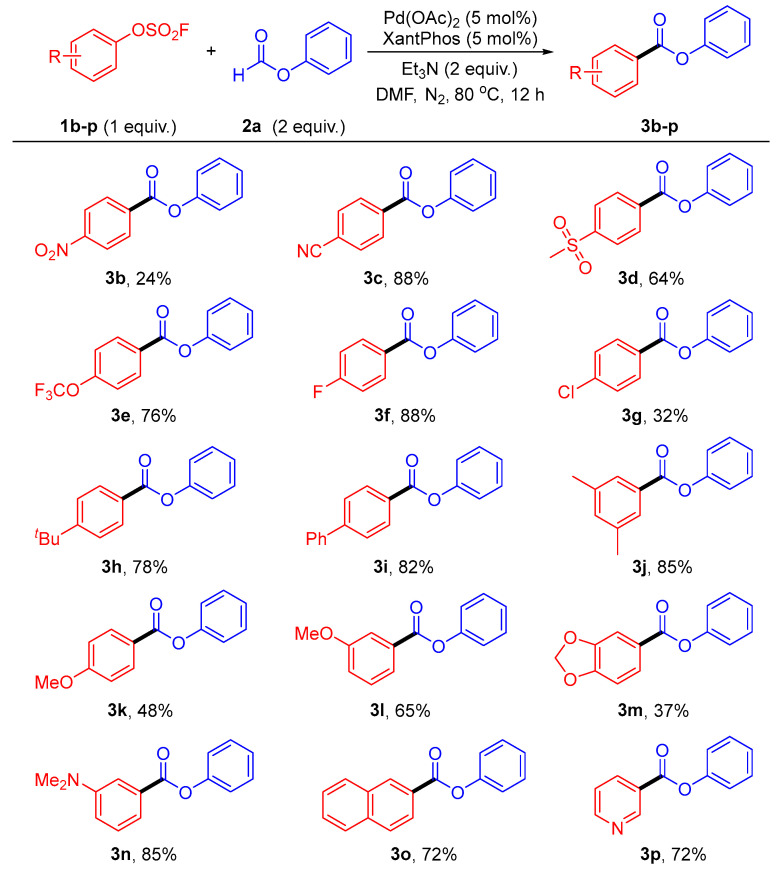
Substrate scope study by using various aryl fluorosulfates ^a,b^. ^a^ The reactions were performed at 80 °C for 12 h under nitrogen atmosphere by using **1b**–**p** (0.5 mmol), **2a** (1.0 mmol), Pd(OAc)_2_ (5 mol%), XantPhos (5 mol%), and Et_3_N (1.0 mmol) in anhydrous DMF (2 mL). ^b^ Isolated yield.

With the establishment of the optimal reaction conditions for the coupling reactions (Table 1, entry 12), we proceeded to examine substrate scope of the reaction by employing a range of structurally diverse aryl fluorosulfates. As illustrated in Figure 1, aryl fluorosulfates **1b**–**n** possessing either electron-withdrawing group or electron-donating substituent in the aryl ring efficiently underwent the cross-coupling with phenyl formate (**2a**) to produce the anticipated ester **3b**–**n** in 24–88% yields. In addition, naphthyl-substituted fluorosulfate **1o** worked equally well with **2a** under established conditions to give the product **3o** in 72% yield. Moreover, fluorosulfate **1p** derived from pyridine was proven to be a suitable candidate for the current esterification, providing the desired product **3p** in 72% yield. Notably, functional groups or substituents, including nitro, cyano, methylsulfonyl, trifluoromethoxy, fluoro, chloro, *t*-butyl, phenyl, methyl, methoxy, *N*,*N*-dimethyl, and [1,3]dioxolyl, were well-tolerated in the reaction, which could be retained for downstream derivatization. However, when sterically congested 2,6-dimethyl phenyl fluorosulfate was employed as a coupling partner, none of the desired cross-coupled product was obtained, presumably because of the inherent steric hindrance in the substrate.

**Figure 2:**
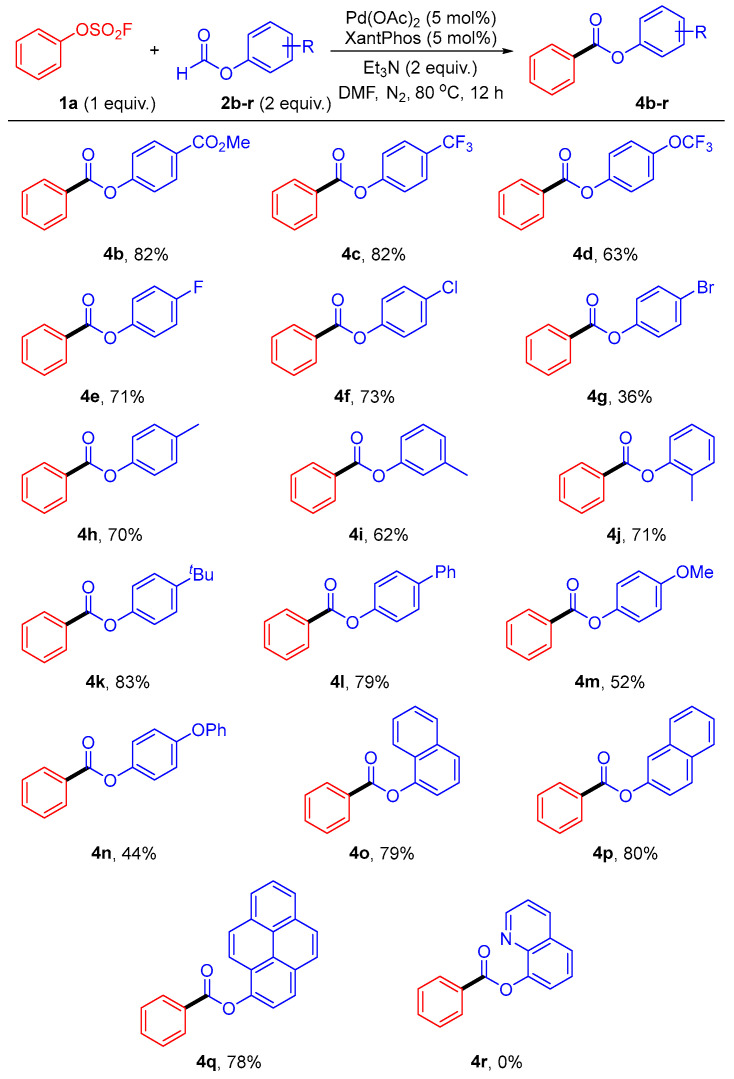
Substrate scope study by using various aryl formates ^a,b^. ^a^ The reactions were performed at 80 °C for 12 h under nitrogen atmosphere by using **1a** (0.5 mmol), **2b**–**r** (1.0 mmol), Pd(OAc)_2_ (5 mol%), XantPhos (5 mol%), and Et_3_N (1.0 mmol) in anhydrous DMF (2 mL). ^b^ Isolated yield.

Next, substrate scope with respect to aryl formates was studied (Figure 2). It was found that aryl formates **2b**–**n** derived from either an electron-poor or electron-rich phenyl ring were capable of efficiently taking part in the coupling reaction with phenyl sulfurofluoridate (**1a**), leading to the corresponding esters **4b**–**n** in modest to good yields. The starting material **1g** containing a relatively reactive C-Br bond could be employed as well, albeit generating the desired product **4g** in 36% yield. In addition, substrates **2h**–**j** bearing a methyl group at the *ortho*, *meta*, and *para* position of phenyl ring could react in a similar manner. Likewise, a plethora of functionalities, such as COOMe, CF_3_, OCF_3_, F, Cl, Br, Me, *^t^*Bu, Ph, OMe, and OPh, were well-amenable to the reaction. Moreover, naphthyl- and pyrenyl-substituted formates **2o**–**q** made good substrates as well, providing the anticipated esters **4o**–**q** in 78–80% yields. However, when heteroaryl formate **2r** derived from quinoline was subjected to cross-coupling with phenyl sulfurofluoridate (**1a**) under the optimized reaction conditions, none of expected product **4r** was obtained.

**Figure 3:**
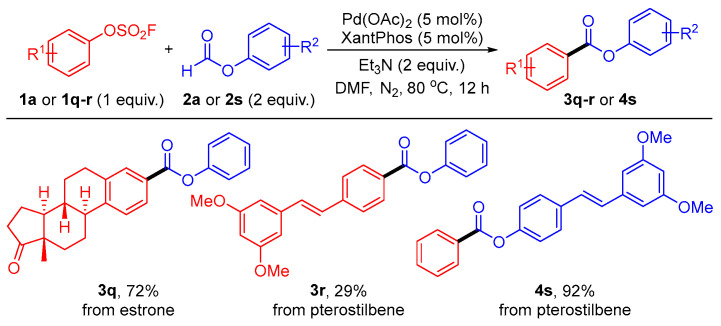
Late-stage derivatization of complex molecules ^a,b^. ^a^ The reactions were performed at 80 °C for 12 h under nitrogen atmosphere by using **1** (0.5 mmol), **2** (1.0 mmol), Pd(OAc)_2_ (5 mol%), XantPhos (5 mol%), and Et_3_N (1.0 mmol) in anhydrous DMF (2 mL). ^b^ Isolated yield.

The synthetic utility of the present coupling reaction was further demonstrated in the functionalization of substrates derived from naturally occurring molecules. As shown in Figure 3, the carbonylation could be applied to fluorosulfates **1q**–**r** and/or formate **2s** derived from estrone and pterostilbene under the developed conditions, producing the products **3q**–**r** and **4s** in 29–92% yields.

**Scheme 2:**
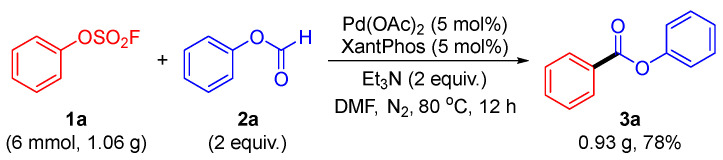
Gram-scale synthesis.

Finally, the current palladium-catalyzed carbonylation of aryl fluorosulfates with aryl formates was found to be amenable to gram-scale synthesis. As shown in Scheme 2, the 6 mmol-scale reaction using 1.06 g of aryl fluorosulfates **1a** as substrate proceeded equally well under well-established conditions to give the product **3a** in 78% yield.

**Figure 4:**
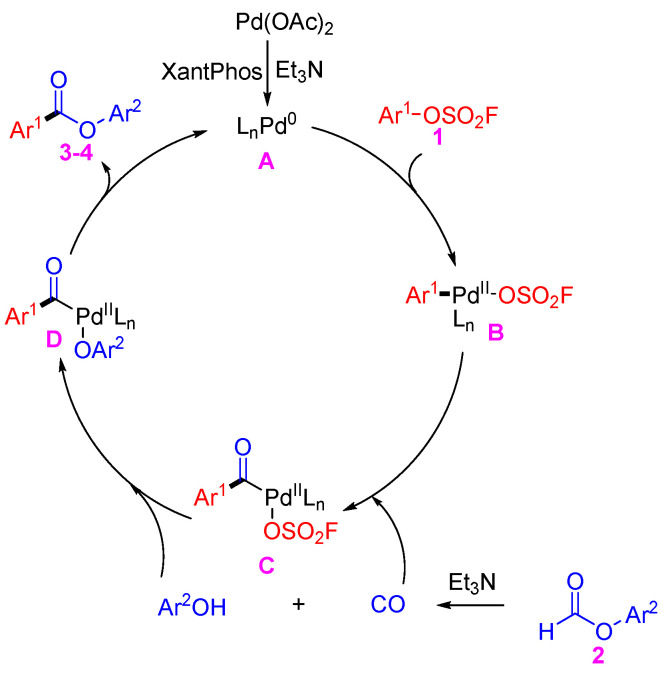
Proposed reaction mechanism.

Based on previous reports [16,17,18,19,20,21,22,23,24,25,26,27,28], a possible reaction mechanism was proposed (Figure 4). Initially, active zero-valent palladium species (L_n_Pd^0^) **A**, which is in situ generated in the reaction system, inserts into aryl fluorosulfate **1** to give an arylpalladium(II) intermediate **B**. On the other hand, carbon monoxide (CO) and phenol (Ar^2^OH) are in situ produced from aryl formate **2** under the action of Et_3_N. Next, the insertion of CO into organopalladium **B** gives an acylpalladium(II) species **C**, which undergoes exchange with phenol (Ar^2^OH) to obtain the corresponding phenoxy(acyl)palladium intermediate **D**. Finally, a reductive elimination of intermediate **D** occurs to furnish the desired product **3-4**, along with the re-production of palladium species **A**, which enters the next catalytic cycle.

## 3. Materials and Methods

### 3.1. General Information

Unless otherwise stated, all reagents were purchased from commercial suppliers and used without further purification. All the aryl fluorosulfates **1** and aryl formates **2** were prepared by following reported method [40,73,80]. Analytical thin layer chromatography (TLC) was performed using silica gel plate (0.2 mm thickness). Subsequent to elution, plates were visualized using UV radiation (254 nm). Flash chromatography was performed using Merck silica gel (200–300 mesh) for column chromatography with freshly distilled solvents. IR spectra were recorded on a FT-IR spectrophotometer using KBr optics. ^1^H, ^13^C, and ^19^F NMR spectra were recorded in CDCl_3_ on Bruker Avance or Jeol 400 MHz spectrometers. Tetramethylsilane (TMS) served as internal standard for ^1^H, ^13^C and ^19^F NMR analysis. High resolution mass spectra (HRMS) were obtained on a Waters Q-TOF Premier Spectrometer (ESI source).

### 3.2. Experimental

#### 3.2.1. General Procedure for the Synthesis of Aryl Fluorosulfates **1a**–**r**

A 500 mL single-neck round-bottom flask was sequentially charged with phenol (100 mmol, 1.0 equiv.), dichloromethane (250 mL, 0.4 M), and triethylamine (42 mL, 300 mmol, 3.0 equiv.), and it was then sealed with a rubber septum. The atmosphere above the solution was removed by gentle vacuum, and SO_2_F_2_ gas was subsequently introduced into the flask by a needle from a balloon filled with SO_2_F_2_ gas. The reaction mixture was vigorously stirred at room temperature for 12 h. The solvent was evaporated, and the residual was purified by silica gel column chromatography using petroleum ether and EtOAc as eluent to obtain the pure product of aryl fluorosulfate. Spectral data of these compounds are in accordance with those previously documented [73,80]. 

#### 3.2.2. General Procedure for the Synthesis of Aryl Formates **2a**–**s**

Formic acid (2.3 mL, 60 mmol, 6.0 equiv.) was added to acetic anhydride (3.8 mL, 40 mmol, 4.0 equiv.) at room temperature. The mixture was stirred at 60 °C (oil bath) for 4 h and cooled to room temperature. The resulting solution was transferred to a 100 mL single-neck round-bottom flask containing phenol (10 mmol, 1.0 equiv.) and NaOAc (0.82 g, 10 mmol, 1.0 equiv.). The mixture was stirred at room temperature for 4 h. Then, CH_2_Cl_2_ and water was added to the mixture and the organic layer was extracted with CH_2_Cl_2_ (60 mL × 3), washed with saturated NaHCO_3_ solution and brine, and dried over anhydrous Na_2_SO_4_. The extracts were concentrated under reduced pressure to obtain the crude product, which was further purified through silica gel column chromatography using petroleum ether and EtOAc as eluent to obtain the pure product of aryl formate. Aryl formates **2a**–**r** are known compounds, and their characterization data are in accord with the reported ones [40,47]. Aryl formate **2s** is a new compound, and its characterization data are shown below.

#### 3.2.3. General Procedure for the Cross-Coupling of Aryl Fluorosulfates with Aryl Formates

An oven-dried sealed tube containing a stirring bar was charged with Pd(OAc)_2_ (5.6 mg, 0.025 mmol, 5 mol%) and XantPhos (14.5 mg, 0.025 mmol, 5 mol%). The sealed tube was then evacuated and backfilled with nitrogen gas three times. The anhydrous DMF (2 mL), aryl fluorosulfate **1** (0.5 mmol, 1 equiv.), aryl formate **2** (1.0 mmol, 2 equiv.), and Et_3_N (101.2 mg, 1.0 mmol, 2 equiv.) were then added under nitrogen atmosphere. The reaction mixture was stirred at 80 °C (oil bath) for 12 h before quenching with saturated NH_4_Cl solution (10 mL) and extracting with EtOAc (20 mL × 3). The organic layers were combined, washed with saturated brine, and dried over Na_2_SO_4_. The extracts were concentrated under reduced pressure to obtain the crude product, which was further purified through silica gel column chromatography (using EtOAc/petroleum ether as eluents) to yield the products **3**-**4**. 

#### 3.2.4. Gram-Scale Synthesis of Aryl Fluorosulfate **1a** with Aryl Formate **2a**

An oven-dried sealed tube containing a stirring bar was charged with Pd(OAc)_2_ (67.4 mg, 0.3 mmol, 5 mol%) and XantPhos (173.6 mg, 0.3 mmol, 5 mol%). The sealed tube was then evacuated and backfilled with nitrogen gas three times. The anhydrous DMF (10 mL), aryl fluorosulfate **1a** (1057.0 mg, 6 mmol, 1 equiv.), aryl formate **2a** (1465.4 mg, 12 mmol, 2 equiv.), and Et_3_N (1214.3 mg, 12 mmol, 2 equiv.) were then added under nitrogen atmosphere. The reaction mixture was stirred at 80 °C (oil bath) for 12 h before quenching with saturated NH_4_Cl solution (20 mL) and extracting with EtOAc (60 mL × 3). The organic layers were combined, washed with brine, and dried over Na_2_SO_4_. The extracts were concentrated under reduced pressure to obtain the crude product, which was further purified through silica gel column chromatography (using EtOAc/petroleum ether as eluents) to yield the product **3a** in 78% yield (931.5 mg).

*(E)-4-(3,5-Dimethoxystyryl)phenyl formate* (**2r**). Yield = 98%, 2.79 g (10 mmol scale). White solid. ^1^H NMR (400 MHz, CDCl_3_): *δ* 8.32 (s, 1H), 7.58–7.49 (m, 2H), 7.17–6.96 (m, 4H), 6.67 (d, *J* = 2.3 Hz, 2H), 6.42 (t, *J* = 2.3 Hz, 1H), 3.83 (s, 6H) ppm. ^13^C NMR (100 MHz, CDCl_3_): *δ* 160.9, 159.2, 149.1, 138.9, 135.4, 129.2, 127.8, 127.6, 121.3, 104.5, 100.0, 55.3 ppm. IR (KBr, neat): *ν* = 3084, 1502, 1267, 1224, 1174, 977, 853, 670 cm^−1^. HRMS (ESI, *m*/*z*): calcd for C_17_H_17_O_4_^+^ [M+H]^+^ 285.1121, found: 285.1122.

*Phenyl benzoate* (**3a**). This product was purified by silica gel column chromatography using petroleum ether/ethyl acetate as eluent (petroleum ether/EtOAc = 100:1). Yield = 84%, 83.3 mg. White solid. ^1^H NMR (400 MHz, CDCl_3_): *δ* 8.29–8.23 (m, 2H), 7.70–7.64 (m, 1H), 7.55 (dd, *J* = 8.4, 7.0 Hz, 2H), 7.51–7.44 (m, 2H), 7.35–7.24 (m, 3H) ppm. ^13^C NMR (100 MHz, CDCl_3_): *δ* 165.1, 150.8, 133.5, 130.1, 129.4 (2C), 128.5, 125.8, 121.6 ppm. IR (KBr, neat): *ν* = 3326, 1730, 1486, 1262, 1198, 1062, 751, 704 cm^−1^. HRMS (ESI, *m*/*z*): calcd for C_13_H_11_O_2_^+^ [M+H]^+^ 199.0754, found: 199.0750. Spectral data of the product are in accordance with those previously documented [47].

*Phenyl 4-nitrobenzoate* (**3b**). This product was purified by silica gel column chromatography using petroleum ether/ethyl acetate as eluent (petroleum ether/EtOAc = 50:1). Yield = 24%, 28.8 mg. White solid. ^1^H NMR (400 MHz, CDCl_3_): *δ* 8.43–8.31 (m, 4H), 7.53–7.42 (m, 2H), 7.36–7.29 (m, 1H), 7.27–7.19 (m, 2H) ppm. ^13^C NMR (100 MHz, CDCl_3_): *δ* 163.3, 150.9, 150.5, 134.9, 131.3, 129.7, 126.4, 123.7, 121.4 ppm. IR (KBr, neat): *ν* = 3338, 2855, 1742, 1528, 1269, 1184, 1079, 711 cm^−1^. HRMS (ESI, *m*/*z*): calcd for C_13_H_10_NO_4_^+^ [M+H]^+^ 244.0604, found: 244.0604. Spectral data of the product are in accordance with those previously documented [81].

*Phenyl 4-cyanobenzoate* (**3c**). This product was purified by silica gel column chromatography using petroleum ether/ethyl acetate as eluent (petroleum ether/EtOAc = 50:1). Yield = 88%, 98.2 mg. White solid. ^1^H NMR (400 MHz, CDCl_3_): *δ* 8.12–8.06 (m, 2H), 7.62–7.56 (m, 2H), 7.28–7.20 (m, 2H), 7.12–7.06 (m, 1H), 7.03–6.98 (m, 2H) ppm. ^13^C NMR (100 MHz, CDCl_3_): *δ* 163.5, 150.4, 133.2, 132.3, 130.5, 129.6, 126.2, 121.3, 117.8, 116.8 ppm. IR (KBr, neat): *ν* = 3320, 2233, 1740, 1483, 1268, 864, 763, 688 cm^−1^. HRMS (ESI, *m*/*z*): calcd for C_14_H_10_NO_2_^+^ [M+H]^+^ 224.0706, found: 224.0702. Spectral data of the product are in accordance with those previously documented [81].

*Phenyl 4-(methylsulfonyl)benzoate* (**3d**). This product was purified by silica gel column chromatography using petroleum ether/ethyl acetate as eluent (petroleum ether/EtOAc = 10:1). Yield = 64%, 88.8 mg. White solid. ^1^H NMR (400 MHz, CDCl_3_): *δ* 8.43–8.38 (m, 2H), 8.13–8.08 (m, 2H), 7.49–7.43 (m, 2H), 7.34–7.28 (m, 1H), 7.25–7.20 (m, 2H), 3.11 (s, 3H) ppm. ^13^C NMR (100 MHz, CDCl_3_): *δ* 163.6, 150.5, 144.8, 134.3, 131.1, 129.6, 127.7, 126.3, 121.4, 44.3 ppm. IR (KBr, neat): *ν* = 3054, 1732, 1280, 1216, 1079, 829, 742, 689 cm^−1^. HRMS (ESI, *m*/*z*): calcd for C_14_H_13_O_4_S^+^ [M+H]^+^ 277.0529, found: 277.0528. Spectral data of the product are in accordance with those previously documented [82].

*Phenyl 4-(trifluoromethoxy)benzoate* (**3e**). This product was purified by silica gel column chromatography using petroleum ether/ethyl acetate as eluent (petroleum ether/EtOAc = 100:1). Yield = 76%, 106.7 mg. White solid. ^1^H NMR (400 MHz, CDCl_3_): *δ* 8.30–8.23 (m, 2H), 7.49–7.41 (m, 2H), 7.38–7.27 (m, 3H), 7.25–7.18 (m, 2H) ppm. ^13^C NMR (100 MHz, CDCl_3_): *δ* 164.0, 153.0, 150.6, 132.2, 129.6, 127.8, 126.1, 121.6, 120.4, 120.2 (q, *J* = 257.9 Hz) ppm. ^19^F NMR (376 MHz, CDCl_3_): *δ* -57.45 (s, 3F) ppm. IR (KBr, neat): *ν* = 3326, 1735, 1505, 1264, 1073, 918, 765, 691 cm^−1^. HRMS (ESI, *m*/*z*): calcd for C_14_H_10_F_3_O_3_^+^ [M+H]^+^ 283.0577, found: 283.0575. Spectral data of the product are in accordance with those previously documented [83].

*Phenyl 4-fluorobenzoate* (**3f**). This product was purified by silica gel column chromatography using petroleum ether/ethyl acetate as eluent (petroleum ether/EtOAc = 100:1). Yield = 88%, 95.5 mg. White solid. ^1^H NMR (400 MHz, CDCl_3_): *δ* 8.27–8.19 (m, 2H), 7.47–7.41 (m, 2H), 7.32–7.26 (m, 1H), 7.25–7.14 (m, 4H) ppm. ^13^C NMR (100 MHz, CDCl_3_): *δ* 166.1 (d, *J* = 255.1 Hz), 164.2, 150.8, 132.8 (d, *J* = 9.4 Hz), 129.5, 126.0, 125.8 (d, *J* = 2.8 Hz), 121.6, 115.8 (d, *J* = 22.1 Hz) ppm. ^19^F NMR (376 MHz, CDCl_3_): *δ* -104.30 (s, 1F) ppm. IR (KBr, neat): *ν* = 3331, 2921, 1733, 1596, 1506, 1278, 745, 687 cm^−1^. HRMS (ESI, *m*/*z*): calcd for C_13_H_10_FO_2_^+^ [M+H]^+^ 217.0659, found: 217.0658. Spectral data of the product are in accordance with those previously documented [81].

*Phenyl 4-chlorobenzoate* (**3g**). This product was purified by silica gel column chromatography using petroleum ether/ethyl acetate as eluent (petroleum ether/EtOAc = 100:1). Yield = 32%, 37.6 mg. White solid. ^1^H NMR (400 MHz, CDCl_3_): *δ* 8.21–8.14 (m, 2H), 7.54–7.43 (m, 4H), 7.35–7.29 (m, 1H), 7.26–7.21 (m, 2H) ppm. ^13^C NMR (100 MHz, CDCl_3_): *δ* 164.3, 150.6, 140.0, 131.5, 129.5, 128.9, 127.9, 126.0, 121.6 ppm. IR (KBr, neat): *ν* = 3325, 2696, 1731, 1591, 1280, 1075, 852, 754 cm^−1^. HRMS (ESI, *m*/*z*): calcd for C_13_H_10_ClO_2_^+^ [M+H]^+^ 233.0364, found: 233.0364. Spectral data of the product are in accordance with those previously documented [47].

*Phenyl 4-(tert-butyl)benzoate* (**3h**). This product was purified by silica gel column chromatography using petroleum ether/ethyl acetate as eluent (petroleum ether/EtOAc = 100:1). Yield = 78%, 99.2 mg. White solid. ^1^H NMR (400 MHz, CDCl_3_): *δ* 8.19–8.15 (m, 2H), 7.58–7.53 (m, 2H), 7.48–7.42 (m, 2H), 7.31–7.26 (m, 1H), 7.25–7.21 (m, 2H), 1.39 (s, 9H) ppm. ^13^C NMR (100 MHz, CDCl_3_): *δ* 165.2, 157.3, 151.0, 130.0, 129.4, 126.7, 125.7, 125.5, 121.7, 35.2, 31.1 ppm. IR (KBr, neat): *ν* = 3332, 3055, 2958, 1732, 1267, 1185, 1073, 750 cm^−1^. HRMS (ESI, *m*/*z*): calcd for C_17_H_19_O_2_^+^ [M+H]^+^ 255.1380, found: 255.1379. Spectral data of the product are in accordance with those previously documented [47].

*Phenyl [1,1’-biphenyl]-4-carboxylate* (**3i**). This product was purified by silica gel column chromatography using petroleum ether/ethyl acetate as eluent (petroleum ether/EtOAc = 100:1). Yield = 82%, 112.2 mg. White solid. ^1^H NMR (400 MHz, CDCl_3_): *δ* 8.31–8.25 (m, 2H), 7.77–7.72 (m, 2H), 7.69–7.64 (m, 2H), 7.53–7.40 (m, 5H), 7.32–7.27 (m, 1H), 7.26–7.22 (m, 2H) ppm. ^13^C NMR (100 MHz, CDCl_3_): *δ* 165.1, 151.0, 146.3, 139.8, 130.7, 129.5, 129.0, 128.3, 128.2, 127.3, 127.2, 125.9, 121.7 ppm. IR (KBr, neat): *ν* = 3326, 2852, 1730, 1605, 1265, 1082, 857, 696 cm^−1^. HRMS (ESI, *m*/*z*): calcd for C_19_H_15_O_2_^+^ [M+H]^+^ 275.1067, found: 275.1069. Spectral data of the product are in accordance with those previously documented [47].

*Phenyl 3,5-dimethylbenzoate* (**3j**). This product was purified by silica gel column chromatography using petroleum ether/ethyl acetate as eluent (petroleum ether/EtOAc = 100:1). Yield = 85%, 96.0 mg. White solid. ^1^H NMR (400 MHz, CDCl_3_): *δ* 7.85 (s, 2H), 7.48–7.42 (m, 2H), 7.32–7.26 (m, 2H), 7.25–7.20 (m, 2H), 2.42 (s, 6H) ppm. ^13^C NMR (100 MHz, CDCl_3_): *δ* 165.5, 151.0, 138.2, 135.2, 129.4, 129.3, 127.8, 125.8, 121.7, 21.1 ppm. IR (KBr, neat): *ν* = 3327, 2918, 1735, 1310, 1100, 896, 731, 505 cm^−1^. HRMS (ESI, *m*/*z*): calcd for C_15_H_15_O_2_^+^ [M+H]^+^ 227.1067, found: 227.1066. Spectral data of the product are in accordance with those previously documented [47].

*Phenyl 4-methoxybenzoate* (**3k**). This product was purified by silica gel column chromatography using petroleum ether/ethyl acetate as eluent (petroleum ether/EtOAc = 100:1). Yield = 48%, 54.8 mg. White solid. ^1^H NMR (400 MHz, CDCl_3_): *δ* 8.26–8.20 (m, 2H), 7.53–7.46 (m, 2H), 7.36–7.25 (m, 3H), 7.09–7.02 (m, 2H), 3.95 (s, 3H) ppm. ^13^C NMR (100 MHz, CDCl_3_): *δ* 164.9, 163.8, 151.0, 132.2, 129.4, 125.7, 121.79, 121.76, 113.8, 55.5 ppm. IR (KBr, neat): *ν* = 3328, 2930, 1726, 1607, 1510, 1164, 763, 564 cm^−1^. HRMS (ESI, *m*/*z*): calcd for C_14_H_13_O_3_^+^ [M+H]^+^ 229.0859, found: 229.0859. Spectral data of the product are in accordance with those previously documented [47].

*Phenyl 3-methoxybenzoate* (**3l**). This product was purified by silica gel column chromatography using petroleum ether/ethyl acetate as eluent (petroleum ether/EtOAc = 100:1). Yield = 65%, 73.9 mg. White solid. ^1^H NMR (400 MHz, CDCl_3_): *δ* 7.83 (dt, *J* = 7.7, 1.2 Hz, 1H), 7.74–7.71 (m, 1H), 7.48–7.40 (m, 3H), 7.32–7.27 (m, 1H), 7.25–7.17 (m, 3H), 3.89 (s, 3H) ppm. ^13^C NMR (100 MHz, CDCl_3_): *δ* 165.0, 159.6, 150.9, 130.8, 129.6, 129.5, 125.9, 122.5, 121.7, 120.1, 114.4, 55.5 ppm. IR (KBr, neat): *ν* = 3316, 3055, 2918, 1725, 1598, 1484, 1276, 741 cm^−1^. HRMS (ESI, *m*/*z*): calcd for C_14_H_13_O_3_^+^ [M+H]^+^ 229.0859, found: 229.0857. Spectral data of the product are in accordance with those previously documented [82].

*Phenyl benzo[d][1,3]dioxole-5-carboxylate* (**3m**). This product was purified by silica gel column chromatography using petroleum ether/ethyl acetate as eluent (petroleum ether/EtOAc = 100:1). Yield = 37%, 45.0 mg. White solid. ^1^H NMR (400 MHz, CDCl_3_): *δ* 7.83 (dd, *J* = 8.2, 1.7 Hz, 1H), 7.62 (d, *J* = 1.7 Hz, 1H), 7.46–7.39 (m, 2H), 7.30–7.24 (m, 1H), 7.23–7.17 (m, 2H), 6.91 (d, *J* = 8.2 Hz, 1H), 6.08 (s, 2H) ppm. ^13^C NMR (100 MHz, CDCl_3_): *δ* 164.5, 152.2, 150.9, 147.9, 129.4, 126.2, 125.8, 123.4, 121.7, 109.9, 108.1, 101.9 ppm. IR (KBr, neat): *ν* = 3325, 2898, 1723, 1608, 1489, 1445, 1193, 750 cm^−1^. HRMS (ESI, *m*/*z*): calcd for C_14_H_11_O_4_^+^ [M+H]^+^ 243.0652, found: 243.0650. Spectral data of the product are in accordance with those previously documented [47].

*Phenyl 3-(dimethylamino)benzoate* (**3n**). This product was purified by silica gel column chromatography using petroleum ether/ethyl acetate as eluent (petroleum ether/EtOAc = 10:1). Yield = 85%, 103.1 mg. White solid. ^1^H NMR (400 MHz, CDCl_3_): *δ* 7.55–7.49 (m, 2H), 7.42–7.36 (m, 2H), 7.35–7.29 (m, 1H), 7.25–7.16 (m, 3H), 6.96–6.91 (m, 1H), 2.98 (s, 6H) ppm. ^13^C NMR (100 MHz, CDCl_3_): *δ* 165.8, 151.1, 150.5, 130.1, 129.4, 129.1, 125.7, 121.8, 118.0, 117.3, 113.5, 40.5 ppm. IR (KBr, neat): *ν* = 3330, 2809, 1733, 1600, 1189, 1089, 990, 691 cm^−1^. HRMS (ESI, *m*/*z*): calcd for C_15_H_16_NO_2_^+^ [M+H]^+^ 242.1176, found: 242.1175. Spectral data of the product are in accordance with those previously documented [82].

*Phenyl 2-naphthoate* (**3o**). This product was purified by silica gel column chromatography using petroleum ether/ethyl acetate as eluent (petroleum ether/EtOAc = 100:1). Yield = 72%, 88.9 mg. White solid. ^1^H NMR (400 MHz, CDCl_3_): *δ* 8.83–8.78 (m, 1H), 8.26–8.18 (m, 1H), 8.06–7.90 (m, 3H), 7.69–7.55 (m, 2H), 7.50–7.44 (m, 2H), 7.34–7.27 (m, 3H) ppm. ^13^C NMR (100 MHz, CDCl_3_): *δ* 165.3, 151.0, 135.8, 132.4, 131.9, 129.5, 129.4, 128.6, 128.4, 127.8, 126.8, 126.7, 125.9, 125.4, 121.7 ppm. IR (KBr, neat): *ν* = 3054, 1732, 1591, 1486, 1280, 829, 742, 689 cm^−1^. HRMS (ESI, *m*/*z*): calcd for C_17_H_13_O_2_^+^ [M+H]^+^ 249.0910, found: 249.0913. Spectral data of the product are in accordance with those previously documented [81].

*Phenyl nicotinate* (**3p**). This product was purified by silica gel column chromatography using petroleum ether/ethyl acetate as eluent (petroleum ether/EtOAc = 10:1). Yield = 72%, 71.3 mg. White solid. ^1^H NMR (400 MHz, CDCl_3_): *δ* 9.22 (dd, *J* = 2.2, 0.9 Hz, 1H), 8.67 (dd, *J* = 4.9, 1.8 Hz, 1H), 8.27 (dt, *J* = 8.0, 2.0 Hz, 1H), 7.31–7.24 (m, 3H), 7.14–7.09 (m, 1H), 7.07–7.02 (m, 2H) ppm. ^13^C NMR (100 MHz, CDCl_3_): *δ* 163.8, 154.0, 151.3, 150.4, 137.5, 129.6, 126.2, 125.5, 123.4, 121.5 ppm. IR (KBr, neat): *ν* = 3042, 1740, 1589, 1490, 1428, 1277, 732, 707 cm^−1^. HRMS (ESI, *m*/*z*): calcd for C_12_H_10_NO_2_^+^ [M+H]^+^ 200.0706, found: 200.0704. Spectral data of the product are in accordance with those previously documented [83].

*Phenyl (8R,9S,13S,14S)-13-methyl-17-oxo-7,8,9,11,12,13,14,15,16,17-decahydro-6H-cyclopenta[a]phenanthrene-3-carboxylate* (**3q**). This product was purified by silica gel column chromatography using petroleum ether/ethyl acetate as eluent (petroleum ether/EtOAc = 50:1). Yield = 72%, 135.2 mg. White solid. ^1^H NMR (400 MHz, CDCl_3_): *δ* 8.00–7.93 (m, 2H), 7.46–7.39 (m, 3H), 7.30–7.24 (m, 1H), 7.23–7.17 (m, 2H), 3.05–2.96 (m, 2H), 2.58–2.43 (m, 2H), 2.37 (td, *J* = 10.9, 4.1 Hz, 1H), 2.23–1.97 (m, 4H), 1.72–1.45 (m, 6H), 0.93 (s, 3H) ppm. ^13^C NMR (100 MHz, CDCl_3_): *δ* 220.5, 165.2, 150.9, 145.9, 136.9, 130.7, 129.4, 127.4, 126.8, 125.7, 125.6, 121.6, 50.4, 47.8, 44.6, 37.7, 35.7, 31.4, 29.1, 26.1, 25.5, 21.5, 13.7 ppm. IR (KBr, neat): *ν* = 3446, 3044, 2939, 1717, 1568, 1493, 739, 505 cm^−1^. HRMS (ESI, *m*/*z*): calcd for C_25_H_27_O_3_^+^ [M+H]^+^ 375.1955, found: 375.1957.

*Phenyl (E)-4-(3,5-dimethoxystyryl)benzoate* (**3r**). This product was purified by silica gel column chromatography using petroleum ether/ethyl acetate as eluent (petroleum ether/EtOAc = 100:1). Yield = 29%, 51.8 mg. White solid. ^1^H NMR (400 MHz, CDCl_3_): *δ* 8.22–8.16 (m, 2H), 7.66–7.60 (m, 2H), 7.49–7.40 (m, 3H), 7.25–7.14 (m, 4H), 6.71 (d, *J* = 2.3 Hz, 2H), 6.45 (t, *J* = 2.2 Hz, 1H), 3.85 (s, 6H) ppm. ^13^C NMR (100 MHz, CDCl_3_): *δ* 165.0, 161.0, 150.9, 142.3, 138.6, 131.6, 130.6, 129.5, 128.3, 127.9, 126.5, 125.9, 121.7, 104.8, 100.6, 55.4 ppm. IR (KBr, neat): *ν* = 3529, 3314, 2918, 1735, 1592, 1310, 1194, 689 cm^−1^. HRMS (ESI, *m*/*z*): calcd for C_23_H_21_O_4_^+^ [M+H]^+^ 361.1434, found: 361.1435.

*Methyl 4-(benzoyloxy)benzoate* (**4b**). This product was purified by silica gel column chromatography using petroleum ether/ethyl acetate as eluent (petroleum ether/EtOAc = 50:1). Yield = 82%, 105.7 mg. Colorless oil. ^1^H NMR (400 MHz, CDCl_3_): *δ* 8.22–8.18 (m, 2H), 8.14–8.10 (m, 2H), 7.67–7.61 (m, 1H), 7.54–7.48 (m, 2H), 7.33–7.28 (m, 2H), 3.92 (s, 3H) ppm. ^13^C NMR (100 MHz, CDCl_3_): *δ* 166.3, 164.5, 154.5, 133.8, 131.1, 130.2, 129.0, 128.6, 127.7, 121.7, 52.1 ppm. IR (KBr, neat): *ν* = 3320, 2956, 1723, 1602, 1440, 1266, 761, 698 cm^−1^. HRMS (ESI, *m*/*z*): calcd for C_15_H_13_O_4_ ^+^ [M+H]^+^ 257.0808, found: 257.0805. Spectral data of the product are in accordance with those previously documented [81].

*4-(Trifluoromethyl)phenyl benzoate* (**4c**). This product was purified by silica gel column chromatography using petroleum ether/ethyl acetate as eluent (petroleum ether/EtOAc = 100:1). Yield = 82%, 108.5 mg. Colorless oil. ^1^H NMR (400 MHz, CDCl_3_): *δ* 8.26–8.19 (m, 2H), 7.75–7.64 (m, 3H), 7.57–7.51 (m, 2H), 7.40–7.34 (m, 2H) ppm. ^13^C NMR (100 MHz, CDCl_3_): *δ* 164.6, 153.4 (q, *J* = 1.9 Hz), 133.9, 130.2, 128.9, 128.7, 128.4 (q, *J* = 32.5 Hz), 126.8 (q, *J* = 3.9 Hz), 125.1 (q, *J* = 260.0 Hz), 122.2 ppm. ^19^F NMR (376 MHz, CDCl_3_): *δ* -62.05 (s, 3F) ppm. IR (KBr, neat): *ν* = 3325, 2851, 1732, 1453, 1118, 882, 704, 593 cm^−1^. HRMS (ESI, *m*/*z*): calcd for C_14_H_10_F_3_O_2_^+^ [M+H]^+^ 267.0627, found: 267.0626. Spectral data of the product are in accordance with those previously documented [81].

*4-(Trifluoromethoxy)phenyl benzoate* (**4d**). This product was purified by silica gel column chromatography using petroleum ether/ethyl acetate as eluent (petroleum ether/EtOAc = 100:1). Yield = 63%, 88.2 mg. Colorless oil. ^1^H NMR (400 MHz, CDCl_3_): *δ* 8.22–8.17 (m, 2H), 7.67–7.62 (m, 1H), 7.55–7.49 (m, 2H), 7.31–7.23 (m, 4H) ppm. ^13^C NMR (100 MHz, CDCl_3_): *δ* 164.9, 149.2, 146.6, 133.8, 130.2, 129.1, 128.6, 123.0, 122.2, 120.4 (q, *J* = 255.9 Hz) ppm. ^19^F NMR (376 MHz, CDCl_3_): *δ* -57.99 (s, 3F) ppm. IR (KBr, neat): *ν* = 3066, 2921, 1910, 1731, 1508, 1154, 705, 535 cm^−1^. HRMS (ESI, *m*/*z*): calcd for C_14_H_10_F_3_O_3_^+^ [M+H]^+^ 283.0577, found: 283.0574. Spectral data of the product are in accordance with those previously documented [84].

*4-Fluorophenyl benzoate* (**4e**). This product was purified by silica gel column chromatography using petroleum ether/ethyl acetate as eluent (petroleum ether/EtOAc = 100:1). Yield = 71%, 77.0 mg. White solid. ^1^H NMR (400 MHz, CDCl_3_): *δ* 8.23–8.17 (m, 2H), 7.69–7.62 (m, 1H), 7.56–7.50 (m, 2H), 7.22–7.16 (m, 2H), 7.16–7.08 (m, 2H) ppm. ^13^C NMR (100 MHz, CDCl_3_): *δ* 165.2, 160.3 (d, *J* = 244.2 Hz), 146.7 (d, *J* = 2.8 Hz), 133.7, 130.1, 129.2, 128.6, 123.1 (d, *J* = 8.5 Hz), 116.1 (d, *J* = 23.5 Hz) ppm. ^19^F NMR (376 MHz, CDCl_3_): *δ* -116.80 (s, 1F) ppm. IR (KBr, neat): *ν* = 3326, 2359, 1732, 1504, 1188, 1063, 706, 526 cm^−1^. HRMS (ESI, *m*/*z*): calcd for C_13_H_10_FO_2_^+^ [M+H]^+^ 217.0659, found: 217.0659. Spectral data of the product are in accordance with those previously documented [83].

*4-Chlorophenyl benzoate* (**4f**). This product was purified by silica gel column chromatography using petroleum ether/ethyl acetate as eluent (petroleum ether/EtOAc = 100:1). Yield = 73%, 84.9 mg. White solid. ^1^H NMR (400 MHz, CDCl_3_): *δ* 8.23–8.18 (m, 2H), 7.72–7.61 (m, 1H), 7.58–7.48 (m, 2H), 7.44–7.36 (m, 2H), 7.23–7.14 (m, 2H) ppm. ^13^C NMR (100 MHz, CDCl_3_): *δ* 164.9, 149.3, 133.8, 131.2, 130.1, 129.5, 129.1, 128.6, 123.1 ppm. IR (KBr, neat): *ν* = 3062, 1732, 1488, 1266, 1060, 876, 705, 513 cm^−1^. HRMS (ESI, *m*/*z*): calcd for C_13_H_10_ClO_2_^+^ [M+H]^+^ 233.0364, found: 233.0361. Spectral data of the product are in accordance with those previously documented [81].

*4-Bromophenyl benzoate* (**4g**). This product was purified by silica gel column chromatography using petroleum ether/ethyl acetate as eluent (petroleum ether/EtOAc = 100:1). Yield = 36%, 50.3 mg. White solid. ^1^H NMR (400 MHz, CDCl_3_): *δ* 8.22–8.16 (m, 2H), 7.69–7.62 (m, 1H), 7.58–7.49 (m, 4H), 7.15–7.09 (m, 2H) ppm. ^13^C NMR (100 MHz, CDCl_3_): *δ* 164.9, 149.9, 133.8, 132.5, 130.2, 129.1, 128.6, 123.5, 119.0 ppm. IR (KBr, neat): *ν* = 3333, 2849, 1732, 1486, 1280, 1059, 805, 510 cm^−1^. HRMS (ESI, *m*/*z*): calcd for C_13_H_10_BrO_2_^+^ [M+H]^+^ 276.9859, found: 276.9862. Spectral data of the product are in accordance with those previously documented [83].

*p-Tolyl benzoate* (**4h**). This product was purified by silica gel column chromatography using petroleum ether/ethyl acetate as eluent (petroleum ether/EtOAc = 100:1). Yield = 70%, 73.8 mg. White solid. ^1^H NMR (400 MHz, CDCl_3_): *δ* 8.24–8.20 (m, 2H), 7.68–7.61 (m, 1H), 7.56–7.49 (m, 2H), 7.26–7.21 (m, 2H), 7.14–7.08 (m, 2H), 2.39 (s, 3H) ppm. ^13^C NMR (100 MHz, CDCl_3_): *δ* 165.4, 148.6, 135.5, 133.5, 130.1, 130.0, 129.6, 128.5, 121.3, 20.9 ppm. IR (KBr, neat): *ν* = 3328, 2920, 1727, 1597, 1508, 1270, 706, 511 cm^−1^. HRMS (ESI, *m*/*z*): calcd for C_14_H_13_O_2_^+^ [M+H]^+^ 213.0910, found: 213.0907. Spectral data of the product are in accordance with those previously documented [81].

*m-Tolyl benzoate* (**4i**). This product was purified by silica gel column chromatography using petroleum ether/ethyl acetate as eluent (petroleum ether/EtOAc = 100:1). Yield = 62%, 65.6 mg. White solid. ^1^H NMR (400 MHz, CDCl_3_): *δ* 8.25–8.22 (m, 2H), 7.68–7.62 (m, 1H), 7.56–7.50 (m, 2H), 7.34 (t, *J* = 7.7 Hz, 1H), 7.13–7.02 (m, 3H), 2.41 (s, 3H) ppm. ^13^C NMR (100 MHz, CDCl_3_): *δ* 165.3, 150.8, 139.6, 133.5, 130.1, 129.6, 129.2, 128.5, 126.7, 122.3, 118.6, 21.3 ppm. IR (KBr, neat): *ν* = 3325, 2921, 1735, 1487, 1237, 1145, 1064, 707 cm^−1^. HRMS (ESI, *m*/*z*): calcd for C_14_H_13_O_2_^+^ [M+H]^+^ 213.0910, found: 213.0910. Spectral data of the product are in accordance with those previously documented [85].

*o-Tolyl benzoate* (**4j**). This product was purified by silica gel column chromatography using petroleum ether/ethyl acetate as eluent (petroleum ether/EtOAc = 100:1). Yield = 71%, 74.9 mg. White solid. ^1^H NMR (400 MHz, CDCl_3_): *δ* 8.28 (dt, *J* = 8.5, 1.6 Hz, 2H), 7.70–7.65 (m, 1H), 7.58–7.53 (m, 2H), 7.34–7.27 (m, 2H), 7.25–7.16 (m, 2H), 2.28 (s, 3H) ppm. ^13^C NMR (100 MHz, CDCl_3_): *δ* 164.8, 149.5, 133.5, 131.1, 130.2, 130.1, 129.4, 128.6, 126.9, 126.0, 121.9, 16.2 ppm. IR (KBr, neat): *ν* = 2927, 1735, 1491, 1266, 1111, 1024, 748, 706 cm^−1^. HRMS (ESI, *m*/*z*): calcd for C_14_H_13_O_2_^+^ [M+H]^+^ 213.0910, found: 213.0910. Spectral data of the product are in accordance with those previously documented [85].

*4-(tert-Butyl)phenyl benzoate* (**4k**). This product was purified by silica gel column chromatography using petroleum ether/ethyl acetate as eluent (petroleum ether/EtOAc = 100:1). Yield = 83%, 105.3 mg. White solid. ^1^H NMR (400 MHz, CDCl_3_): *δ* 8.26–8.21 (m, 2H), 7.68–7.62 (m, 1H), 7.53 (ddt, *J* = 8.1, 6.6, 1.0 Hz, 2H), 7.49–7.44 (m, 2H), 7.19–7.14 (m, 2H), 1.36 (s, 9H) ppm. ^13^C NMR (100 MHz, CDCl_3_): *δ* 165.3, 148.6, 148.5, 133.5, 130.1, 129.6, 128.5, 126.4, 120.9, 34.5, 31.4 ppm. IR (KBr, neat): *ν* = 3057, 2963, 1732, 1598, 1508, 1262, 707, 552 cm^−1^. HRMS (ESI, *m*/*z*): calcd for C_17_H_19_O_2_^+^ [M+H]^+^ 255.1380, found: 255.1382. Spectral data of the product are in accordance with those previously documented [85].

*[1,1’-Biphenyl]-4-yl benzoate* (**4l**). This product was purified by silica gel column chromatography using petroleum ether/ethyl acetate as eluent (petroleum ether/EtOAc = 100:1). Yield = 79%, 108.7 mg. White solid. ^1^H NMR (400 MHz, CDCl_3_): *δ* 8.28–8.24 (m, 2H), 7.70–7.65 (m, 3H), 7.65–7.61 (m, 2H), 7.58–7.52 (m, 2H), 7.51–7.45 (m, 2H), 7.41–7.36 (m, 1H), 7.35–7.30 (m, 2H) ppm. ^13^C NMR (100 MHz, CDCl_3_): *δ* 165.2, 150.3, 140.4, 139.0, 133.6, 130.2, 129.5, 128.8, 128.6, 128.2 127.3, 127.1, 122.0 ppm. IR (KBr, neat): *ν* = 3325, 2923, 1731, 1598, 1486, 1280, 758, 703 cm^−1^. HRMS (ESI, *m*/*z*): calcd for C_19_H_15_O_2_^+^ [M+H]^+^ 275.1067, found: 275.1068. Spectral data of the product are in accordance with those previously documented [81].

*4-Methoxyphenyl benzoate* (**4m**). This product was purified by silica gel column chromatography using petroleum ether/ethyl acetate as eluent (petroleum ether/EtOAc = 100:1). Yield = 52%, 59.0 mg. White solid. ^1^H NMR (400 MHz, CDCl_3_): *δ* 8.23–8.19 (m, 2H), 7.66–7.61 (m, 1H), 7.54–7.48 (m, 2H), 7.17–7.11 (m, 2H), 6.97–6.92 (m, 2H), 3.83 (s, 3H) ppm. ^13^C NMR (100 MHz, CDCl_3_): *δ* 165.5, 157.3, 144.4, 133.5, 130.1, 129.6, 128.5, 122.4, 114.5, 55.6 ppm. IR (KBr, neat): *ν* = 3323, 2837, 1724, 1506, 1247, 1192, 1025, 814 cm^−1^. HRMS (ESI, *m*/*z*): calcd for C_14_H_13_O_3_^+^ [M+H]^+^ 229.0859, found: 229.0862. Spectral data of the product are in accordance with those previously documented [81].

*4-Phenoxyphenyl benzoate* (**4n**). This product was purified by silica gel column chromatography using petroleum ether/ethyl acetate as eluent (petroleum ether/EtOAc = 100:1). Yield = 44%, 63.7 mg. White solid. ^1^H NMR (400 MHz, CDCl_3_): *δ* 8.26–8.19 (m, 2H), 7.70–7.61 (m, 1H), 7.57–7.48 (m, 2H), 7.41–7.32 (m, 2H), 7.23–7.17 (m, 2H), 7.16–7.11 (m, 1H), 7.10–7.02 (m, 4H) ppm. ^13^C NMR (100 MHz, CDCl_3_): *δ* 165.3, 157.2, 154.8, 146.3, 133.6, 130.1, 129.8, 129.4, 128.6, 123.4, 122.8, 119.6, 118.8 ppm. IR (KBr, neat): *ν* = 3332, 2734, 1732, 1588, 1500, 1188, 1065, 708 cm^−1^. HRMS (ESI, *m*/*z*): calcd for C_19_H_15_O_3_^+^ [M+H]^+^ 291.1016, found: 291.1016. Spectral data of the product are in accordance with those previously documented [83].

*Naphthalen-1-yl benzoate* (**4o**). This product was purified by silica gel column chromatography using petroleum ether/ethyl acetate as eluent (petroleum ether/EtOAc = 100:1). Yield = 79%, 98.1 mg. White solid. ^1^H NMR (400 MHz, CDCl_3_): *δ* 8.43–8.35 (m, 2H), 8.01–7.98 (m, 1H), 7.96–7.92 (m, 1H), 7.83 (d, *J* = 8.3 Hz, 1H), 7.75–7.68 (m, 1H), 7.63–7.52 (m, 5H), 7.43 (dd, *J* = 7.5, 1.0 Hz, 1H) ppm. ^13^C NMR (100 MHz, CDCl_3_): *δ* 165.2, 146.8, 134.6, 133.7, 130.3, 129.3, 128.7, 128.0, 126.9, 126.5, 126.4, 126.0, 125.4, 121.2, 118.2 ppm. IR (KBr, neat): *ν* = 3060, 1740, 1598, 1451, 1254, 1021, 871, 704 cm^−1^. HRMS (ESI, *m*/*z*): calcd for C_17_H_13_O_2_^+^ [M+H]^+^ 249.0910, found: 249.0909. Spectral data of the product are in accordance with those previously documented [81].

*Naphthalen-2-yl benzoate* (**4p**). This product was purified by silica gel column chromatography using petroleum ether/ethyl acetate as eluent (petroleum ether/EtOAc = 100:1). Yield = 80%, 99.2 mg. White solid. ^1^H NMR (400 MHz, CDCl_3_): *δ* 8.36–8.28 (m, 2H), 7.98–7.84 (m, 3H), 7.78–7.72 (m, 1H), 7.72–7.64 (m, 1H), 7.61–7.48 (m, 4H), 7.46–7.37 (m, 1H) ppm. ^13^C NMR (100 MHz, CDCl_3_): *δ* 165.3, 148.5, 133.8, 133.6, 131.5, 130.2, 129.5, 129.4, 128.6, 127.8, 127.6, 126.6, 125.7, 121.2, 118.7 ppm. IR (KBr, neat): *ν* = 3328, 2921, 1729, 1451, 1238, 1062, 708, 478 cm^−1^. HRMS (ESI, *m*/*z*): calcd for C_17_H_13_O_2_^+^ [M+H]^+^ 249.0910, found: 249.0911. Spectral data of the product are in accordance with those previously documented [81].

*Pyren-1-yl benzoate* (**4q**). This product was purified by silica gel column chromatography using petroleum ether/ethyl acetate as eluent (petroleum ether/EtOAc = 100:1). Yield = 78%, 125.0 mg. White solid. ^1^H NMR (400 MHz, CDCl_3_): *δ* 8.48–8.41 (m, 2H), 8.26–8.14 (m, 4H), 8.11–8.01 (m, 4H), 7.96–7.90 (m, 1H), 7.78–7.71 (m, 1H), 7.67–7.59 (m, 2H) ppm. ^13^C NMR (100 MHz, CDCl_3_): *δ* 165.6, 144.5, 133.8, 131.1, 130.9, 130.4, 129.4, 129.3, 128.8, 128.2, 127.1, 126.3, 125.6, 125.5, 125.2, 125.0, 124.5, 123.3, 120.3, 119.8 ppm. IR (KBr, neat): *ν* = 3446, 3047, 1735, 1597, 1452, 1229, 1065, 705 cm^−1^. HRMS (ESI, *m*/*z*): calcd for C_23_H_15_O_2_^+^ [M+H]^+^ 323.1067, found: 323.1065. Spectral data of the product are in accordance with those previously documented [86].

*(E)-4-(3,5-Dimethoxystyryl)phenyl benzoate* (**4s**). This product was purified by silica gel column chromatography using petroleum ether/ethyl acetate as eluent (petroleum ether/EtOAc = 100:1). Yield = 92%, 166.5 mg. White solid. ^1^H NMR (400 MHz, CDCl_3_): *δ* 8.27–8.22 (m, 2H), 7.69–7.63 (m, 1H), 7.60–7.50 (m, 4H), 7.27–7.23 (m, 2H), 7.16–7.00 (m, 2H), 6.72 (d, *J* = 2.2 Hz, 2H), 6.45 (t, *J* = 2.3 Hz, 1H), 3.84 (s, 6H) ppm. ^13^C NMR (100 MHz, CDCl_3_): *δ* 165.0, 160.8, 150.2, 139.0, 134.8, 133.5, 130.0, 129.3, 128.7, 128.5, 128.0, 127.4, 121.8, 104.4, 99.9, 55.2 ppm. IR (KBr, neat): *ν* = 3327, 2832, 1727, 1591, 1455, 1152, 1065, 707 cm^−1^. HRMS (ESI, *m*/*z*): calcd for C_23_H_21_O_4_^+^ [M+H]^+^ 361.1434, found: 361.1437.

## 4. Conclusions

In summary, an efficient protocol for the easy entry to esters through the palladium-catalyzed esterification of aryl fluorosulfates with aryl formates was realized. The carbonylative reactions efficiently occurred with the aid of palladium catalyst, phosphine ligand, and triethylamine in DMF to provide the desired esters in modest to high yields with good tolerance to a range of functional groups and substituents embedded in the aryl ring. In addition, the reaction could not only be scaled up with good efficiency, but also be applied to the utilization of substrates derived from naturally occurring estrone and pterostilbene.

## Figures and Tables

**Table 1:** Optimization of reaction conditions by using various catalysts ^a^.

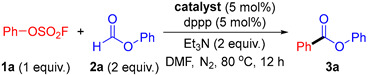		
**Entry**	**Catalyst**	**Yield (%) ^b^**
1	Fe(acac)_3_	0
2	Co(acac)_2_	0
3	Mn(acac)_3_	0
4	Cr(acac)_3_	0
5	Ni(acac)_2_	0
6	Pd(acac)_2_	73
7	PdCl_2_	39
8	Pd(PPh_3_)_2_Cl_2_	28
9	Pd(PPh_3_)_4_	67
10	Pd_2_(dba)_3_	75
11	Pd(OAc)_2_	75
12	Pd(OAc)_2_	82 ^c^ (84) ^d^
13	Pd(OAc)_2_	80 ^e^
14	Pd(OAc)_2_	83 ^f^
15	Pd(OAc)_2_	21 ^g^
16	-	0 ^c^
17	Pd(OAc)_2_	0 ^c,h^
18	Pd(OAc)_2_	0 ^i^

^a^ The reactions were performed at 80 °C for 12 h under nitrogen atmosphere by using **1a** (0.5 mmol), **2a** (1.0 mmol), catalyst (5 mol%), dppp (5 mol%), and Et_3_N (1.0 mmol) in anhydrous DMF (2 mL). ^b^ Yields were determined by NMR analysis of crude reaction mixture after work-up by using 1,4-dimethoxybenzene as an internal standard. ^c^ Using XantPhos instead of dppp as ligand. ^d^ Isolated yield. ^e^ At 60 °C. ^f^ At 100 °C. ^g^ At room temperature. ^h^ Without Et_3_N. ^i^ Without dppp.

## Data Availability

Data are contained within the article and Supplementary Materials.

## Supplementary Materials

The following supporting information can be downloaded at: https://www.mdpi.com/article/10.3390/molecules29091991/s1, optimization of reaction conditions, and copies of the ^1^H NMR and ^13^C NMR spectra of investigated compounds are available online.
