# Supplementary material for: Palladium-Catalyzed Esterification of Aryl Fluorosulfates with Aryl Formates
Source: Molecules. 2024 Apr 26;29(9):1991. doi: 10.3390/molecules29091991 (PMC11085239; doi:10.3390/molecules29091991)

Supporting Information

Palladium-catalyzed esterification of aryl fluorosulfates with aryl formates

Xue Chen,[a] Yuan Liang,[a] Wen-Wen Wang,[a] Chengping Miao,[b] Xue-Qiang Chu,[a] Weidong Rao,[c] Hao Xu,*[,a] Xiaocong Zhou,*[,a,b] and Zhi-Liang Shen*[,a]

[a] Technical Institute of Fluorochemistry (TIF), Institute of Advanced Synthesis, School of Chemistry and Molecular Engineering, Nanjing Tech University, Nanjing 211816, China. E-mail: xuhao@njtech.edu.cn, ias_zlshen@njtech.edu.cn

[b] College of Biological, Chemical Science and Engineering, Jiaxing University, 118 Jiahang Road, Jiaxing 314001, China. E-mail: xczhou@zjxu.edu.cn

[c] Jiangsu Provincial Key Lab for the Chemistry and Utilization of Agro-Forest Biomass, College of Chemical Engineering, Nanjing Forestry University, Nanjing 210037, China.

Table of Contents

Optimization of reaction conditions……………...………………………...……...…..........page S6

Figure S1. ^{1}H NMR spectrum of **2s** (400 MHz, $CDCl_3$) ………....…………..……..……......page S8

Figure S2. ^{13}C NMR spectrum of **2s** (100 MHz, $CDCl_3$) ………....……………..……..….........page S8

Figure S3. ^{1}H NMR spectrum of **3a** (400 MHz, $CDCl_3$) ………....……………..………….......page S9

Figure S4. ^{13}C NMR spectrum of **3a** (100 MHz, $CDCl_3$) ………....……………..……...….......page S9

Figure S5. ^{1}H NMR spectrum of **3b** (400 MHz, $CDCl_3$) ………....……………..……..…........page S10

Figure S6. ^{13}C NMR spectrum of **3b** (100 MHz, $CDCl_3$) ………....……………..……..…........page S10

Figure S7. ^{1}H NMR spectrum of **3c** (400 MHz, $CDCl_3$) ……….....….……….……..….......page S11

Figure S8. ^{13}C NMR spectrum of **3c** (100 MHz, $CDCl_3$) ……….....……………….……..….......page S11

Figure S9. ^{1}H NMR spectrum of **3d** (400 MHz, $CDCl_3$) ……….....……………….……..….......page S12

Figure S10. ^{13}C NMR spectrum of **3d** (100 MHz, $CDCl_3$) ……....……………….……..….......page S12

Figure S11. ^{1}H NMR spectrum of **3e** (400 MHz, $CDCl_3$) ……….....……………….……..….......page S13

Figure S12. ^{13}C NMR spectrum of **3e** (100 MHz, $CDCl_3$) ……….....………….…....….......page S13

Figure S13. ^{19}F NMR spectrum of **3e** (376 MHz, $CDCl_3$) ……….....………....……..….......page S14

Figure S14. ^{1}H NMR spectrum of **3f** (400 MHz, $CDCl_3$) ……….....……………….……..….......page S14

Figure S15. ^{13}C NMR spectrum of **3f** (100 MHz, $CDCl_3$) ……….....………..……...….......page S15

Figure S16. ^{19}F NMR spectrum of **3f** (376 MHz, $CDCl_3$) ……….....……..….……...….......page S15

Figure S17. ^{1}H NMR spectrum of **3g** (400 MHz, $CDCl_3$) ……….....……………….……..….......page S16

Figure S18. ^{13}C NMR spectrum of **3g** (100 MHz, $CDCl_3$) ……....……………….……..….......page S16

Figure S19. ^{1}H NMR spectrum of **3h** (400 MHz, $CDCl_3$) ……….....…...…….……..….......page S17

Figure S20. ^{13}C NMR spectrum of **3h** (100 MHz, $CDCl_3$) ……....……………….……..….......page S17

Figure S21. ^{1}H NMR spectrum of **3i** (400 MHz, $CDCl_3$) ……….....……………….……..….......page S18

Figure S22. ^{13}C NMR spectrum of **3i** (100 MHz, $CDCl_3$) ……….....………....……..….......page S18

Figure S23. ^{1}H NMR spectrum of **3j** (400 MHz, $CDCl_3$) ……….....……………….……..….......page S19

Figure S24. ^{13}C NMR spectrum of **3j** (100 MHz, $CDCl_3$) ……….....………....……...….......page S19

Figure S25. ^{1}H NMR spectrum of **3k** (400 MHz, $CDCl_3$) ……….....………....….....….......page S20

Figure S26. ^{13}C NMR spectrum of **3k** (100 MHz, $CDCl_3$) ……….....……….…….……..….......page S20

Figure S27. ^{1}H NMR spectrum of **3l** (400 MHz, $CDCl_3$) ……….....……………….……..….......page S21

Figure S28. ^{13}C NMR spectrum of **3l** (100 MHz, $CDCl_3$) ……….....……………….……..….......page S21

Figure S29. ^{1}H NMR spectrum of **3m** (400 MHz, $CDCl_3$) ……….………….……..….......page S22

Figure S30. ^{13}C NMR spectrum of **3m** (100 MHz, $CDCl_3$) ……….………….……..….......page S22

Figure S31. ^{1}H NMR spectrum of **3n** (400 MHz, $CDCl_3$) ……….………….……..….......page S23

Figure S32. ^{13}C NMR spectrum of **3n** (100 MHz, $CDCl_3$) ……….………….……..….......page S23

Figure S33. ^{1}H NMR spectrum of **3o** (400 MHz, $CDCl_3$) ……….………….……..….......page S24

Figure S34. ^{13}C NMR spectrum of **3o** (100 MHz, $CDCl_3$) ……….………….……..….......page S24

Figure S35. ^{1}H NMR spectrum of **3p** (400 MHz, $CDCl_3$) ……….………….……..….......page S25

Figure S36. ^{13}C NMR spectrum of **3p** (100 MHz, $CDCl_3$) ……….………….……..….......page S25

Figure S37. ^{1}H NMR spectrum of **3q** (400 MHz, $CDCl_3$) ……….………….……..….......page S26

Figure S38. ^{13}C NMR spectrum of **3q** (100 MHz, $CDCl_3$) ……….………….……..….......page S26

Figure S39. ^{1}H NMR spectrum of **3r** (400 MHz, $CDCl_3$) ……….………….……..….......page S27

Figure S40. ^{13}C NMR spectrum of **3r** (100 MHz, $CDCl_3$) ……….………….……..….......page S27

Figure S41. ^{1}H NMR spectrum of **4b** (400 MHz, $CDCl_3$) ……….………….……..….......page S28

Figure S42. ^{13}C NMR spectrum of **4b** (100 MHz, $CDCl_3$) ……….………….……..….......page S28

Figure S43. ^{1}H NMR spectrum of **4c** (400 MHz, $CDCl_3$) ……….………….……..….......page S29

Figure S44. ^{13}C NMR spectrum of **4c** (100 MHz, $CDCl_3$) ……….………….……..….......page S29

Figure S45. ^{19}F NMR spectrum of **4c** (376 MHz, $CDCl_3$) ……….………….……..….......page S30

Figure S46. ^{1}H NMR spectrum of **4d** (400 MHz, $CDCl_3$) ……….………….……..….......page S30

Figure S47. ^{13}C NMR spectrum of **4d** (100 MHz, $CDCl_3$) ……….………….……..….......page S31

Figure S48. ^{19}F NMR spectrum of **4d** (376 MHz, $CDCl_3$) ……….………….……..….......page S31

Figure S49. ^{1}H NMR spectrum of **4e** (400 MHz, $CDCl_3$) ……….………….……..….......page S32

Figure S50. ^{13}C NMR spectrum of **4e** (100 MHz, $CDCl_3$) ……….………….……..….......page S32

Figure S51. ^{19}F NMR spectrum of **4e** (376 MHz, $CDCl_3$) ……….....……….....……...….......page S33

Figure S52. ^{1}H NMR spectrum of **4f** (400 MHz, $CDCl_3$) ……….....…………….……...….......page S33

Figure S53. ^{13}C NMR spectrum of **4f** (100 MHz, $CDCl_3$) ……….....……………..……..….......page S34

Figure S54. ^{1}H NMR spectrum of **4g** (400 MHz, $CDCl_3$) ……….....………...………...….......page S34

Figure S55. ^{13}C NMR spectrum of **4g** (100 MHz, $CDCl_3$) ……….....………….....…...….......page S35

Figure S56. ^{1}H NMR spectrum of **4h** (400 MHz, $CDCl_3$) ……….....………………...….......page S35

Figure S57. ^{13}C NMR spectrum of **4h** (100 MHz, $CDCl_3$) ……….....……….....……...….......page S36

Figure S58. ^{1}H NMR spectrum of **4i** (400 MHz, $CDCl_3$) ……….....…………..……...….......page S36

Figure S59. ^{13}C NMR spectrum of **4i** (100 MHz, $CDCl_3$) ……….....…….....……...….......page S37

Figure S60. ^{1}H NMR spectrum of **4j** (400 MHz, $CDCl_3$) ……….....……………….....….......page S37

Figure S61. ^{13}C NMR spectrum of **4j** (100 MHz, $CDCl_3$) ……….....………………...….......page S38

Figure S62. ^{1}H NMR spectrum of **4k** (400 MHz, $CDCl_3$) ……….......…………..……...….......page S38

Figure S63. ^{13}C NMR spectrum of **4k** (100 MHz, $CDCl_3$) ……….....……………...….......page S39

Figure S64. ^{1}H NMR spectrum of **4l** (400 MHz, $CDCl_3$) ……….....………………...….......page S39

Figure S65. ^{13}C NMR spectrum of **4l** (100 MHz, $CDCl_3$) ……….......…………...….......page S40

Figure S66. ^{1}H NMR spectrum of **4m** (400 MHz, $CDCl_3$) ……….....……..……...….......page S40

Figure S67. ^{13}C NMR spectrum of **4m** (100 MHz, $CDCl_3$) ……….....…………...….......page S41

Figure S68. ^{1}H NMR spectrum of **4n** (400 MHz, $CDCl_3$) ……….....……………...….......page S41

Figure S69. ^{13}C NMR spectrum of **4n** (100 MHz, $CDCl_3$) ……….....…….....……...….......page S42

Figure S70. ^{1}H NMR spectrum of **4o** (400 MHz, $CDCl_3$) ……….....…..……..……...….......page S42

Figure S71. ^{13}C NMR spectrum of **4o** (100 MHz, $CDCl_3$) ……….....……..……...….......page S43

Figure S72. ^{1}H NMR spectrum of **4p** (400 MHz, $CDCl_3$) ……….....………….……..….......page S43

Figure S73. ^{13}C NMR spectrum of **4p** (100 MHz, $CDCl_3$) ………...…….….……..….......page S44

Figure S74. ^{1}H NMR spectrum of **4q** (400 MHz, $CDCl_3$) …………..…….….……..….......page S44

Figure S75. ^{13}C NMR spectrum of **4q** (100 MHz, $CDCl_3$) ………....…….….……..….......page S45

Figure S76. ^{1}H NMR spectrum of **4s** (400 MHz, $CDCl_3$) ………....….…….….…..……......page S45

Figure S77. ^{13}C NMR spectrum of **4s** (100 MHz, $CDCl_3$) ………....…………...…..….......page S46

Optimization of reaction conditions

Table S1. Optimization of reaction conditions by using different ligands[a]

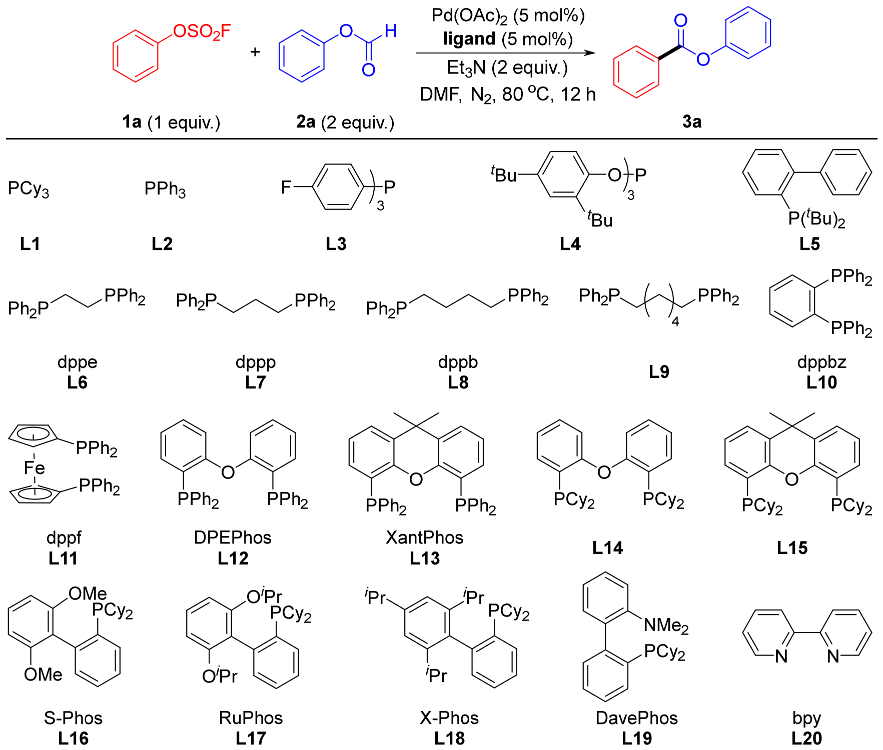

Entry	Ligand	Yield (%)[b]	Entry	Ligand	Yield (%)[b]
1	**L1**	0	11	**L11**	trace
2	**L2**	0	12	**L12**	8
3	**L3**	0	**13**	**L13**	**82 (84)**[c]
4	**L4**	0	14	**L14**	trace
5	**L5**	0	15	**L15**	trace
6	**L6**	trace	16	**L16**	trace
7	**L7**	75	17	**L17**	0
8	**L8**	76	18	**L18**	0
9	**L9**	78	19	**L19**	trace
10	**L10**	13	20	**L20**	0

[a] The reactions were performed at 80 °C for 12 h under nitrogen atmosphere by using **1a** (0.5 mmol), **2a** (1.0 mmol), $Pd(OAc)_2$ (5 mol%), ligand (5 mol%), and Et_3N (1.0 mmol) in anhydrous DMF (2 mL). [b] Yields were determined by NMR analysis of crude reaction mixture after work-up by using 1,4-dimethoxybenzene as an internal standard. [c] Isolated yield.

Table S2. Optimization of reaction conditions by using different bases[a]

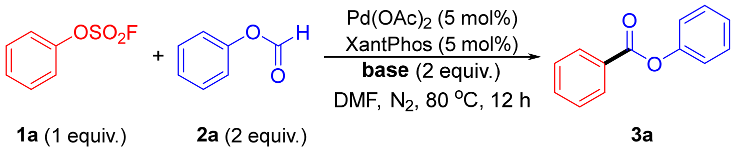

Entry	Base	Yield (%)[b]	Entry	Base	Yield (%)[b]
**1**	**Et_3N**	**82 (84)**[c]	6	^{t}BuONa	15
2	DIPEA	79	7	Cs_2CO_3	trace
3	DMAP	77	8	K_2CO_3	28
4	DBU	0	9	$NaHCO_3$	54
5	Pyridine	0	10	NaOH	trace

[a] The reactions were performed at 80 °C for 12 h under nitrogen atmosphere by using **1a** (0.5 mmol), **2a** (1.0 mmol), $Pd(OAc)_2$ (5 mol%), XantPhos (5 mol%), and base (1.0 mmol) in anhydrous DMF (2 mL). [b] Yields were determined by NMR analysis of crude reaction mixture after work-up by using 1,4-dimethoxybenzene as an internal standard. [c] Isolated yield.

Table S3. Optimization of reaction conditions by using different solvents[a]

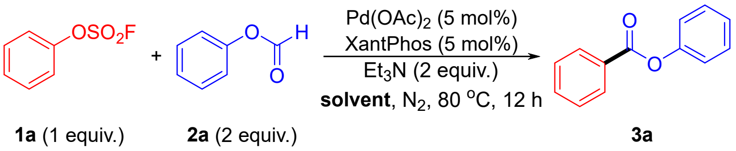

Entry	Solvent	Yield (%)[b]	Entry	Solvent	Yield (%)[b]
**1**	**DMF**	**82 (84)**[c]	6	MeCN	10
2	DMA	15	7	MeOH	0
3	NMP	18	8	1,4-dioxane	30
4	DMPU	13	9	THF	80
5	DMSO	49	10	toluene	27

[a] The reactions were performed at 80 °C for 12 h under nitrogen atmosphere by using **1a** (0.5 mmol), **2a** (1.0 mmol), $Pd(OAc)_2$ (5 mol%), XantPhos (5 mol%), and Et_3N (1.0 mmol) in solvent (2 mL). [b] Yields were determined by NMR analysis of crude reaction mixture after work-up by using 1,4-dimethoxybenzene as an internal standard. [c] Isolated yield.

Figure S1. ^{1}H NMR spectrum of **2s** (400 MHz, $CDCl_3$)

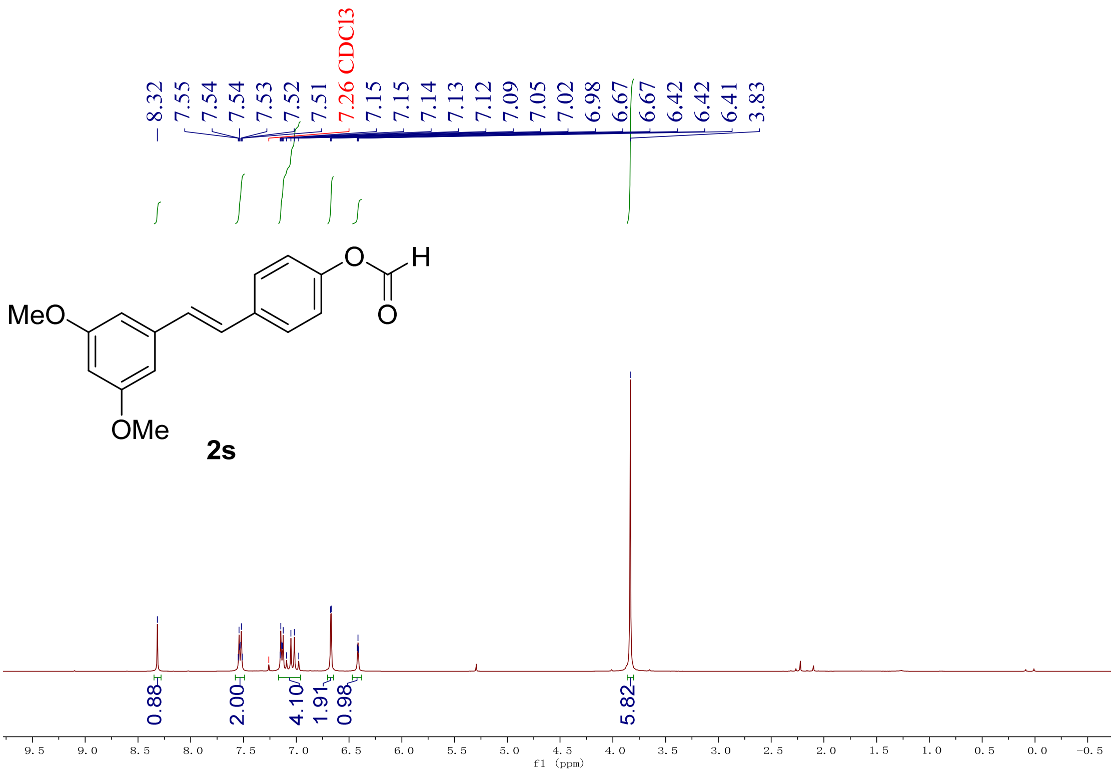

Figure S2. ^{13}C NMR spectrum of **2s** (100 MHz, $CDCl_3$)

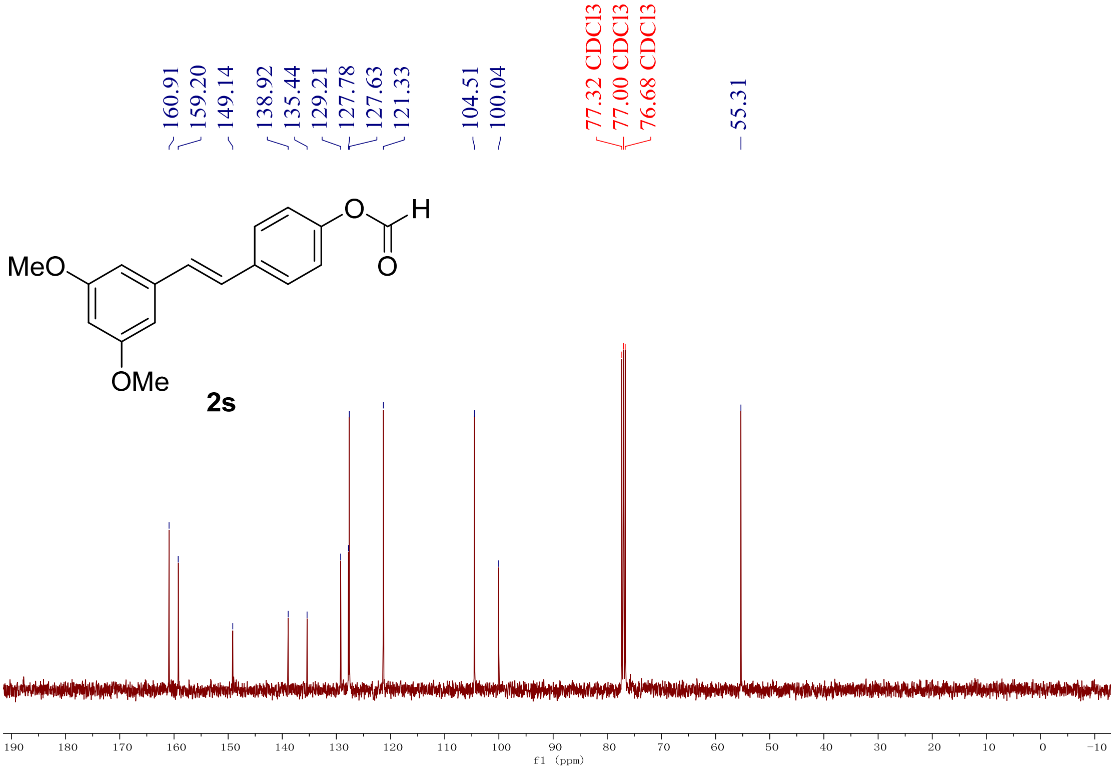

Figure S3. ^{1}H NMR spectrum of **3a** (400 MHz, $CDCl_3$)

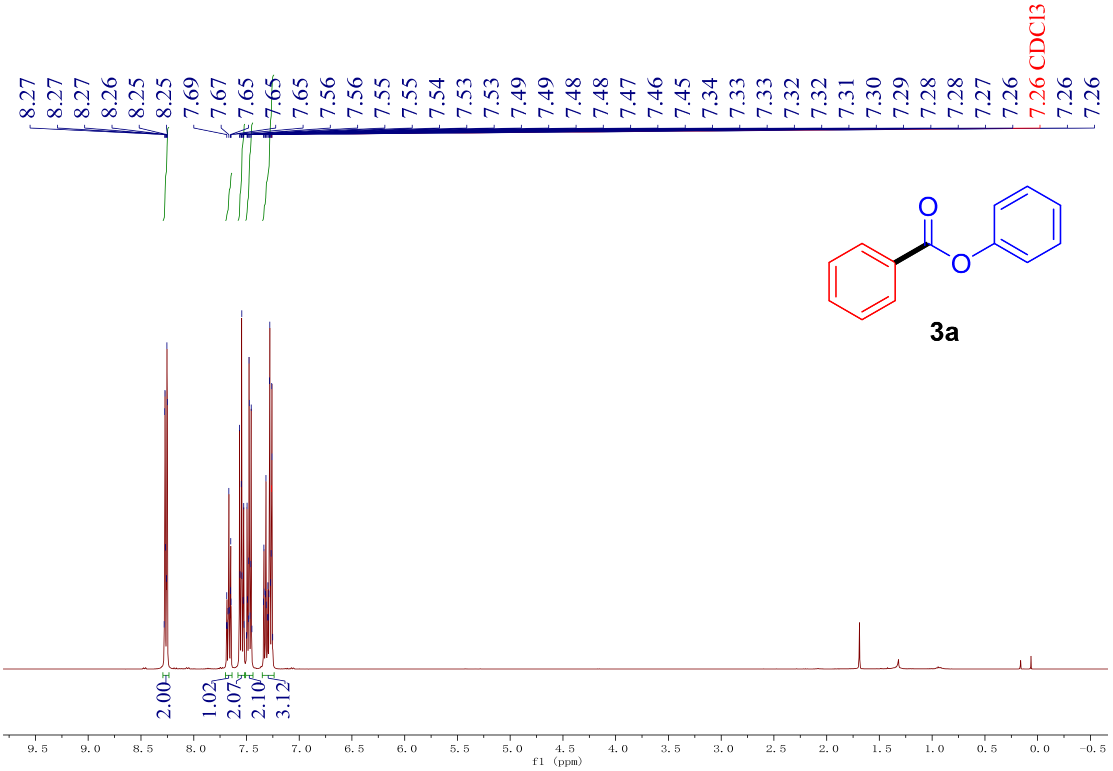

Figure S4. ^{13}C NMR spectrum of **3a** (100 MHz, $CDCl_3$)

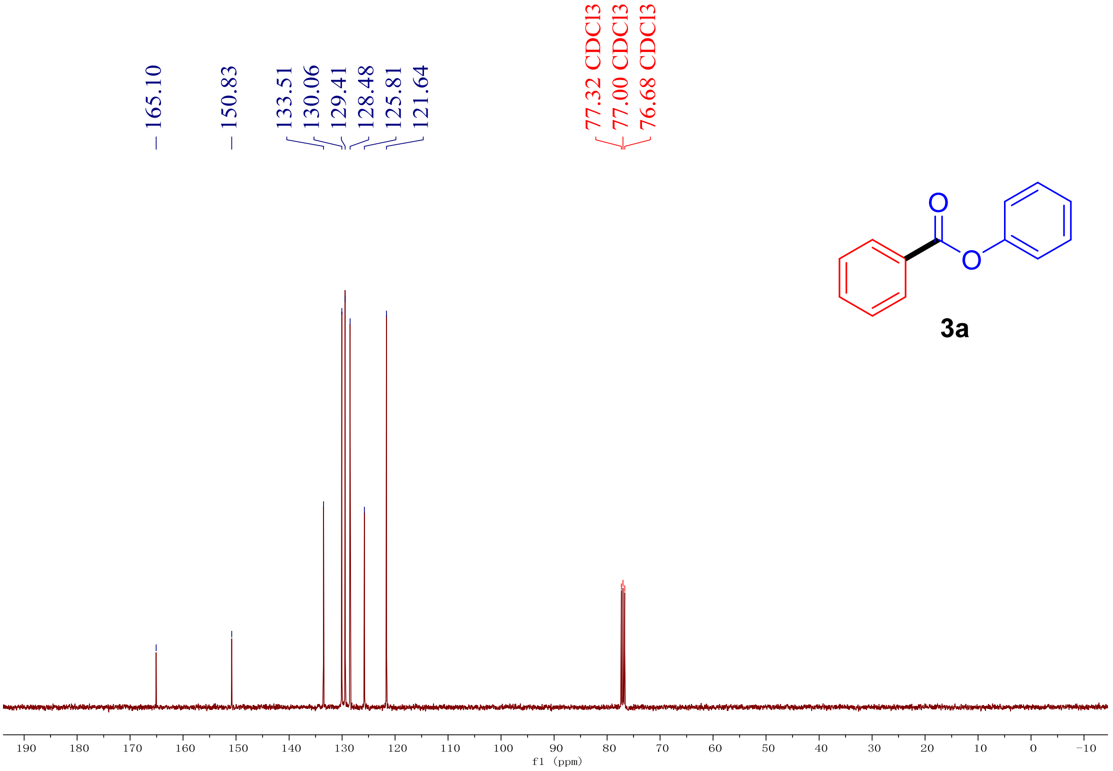

Figure S5. ^{1}H NMR spectrum of **3b** (400 MHz, $CDCl_3$)

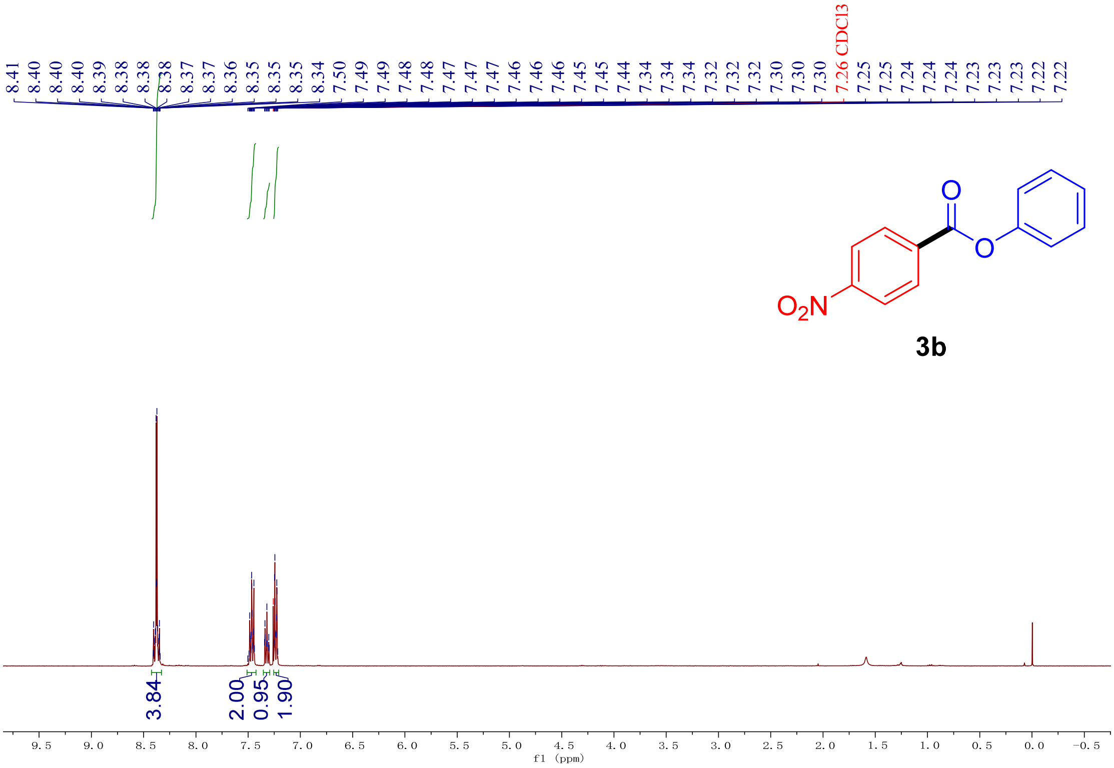

Figure S6. ^{13}C NMR spectrum of **3b** (100 MHz, $CDCl_3$)

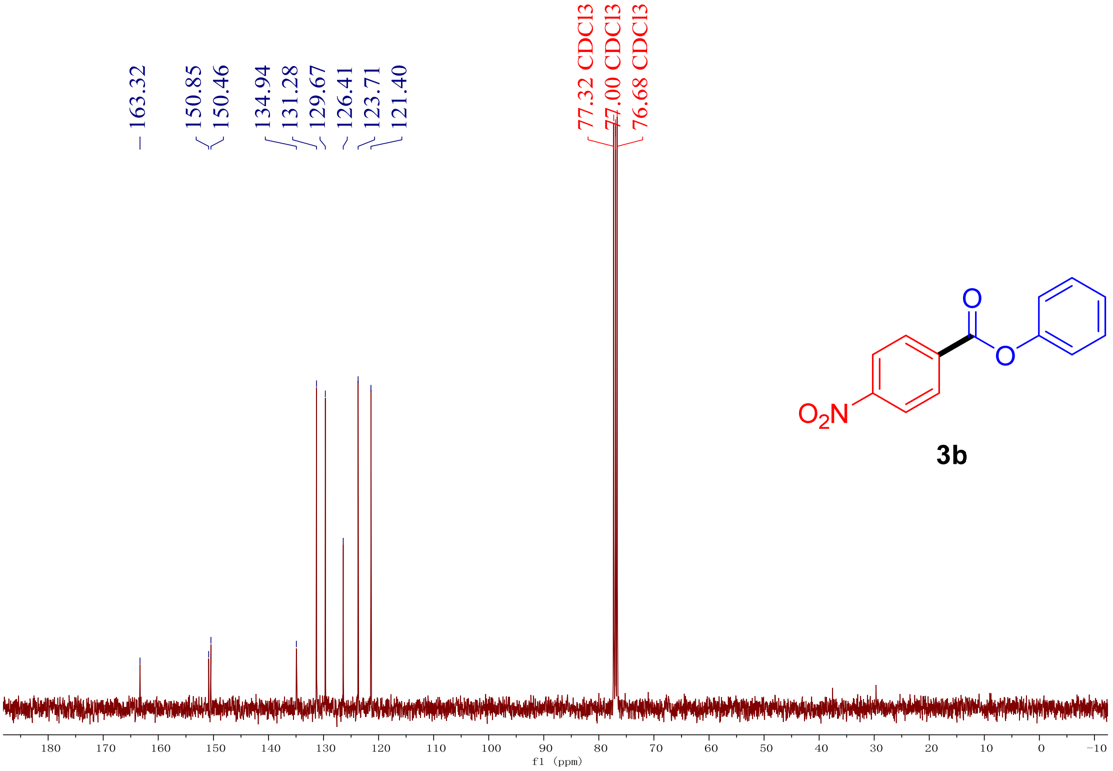

Figure S7.^{1}H NMR spectrum of **3c** (400 MHz, $CDCl_3$)

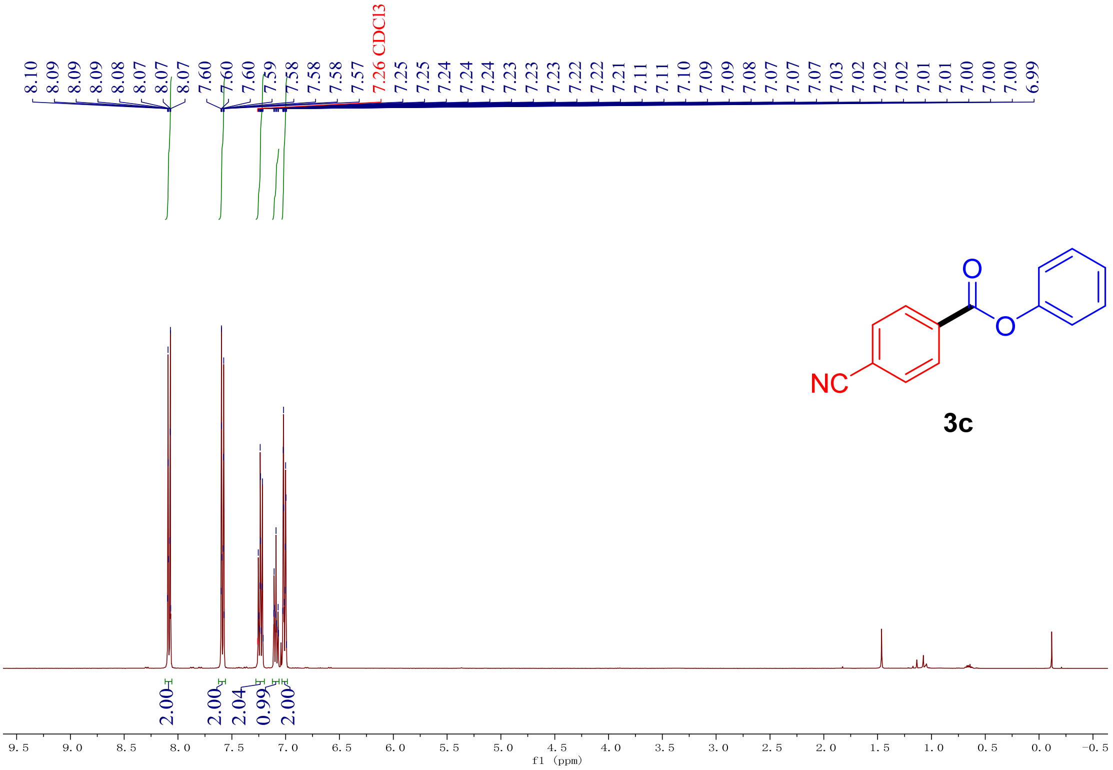

Figure S8. ^{13}C NMR spectrum of **3c** (100 MHz, $CDCl_3$)

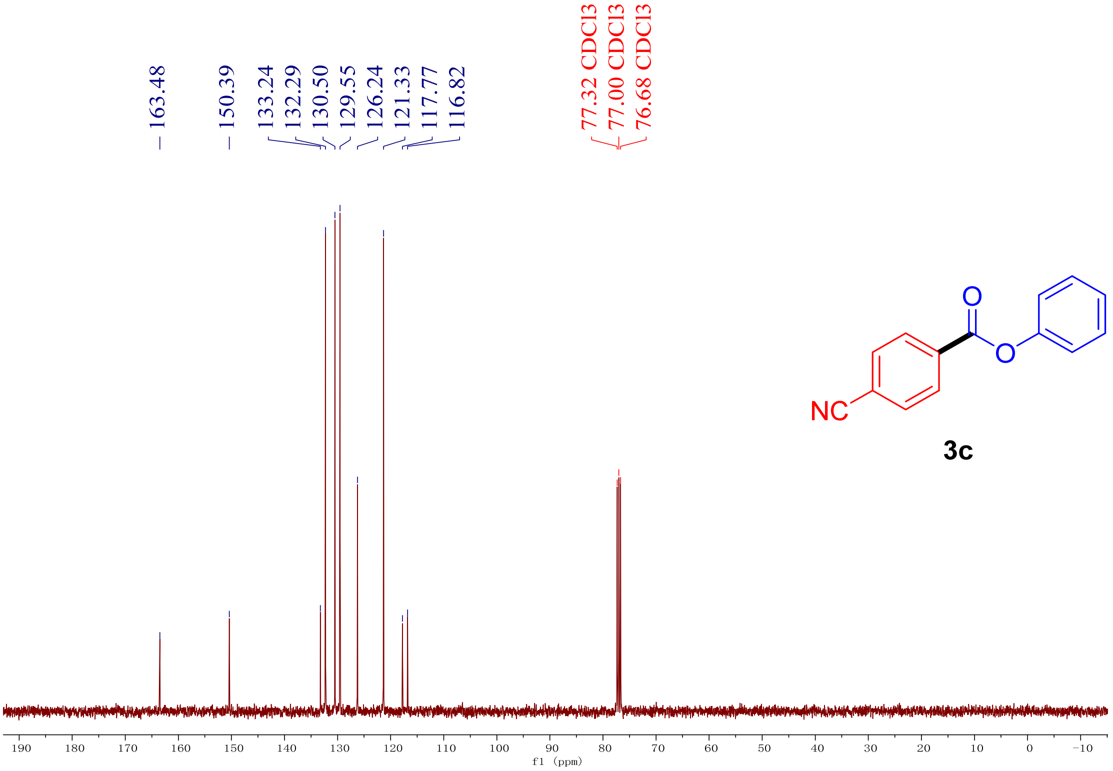

Figure S9. ^{1}H NMR spectrum of **3d** (400 MHz, $CDCl_3$)

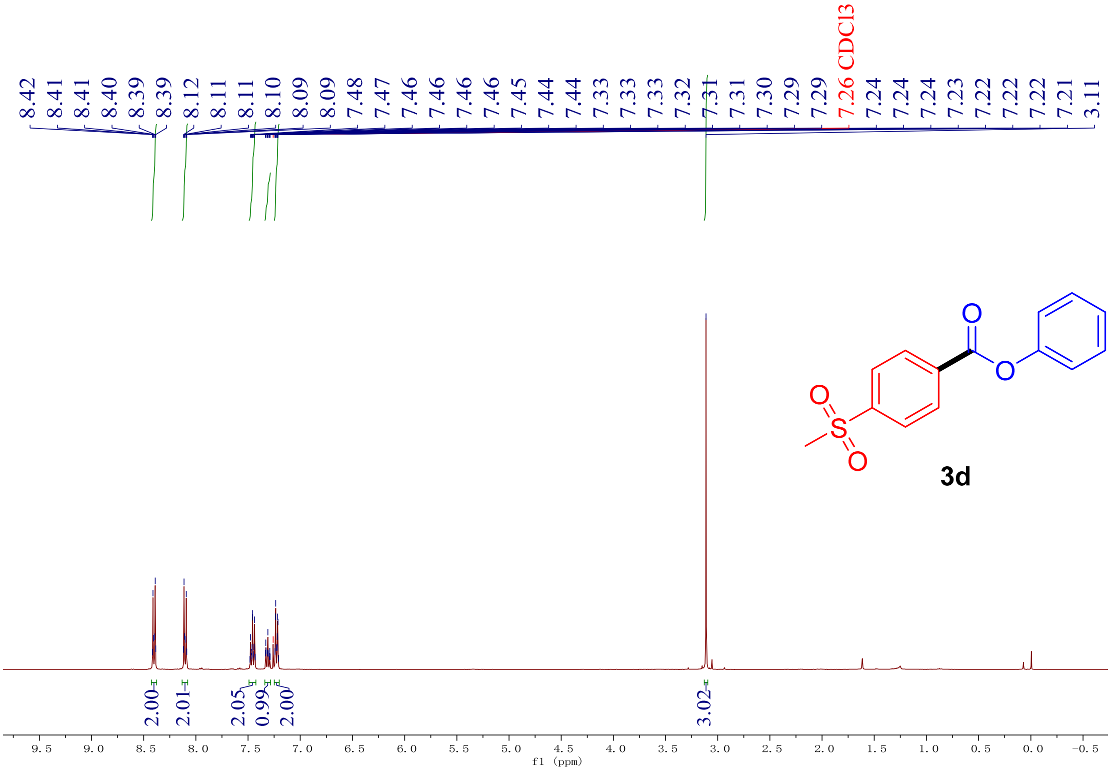

Figure S10. ^{13}C NMR spectrum of **3d** (100 MHz, $CDCl_3$)

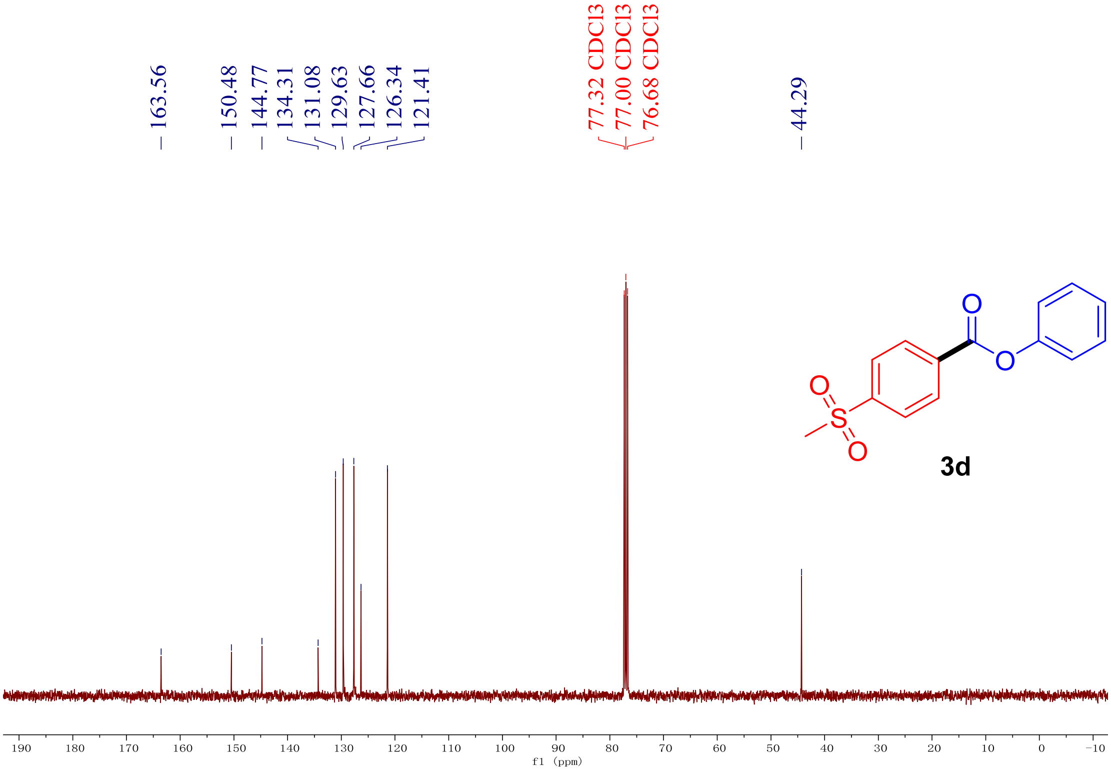

Figure S11. ^{1}H NMR spectrum of **3e** (400 MHz, $CDCl_3$)

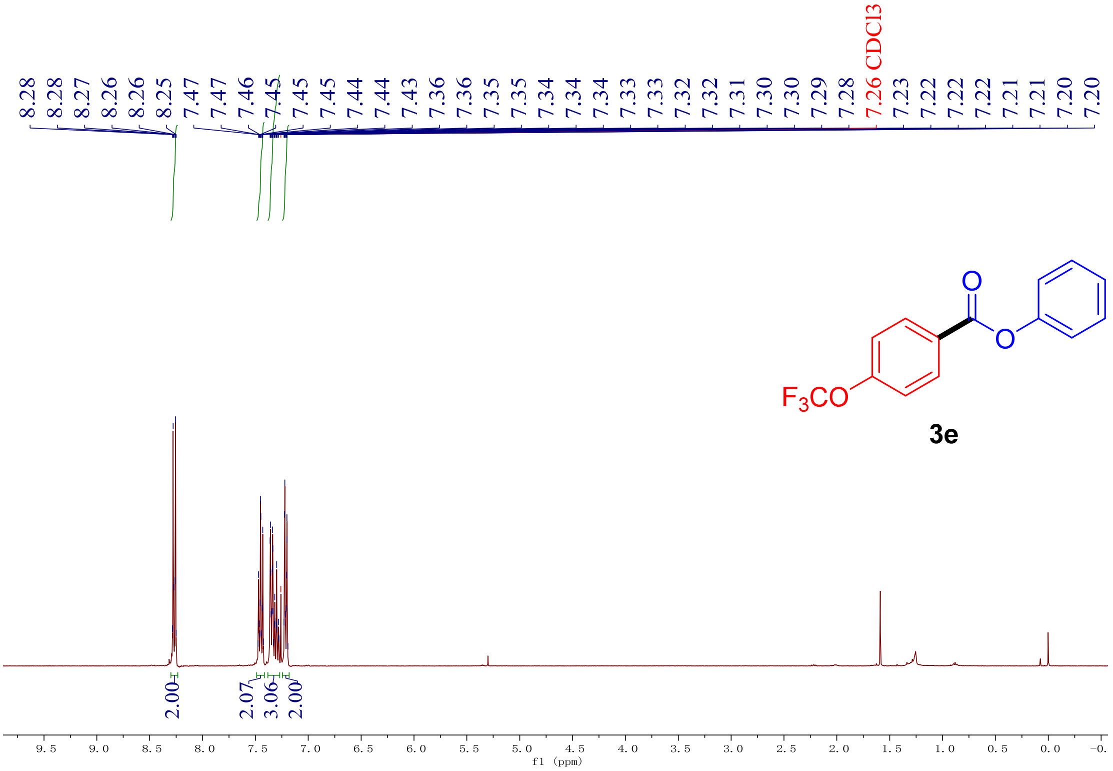

Figure S12. ^{13}C NMR spectrum of **3e** (100 MHz, $CDCl_3$)

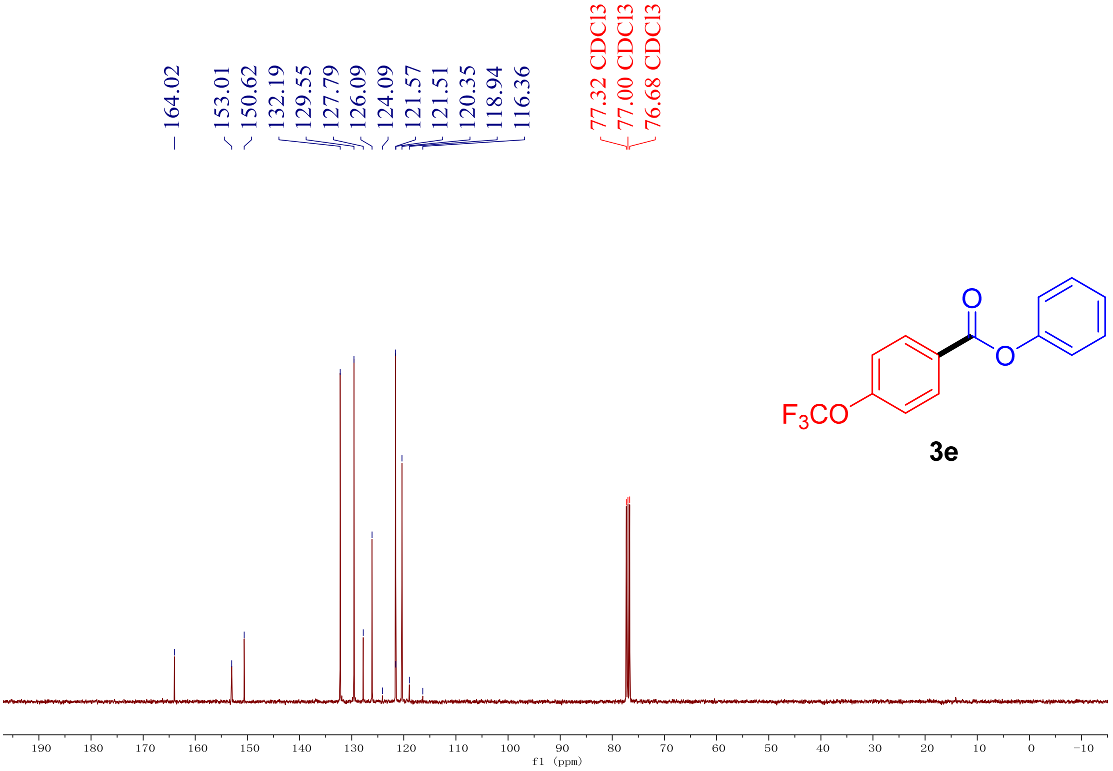

Figure S13. ^{19}F NMR spectrum of **3e** (376 MHz, $CDCl_3$)

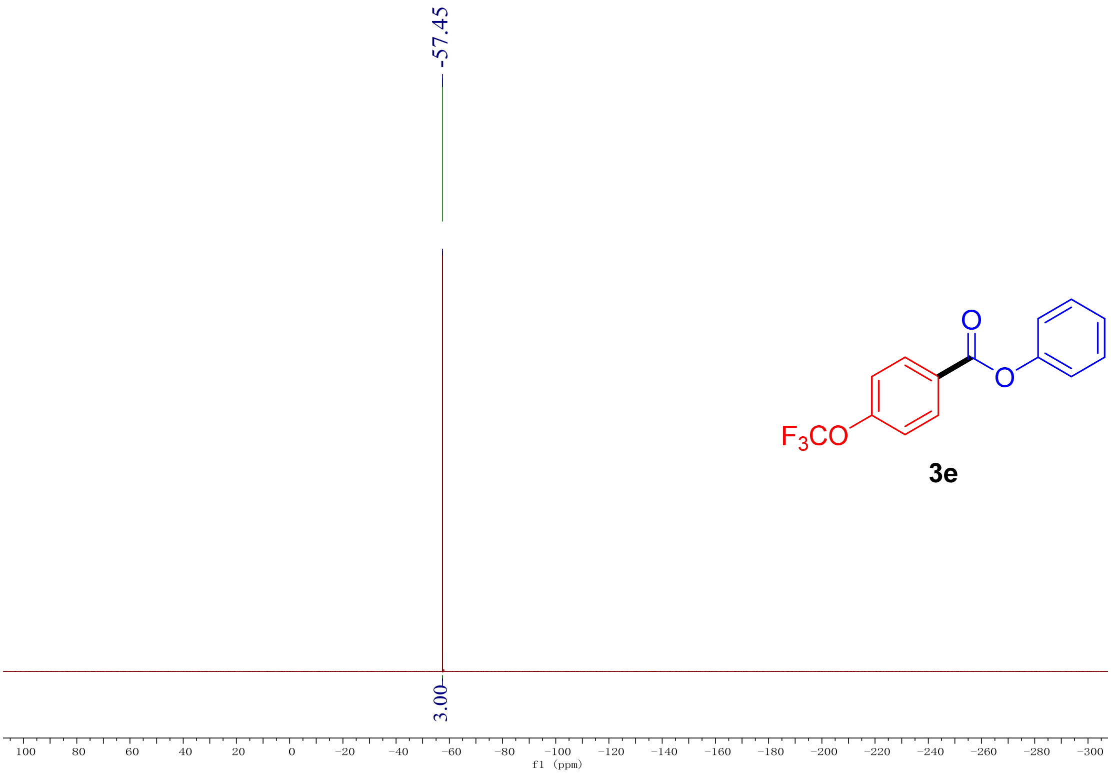

Figure S14. ^{1}H NMR spectrum of **3f** (400 MHz, $CDCl_3$)

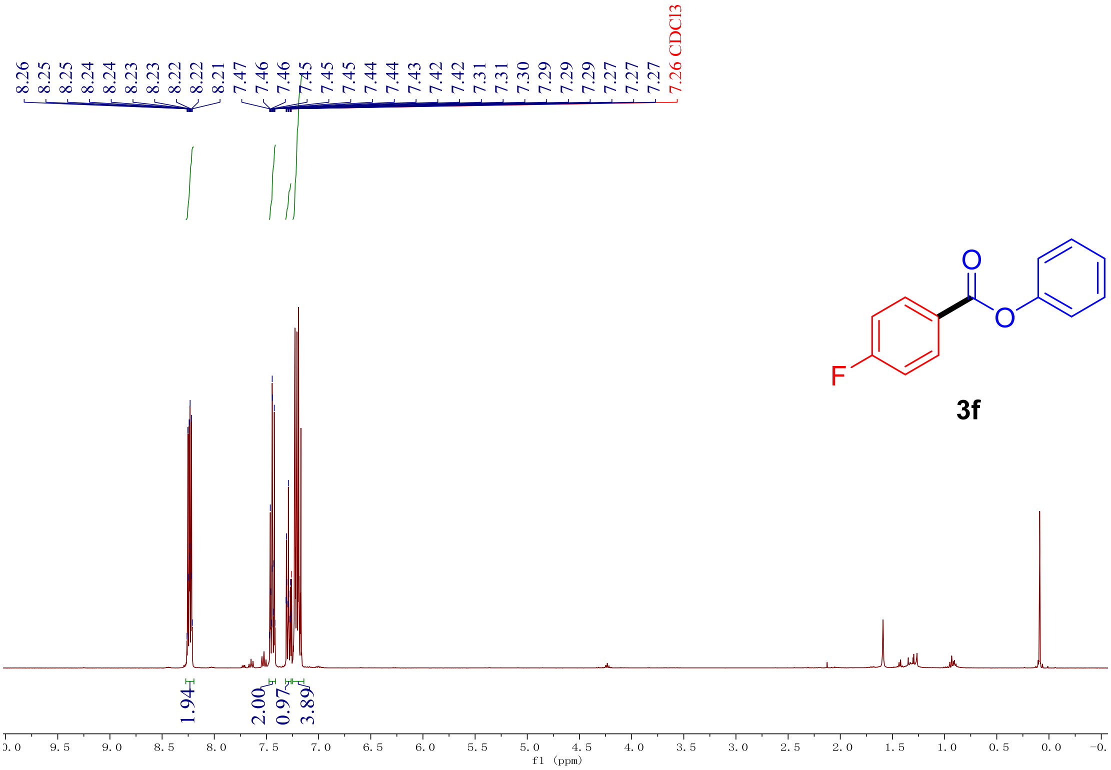

Figure S15. ^{13}C NMR spectrum of **3f** (100 MHz, $CDCl_3$)

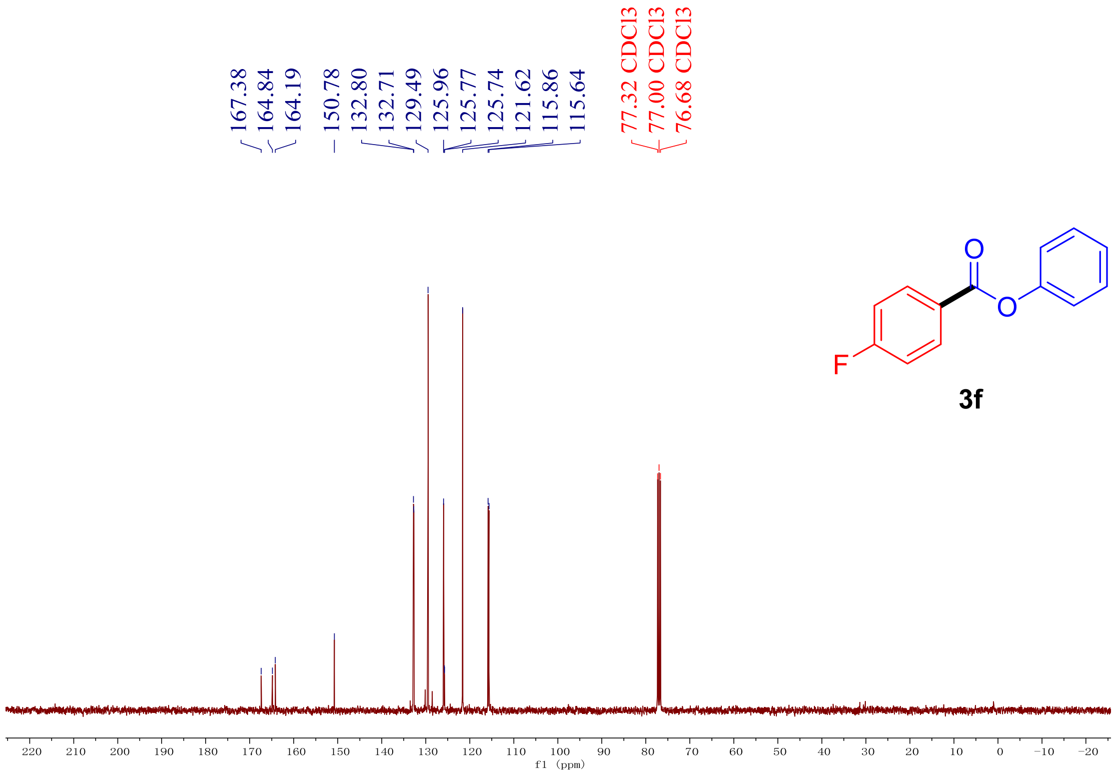

Figure S16. ^{19}F NMR spectrum of **3f** (376 MHz, $CDCl_3$)

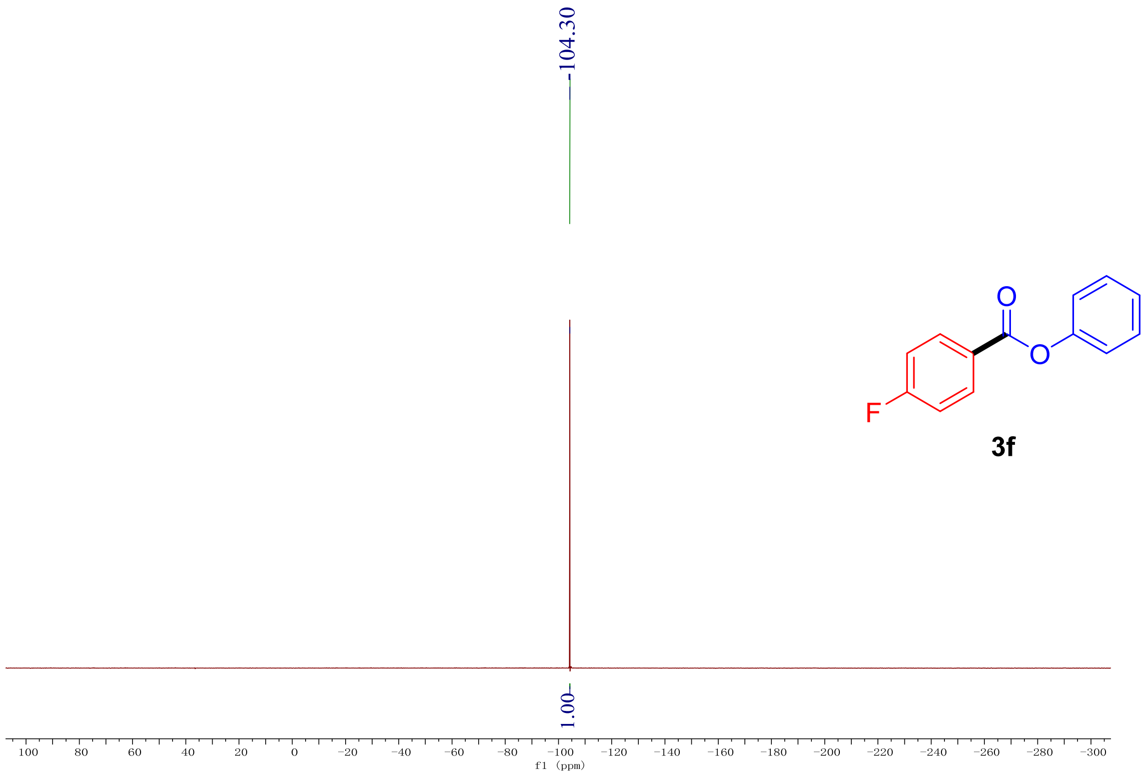

Figure S17. ^{1}H NMR spectrum of **3g** (400 MHz, $CDCl_3$)

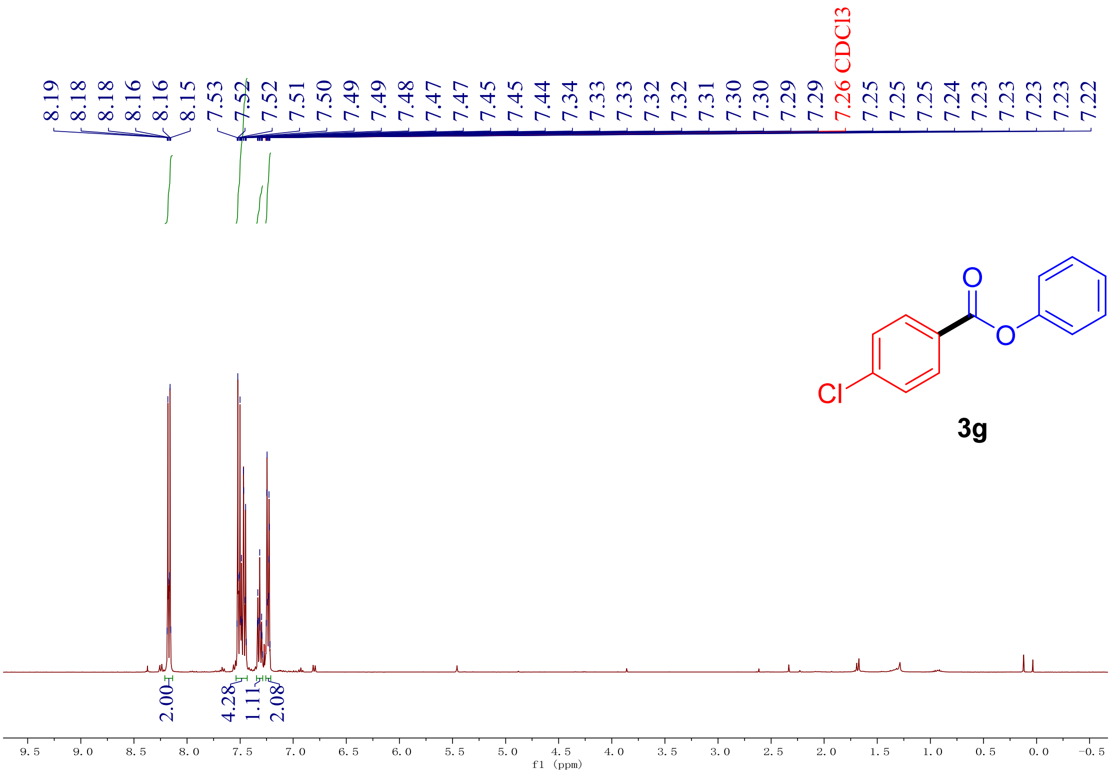

Figure S18. ^{13}C NMR spectrum of **3g** (100 MHz, $CDCl_3$)

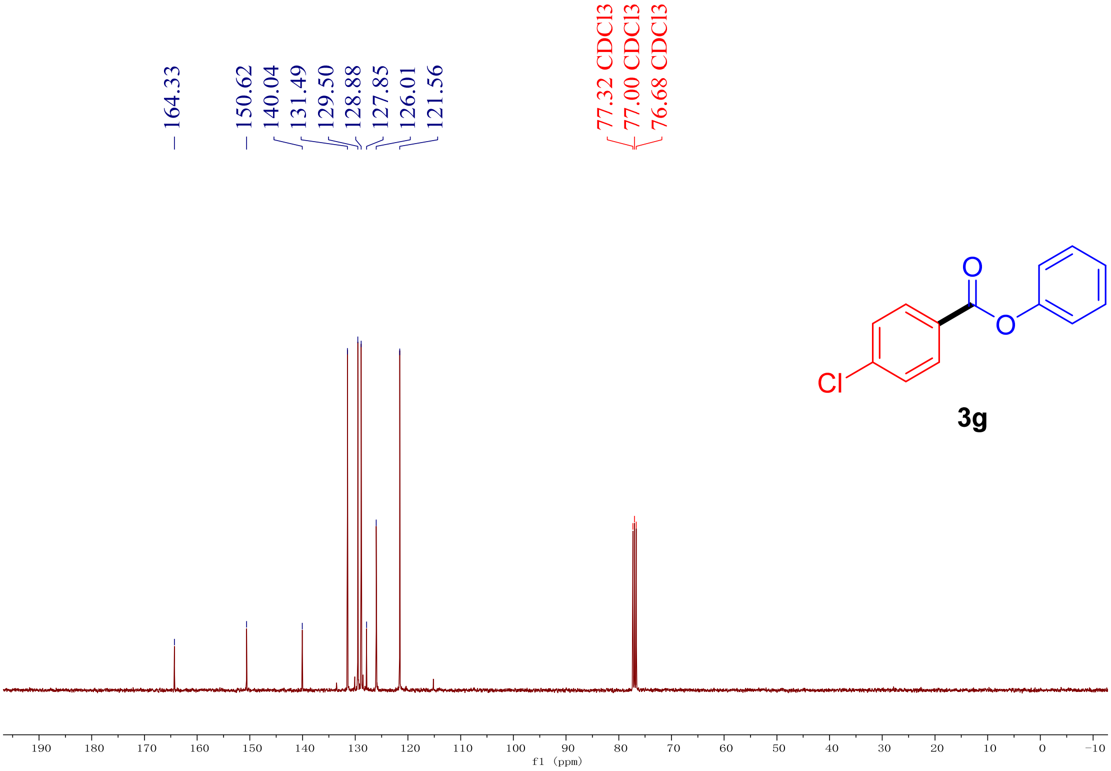

Figure S19. ^{1}H NMR spectrum of **3h** (400 MHz, $CDCl_3$)

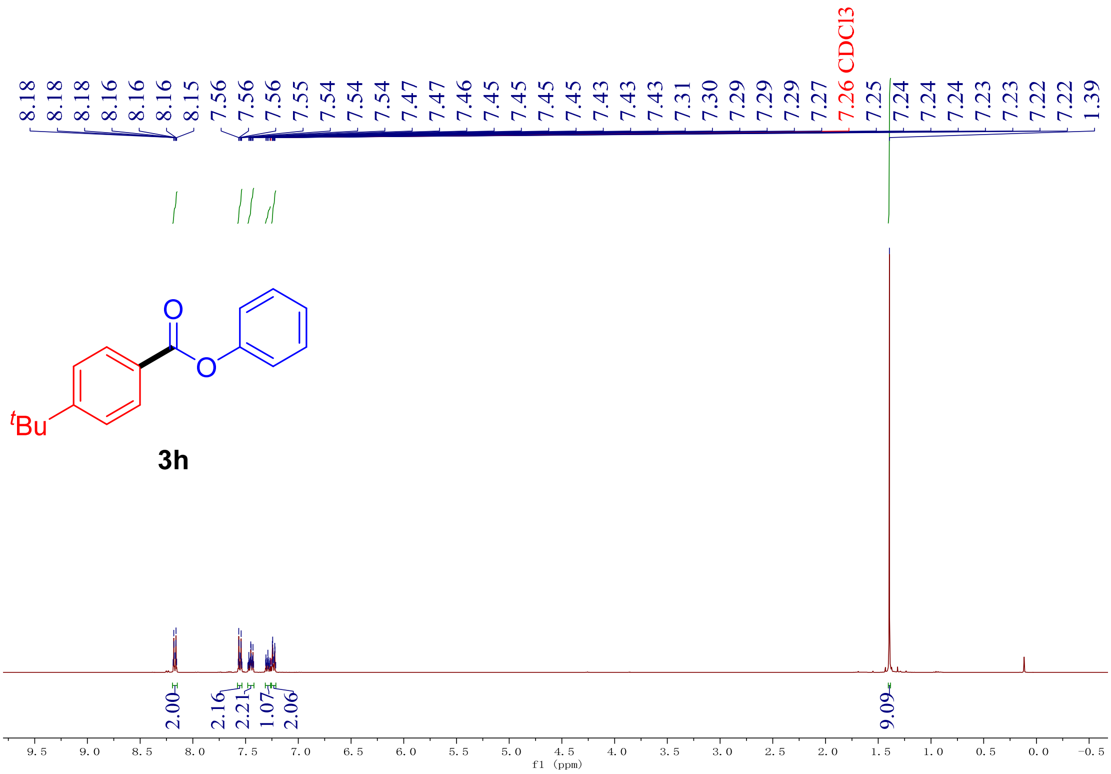

Figure S20. ^{13}C NMR spectrum of **3h** (100 MHz, $CDCl_3$)

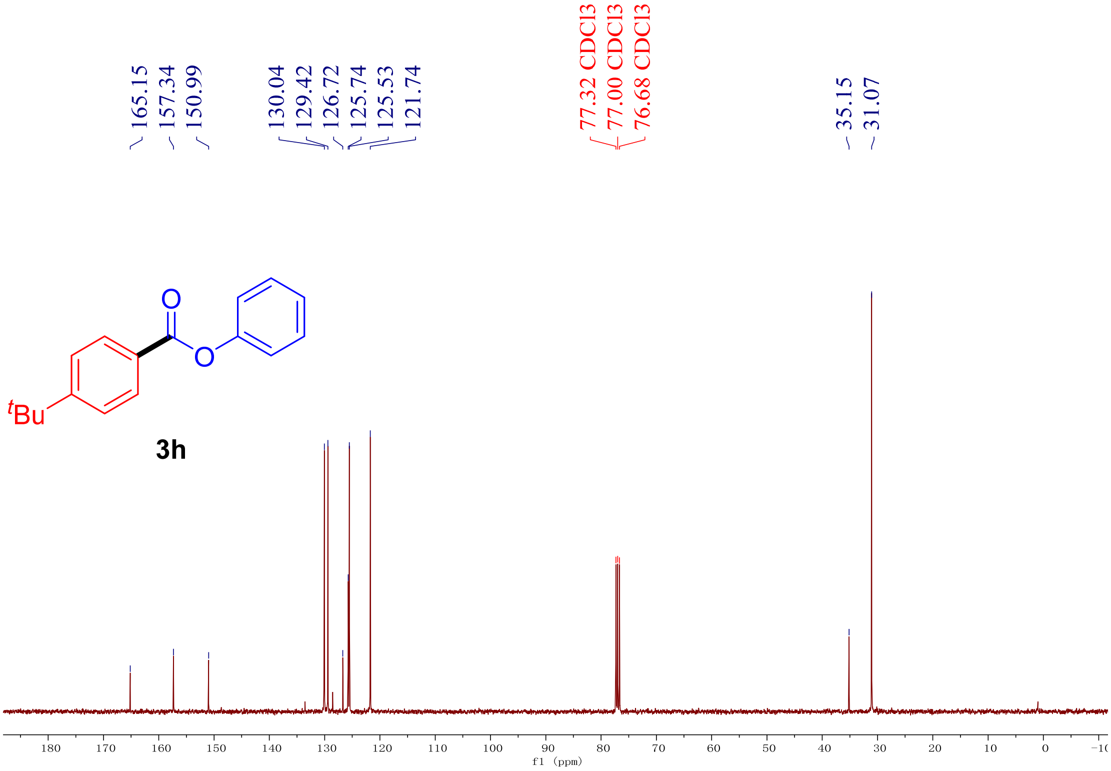

Figure S21. ^{1}H NMR spectrum of **3i** (400 MHz, $CDCl_3$)

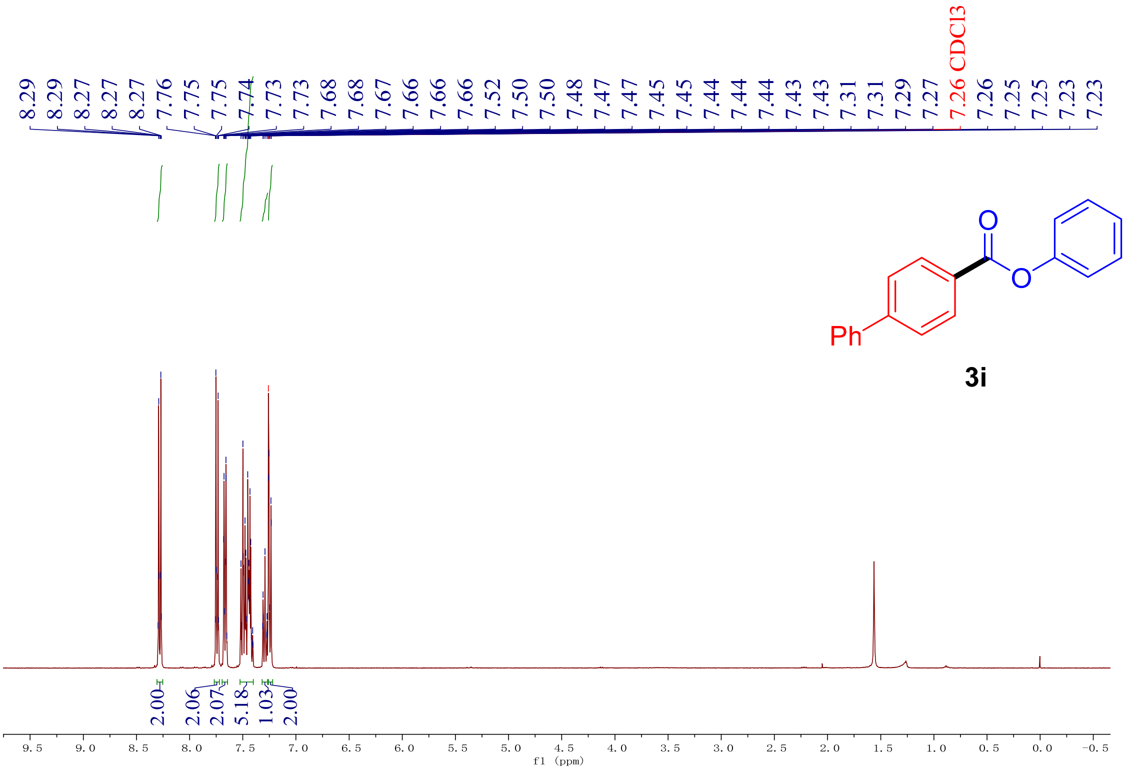

Figure S22. ^{13}C NMR spectrum of **3i** (100 MHz, $CDCl_3$)

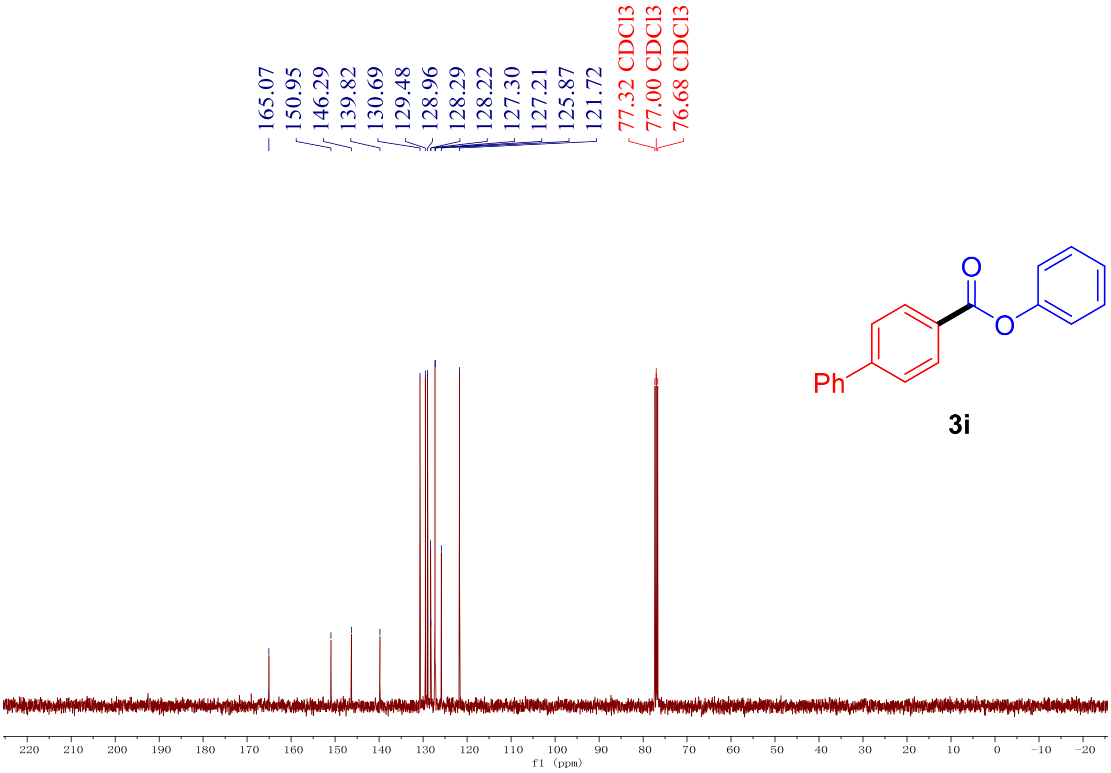

Figure S23. ^{1}H NMR spectrum of **3j** (400 MHz, $CDCl_3$)

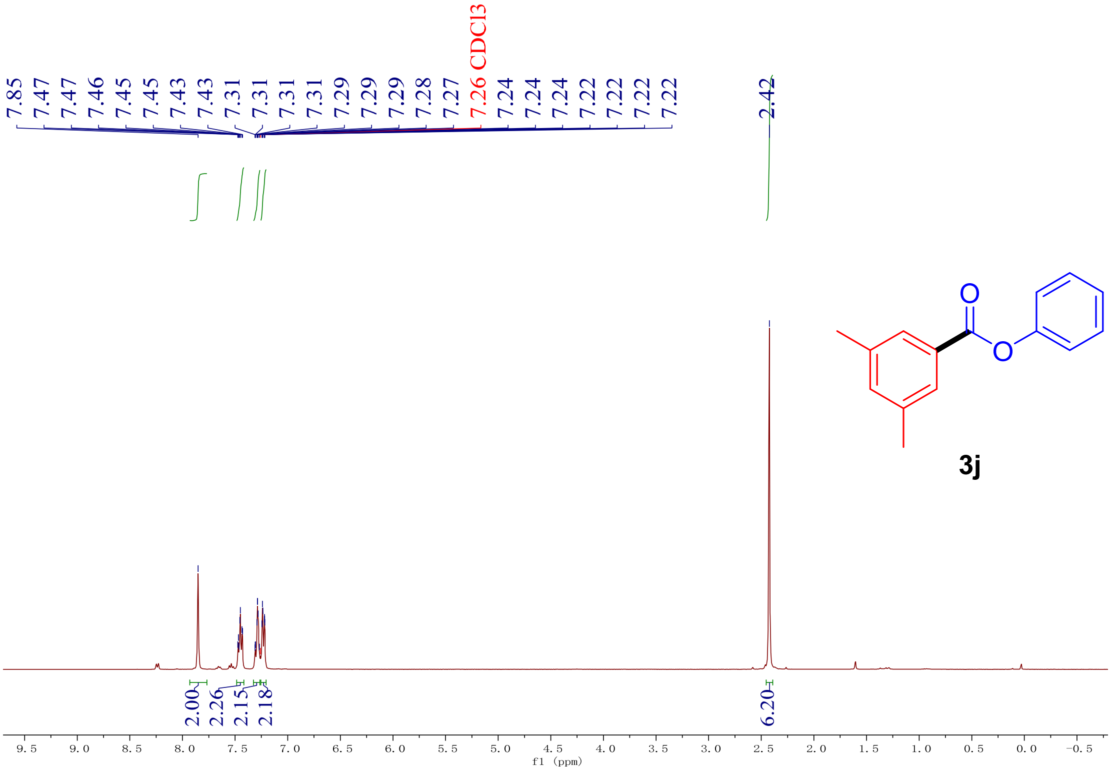

Figure S24. ^{13}C NMR spectrum of **3j** (100 MHz, $CDCl_3$)

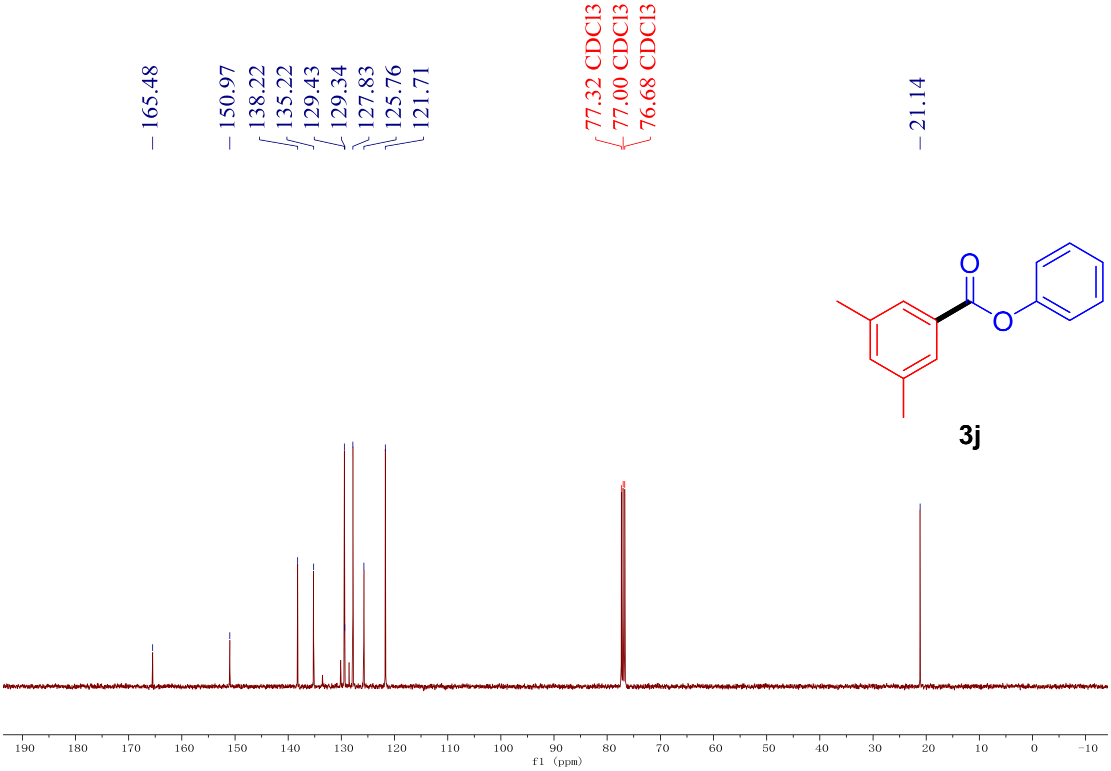

Figure S25. ^{1}H NMR spectrum of **3k** (400 MHz, $CDCl_3$)

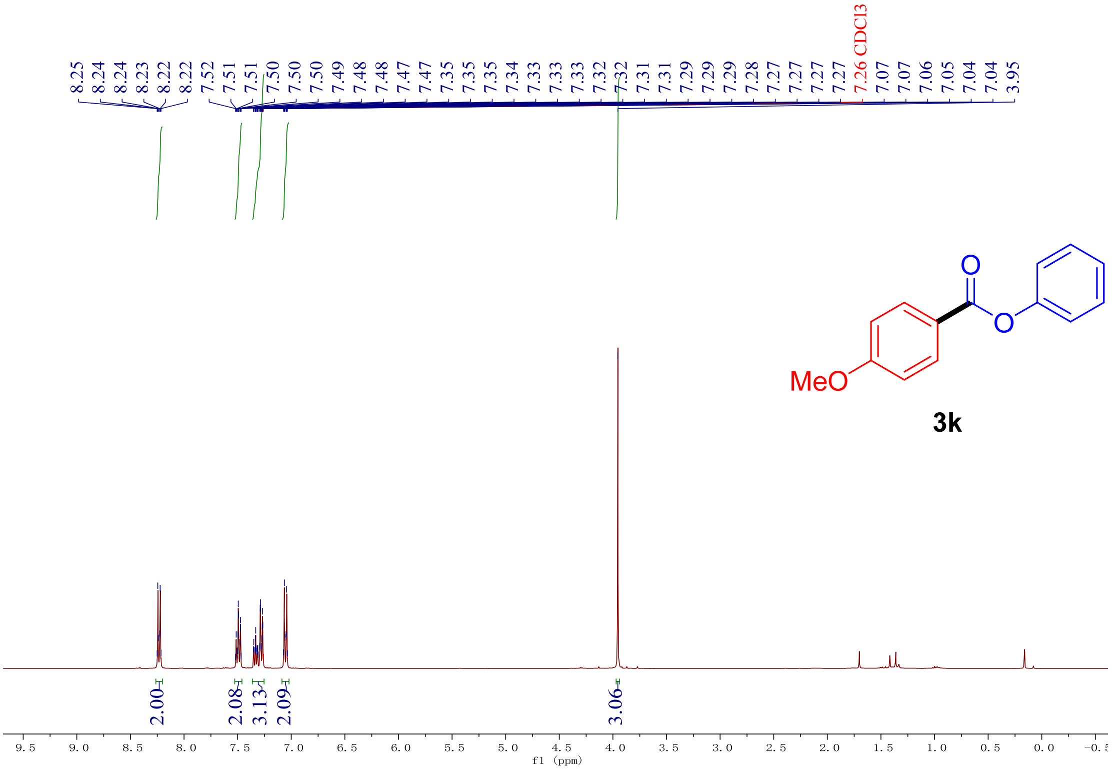

Figure S26. ^{13}C NMR spectrum of **3k** (100 MHz, $CDCl_3$)

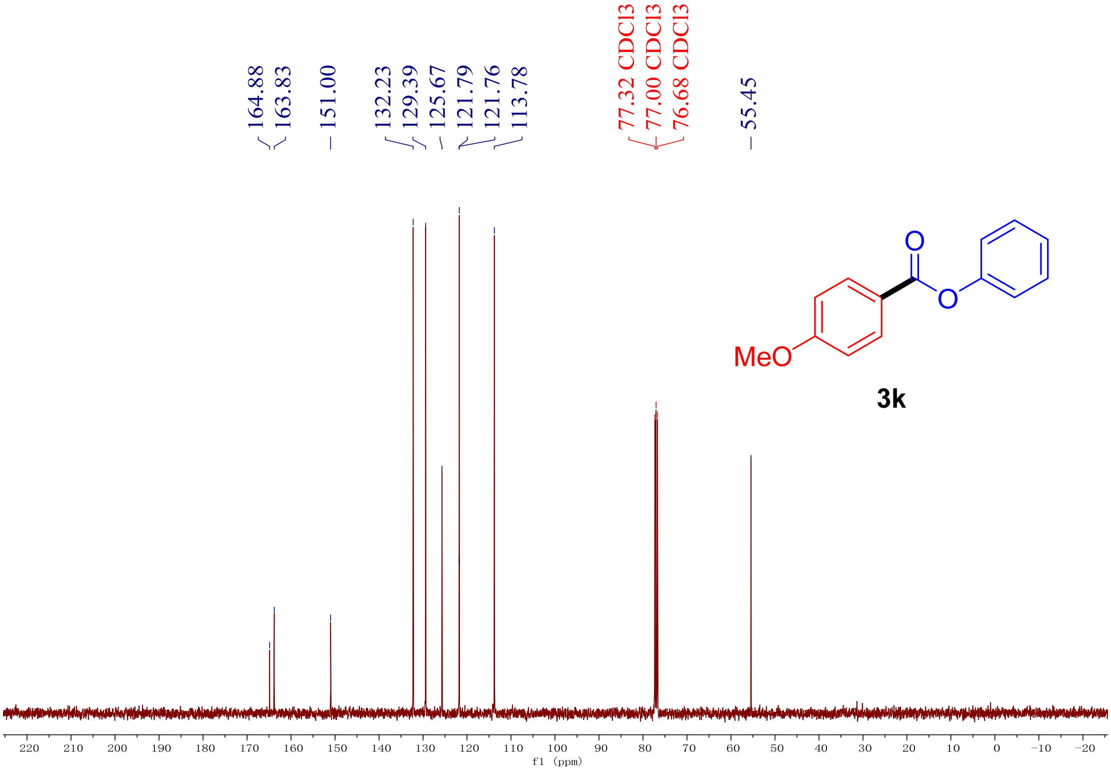

Figure S27. ^{1}H NMR spectrum of **3l** (400 MHz, $CDCl_3$)

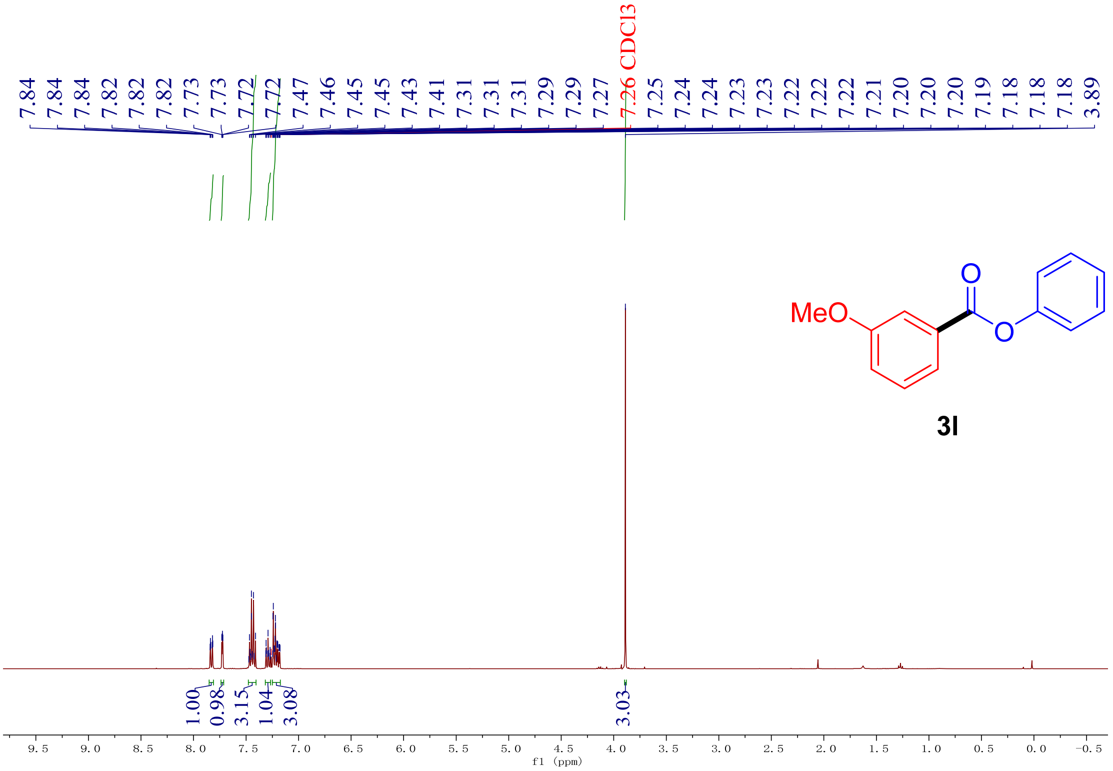

Figure S28. ^{13}C NMR spectrum of **3l** (100 MHz, $CDCl_3$)

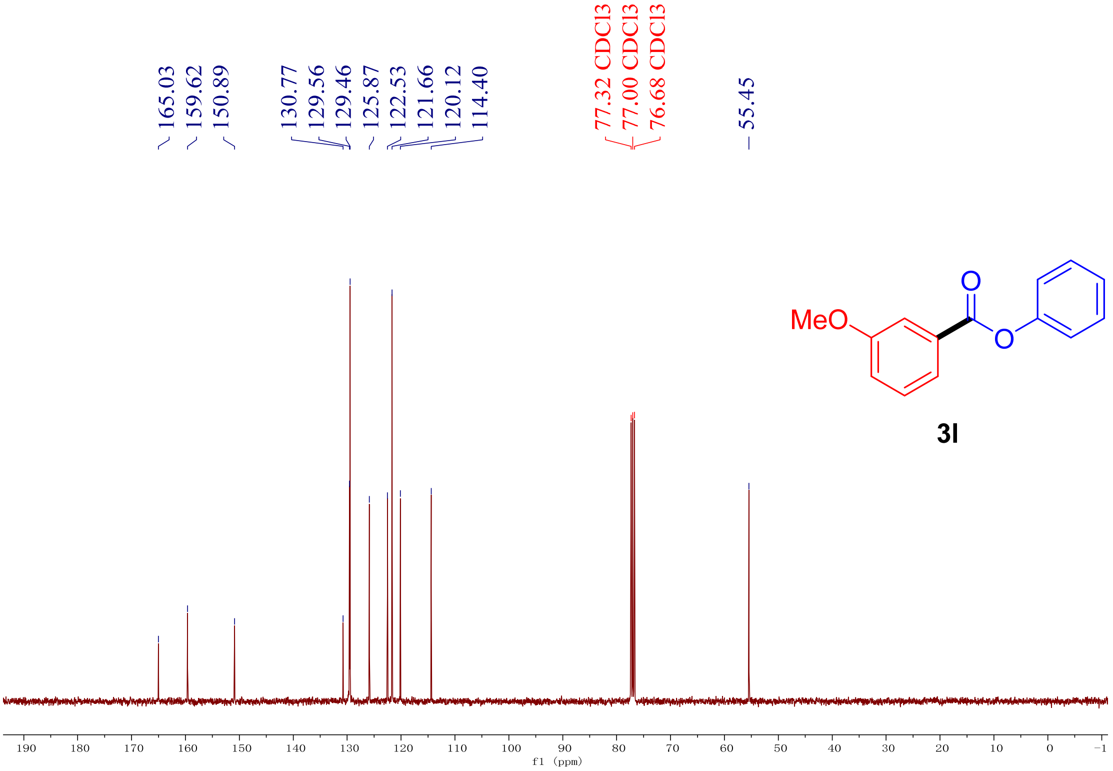

Figure S29. ^{1}H NMR spectrum of **3m** (400 MHz, $CDCl_3$)

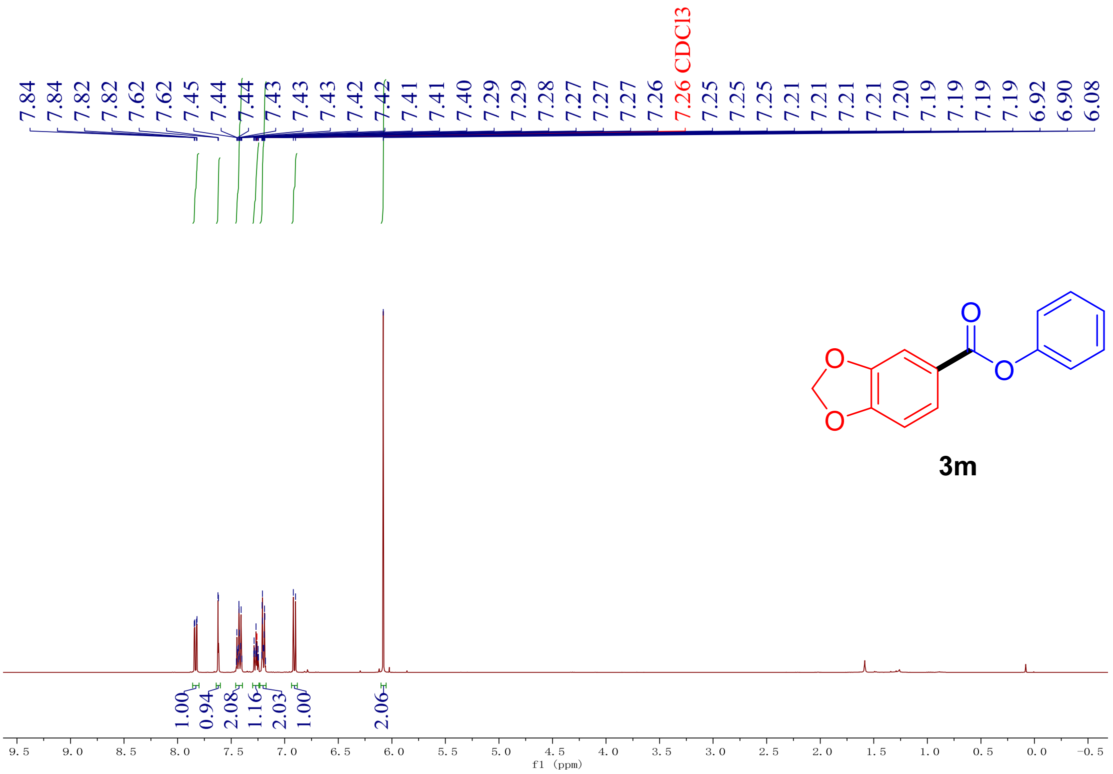

Figure S30. ^{13}C NMR spectrum of **3m** (100 MHz, $CDCl_3$)

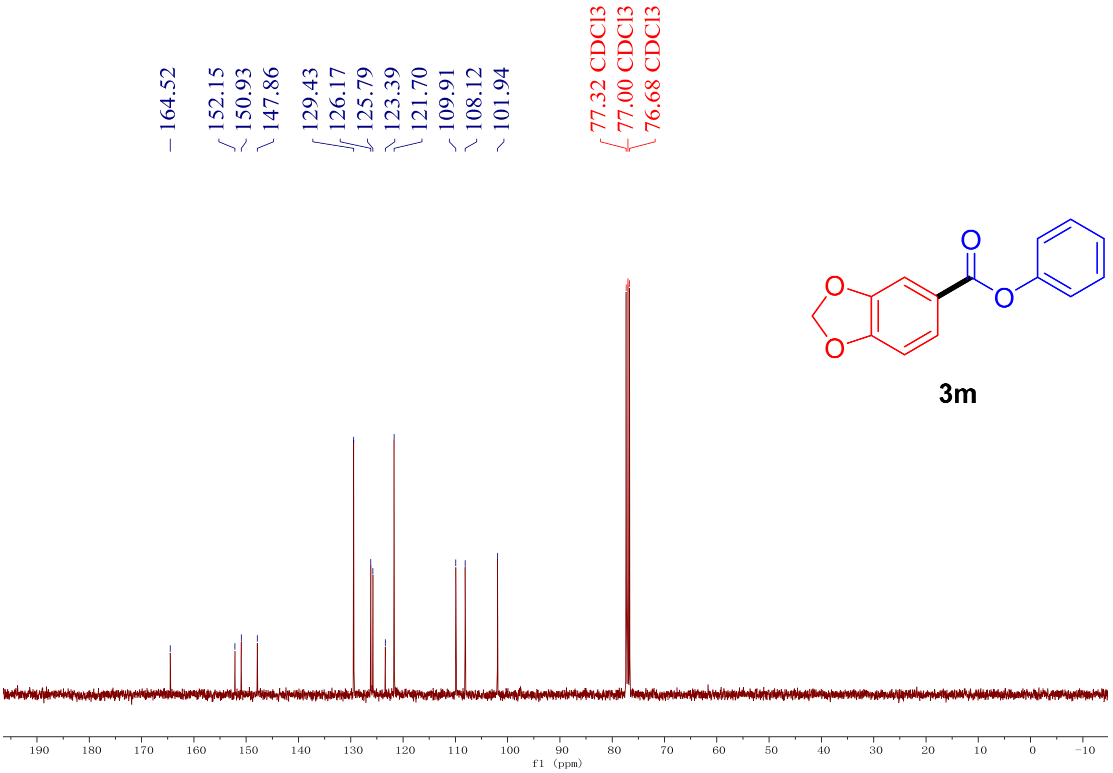

Figure S31. ^1H NMR spectrum of **3n** (400 MHz, $CDCl_3$)

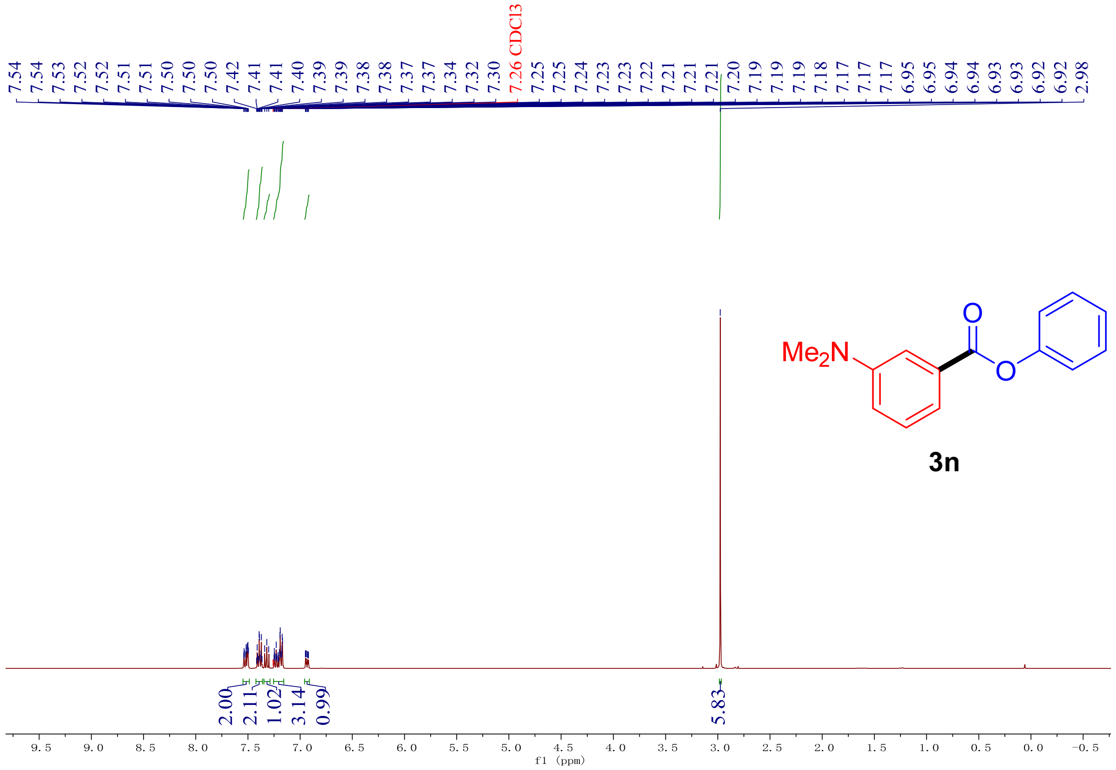

Figure S32. ^{13}C NMR spectrum of **3n** (100 MHz, $CDCl_3$)

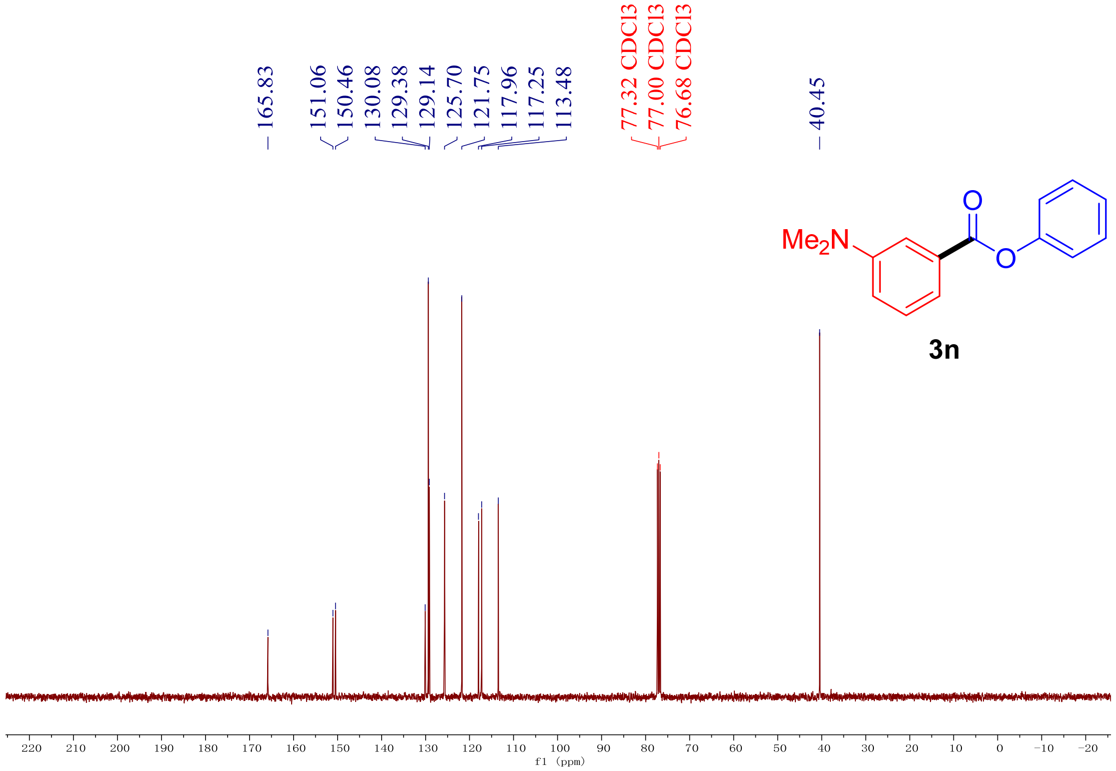

Figure S33. ^{1}H NMR spectrum of **3o** (400 MHz, $CDCl_3$)

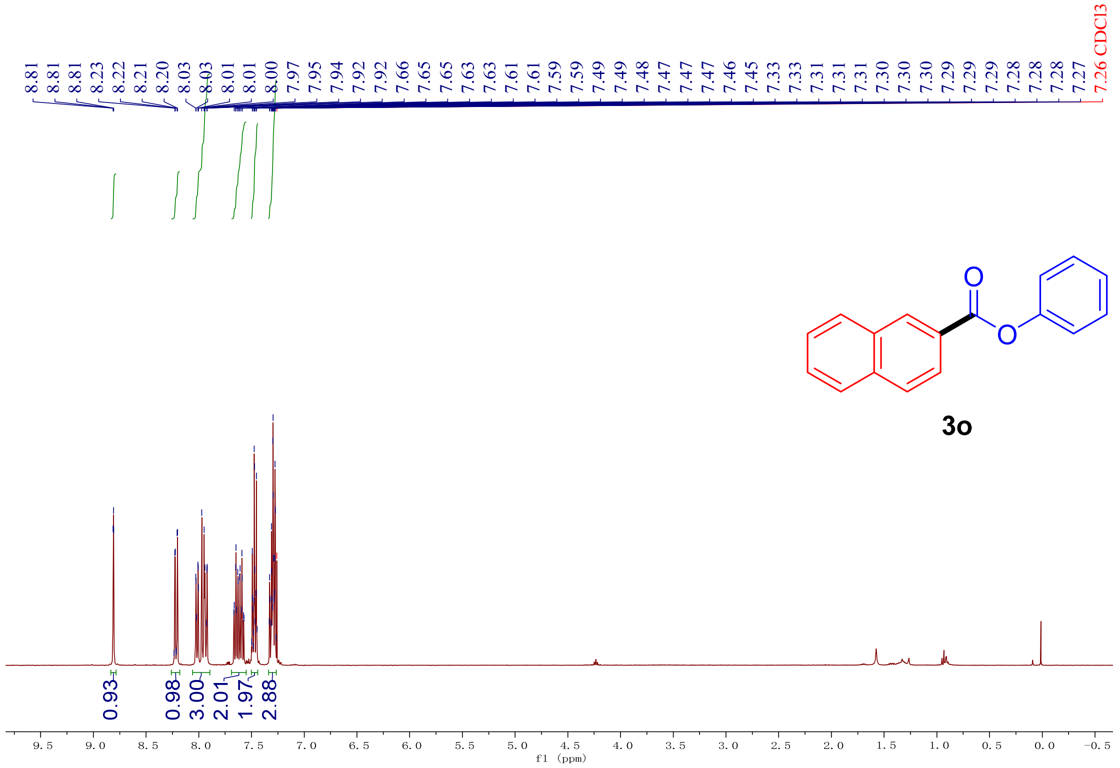

Figure S34. ^{13}C NMR spectrum of **3o** (100 MHz, $CDCl_3$)

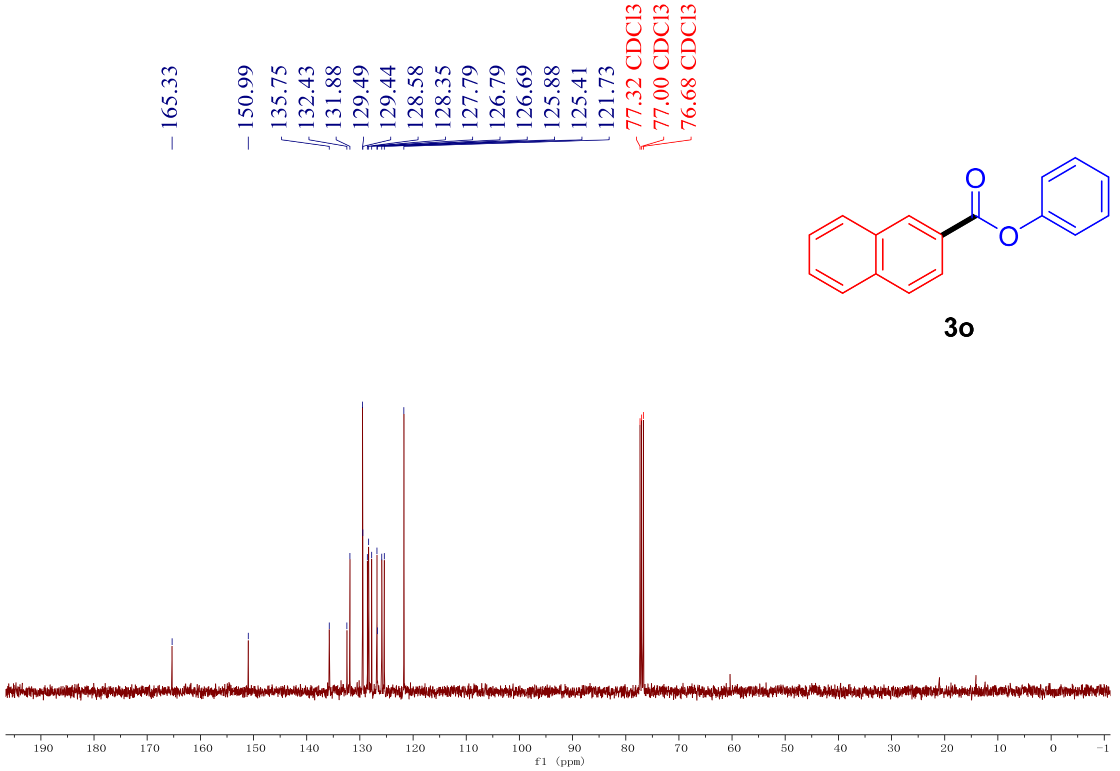

Figure S35. ^{1}H NMR spectrum of **3p** (400 MHz, $CDCl_3$)

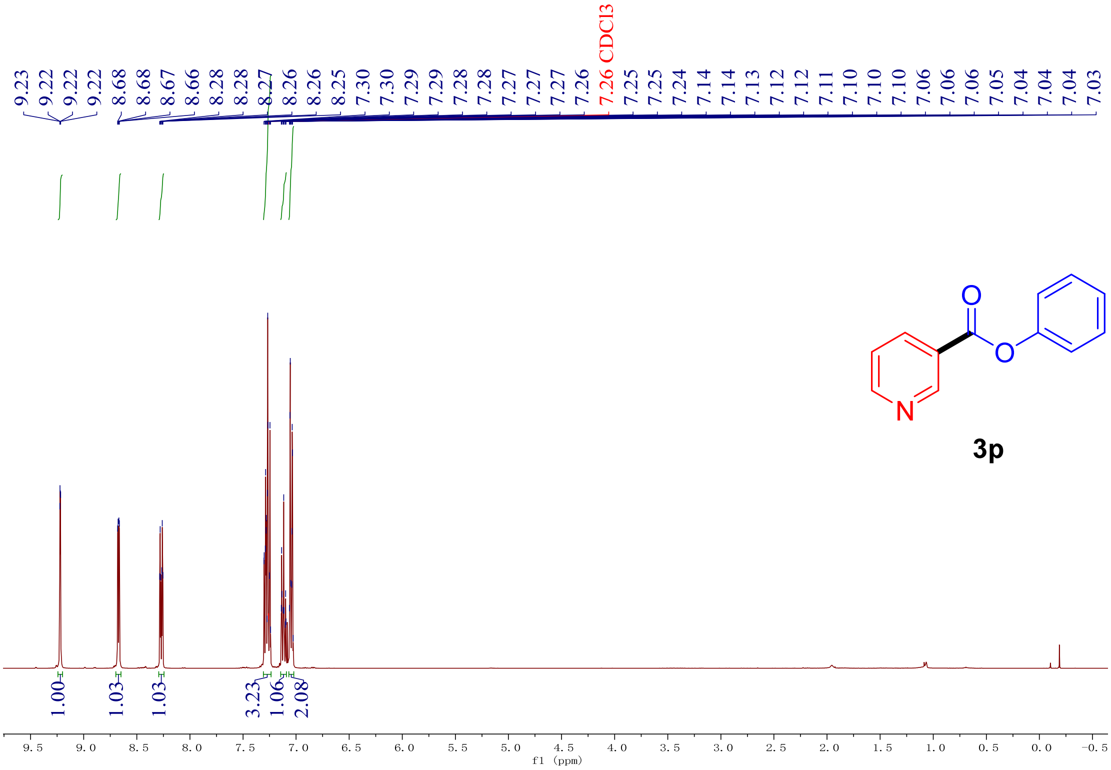

Figure S36. ^{13}C NMR spectrum of **3p** (100 MHz, $CDCl_3$)

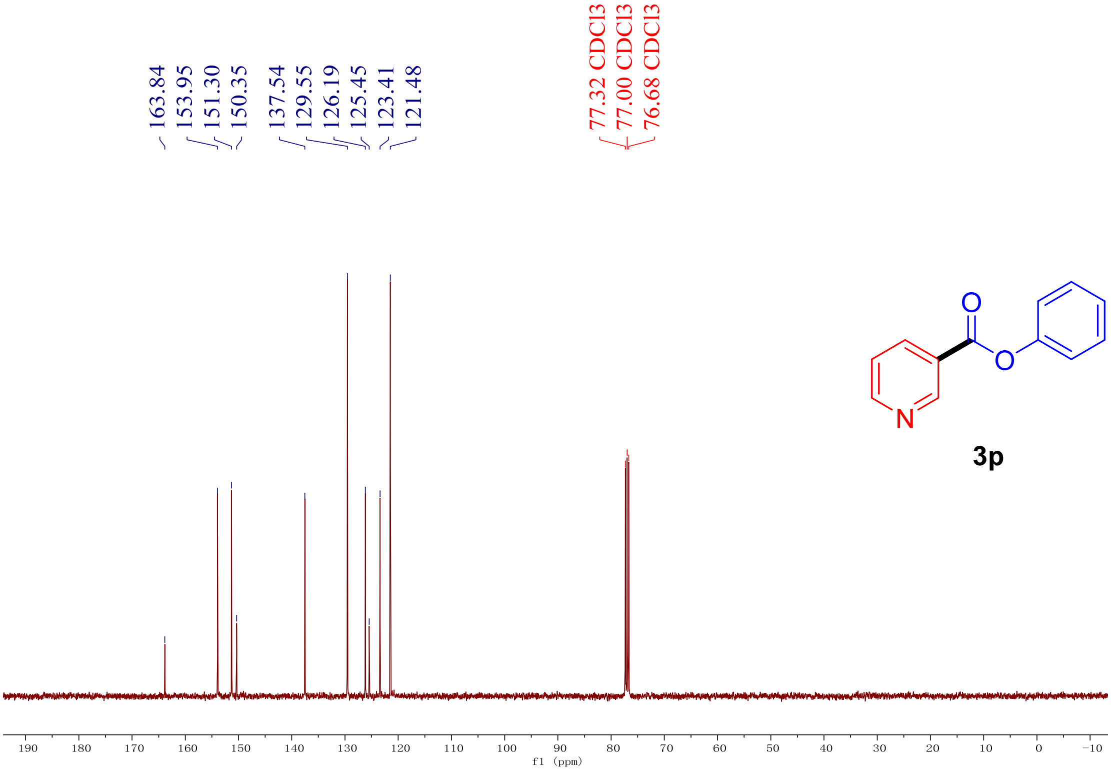

Figure S37. ^{1}H NMR spectrum of **3q** (400 MHz, $CDCl_3$)

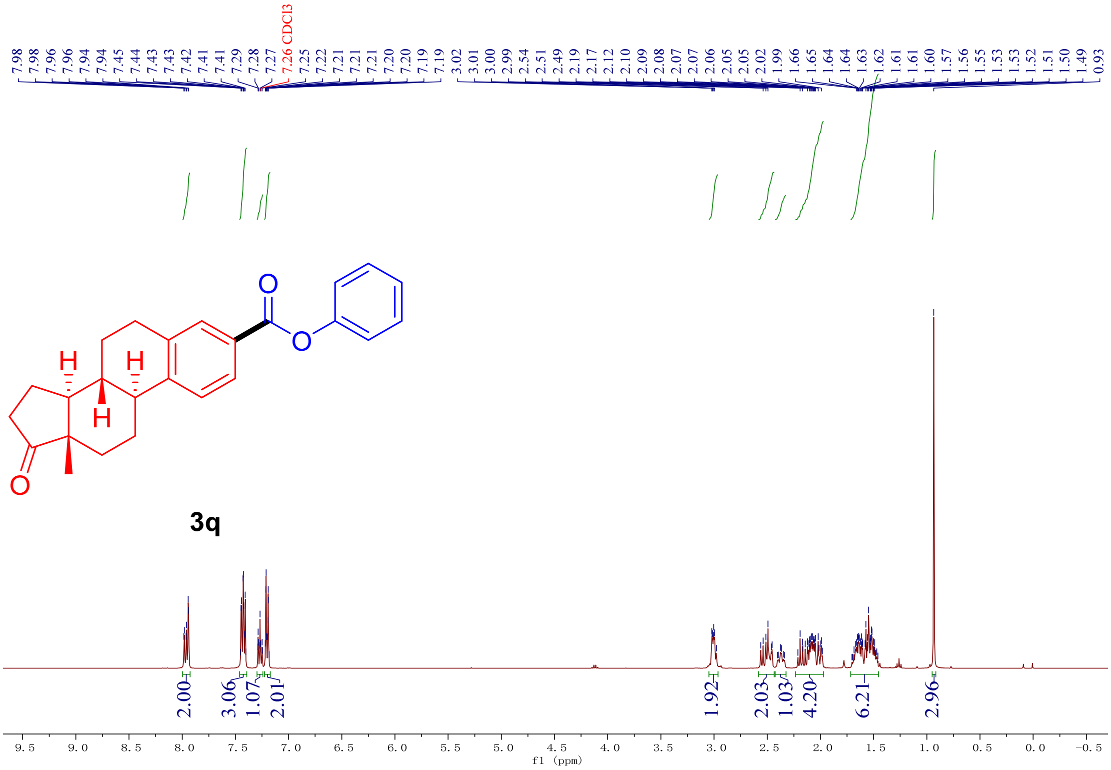

Figure S38. ^{13}C NMR spectrum of **3q** (100 MHz, $CDCl_3$)

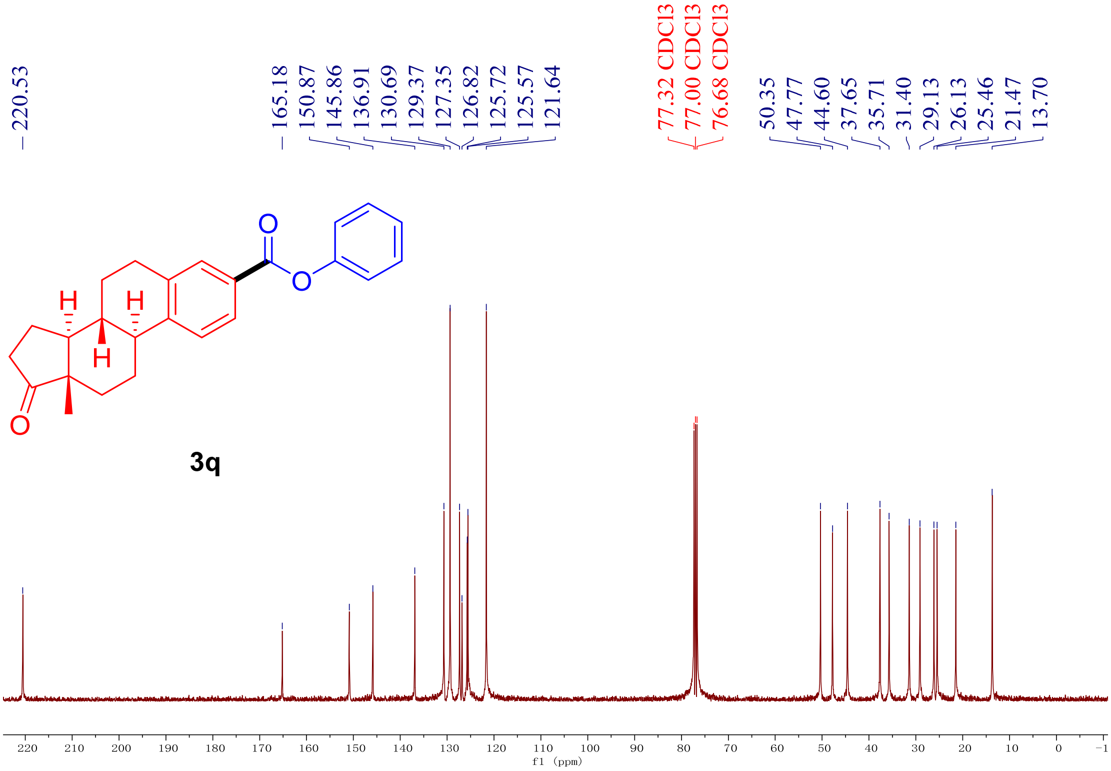

Figure S39. ^{1}H NMR spectrum of **3r** (400 MHz, $CDCl_3$)

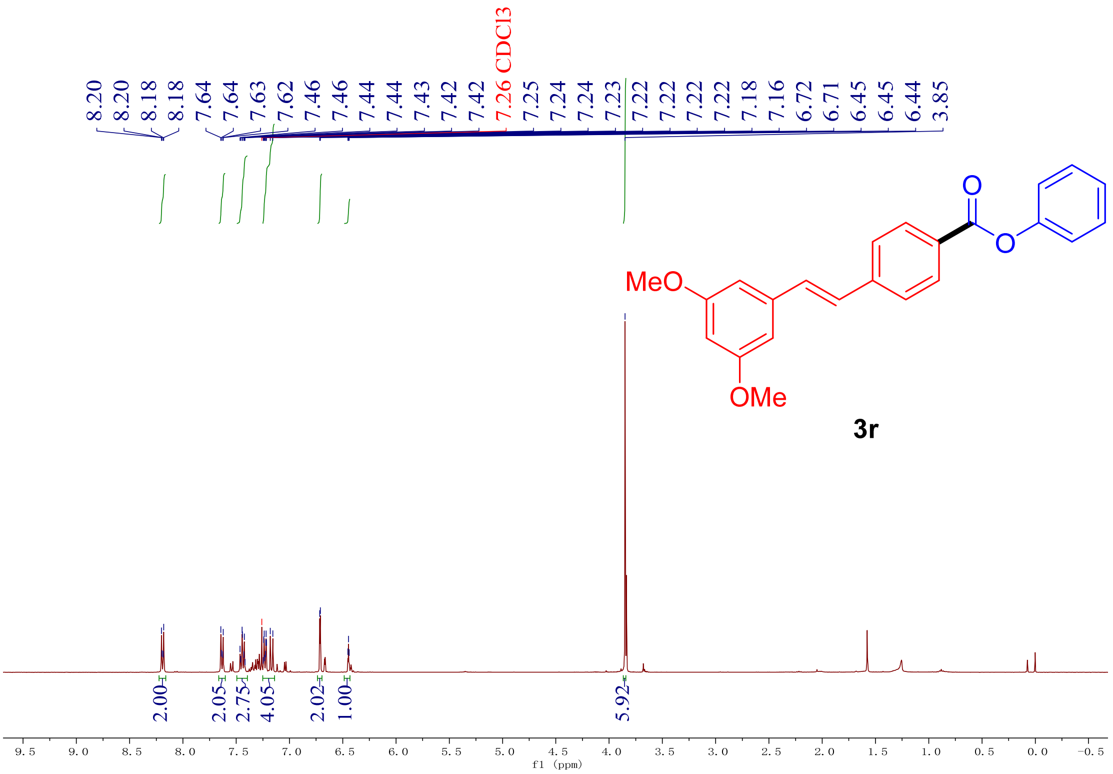

Figure S40. ^{13}C NMR spectrum of **3r** (100 MHz, $CDCl_3$)

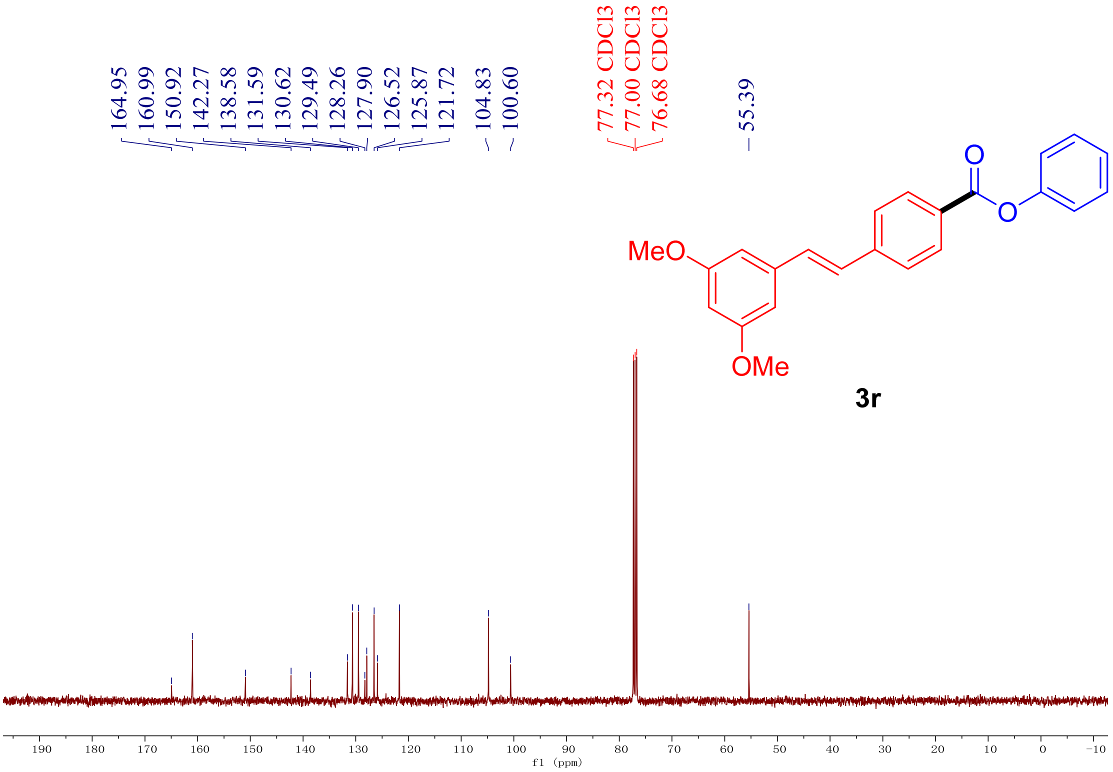

Figure S41. ^{1}H NMR spectrum of **4b** (400 MHz, $CDCl_3$)

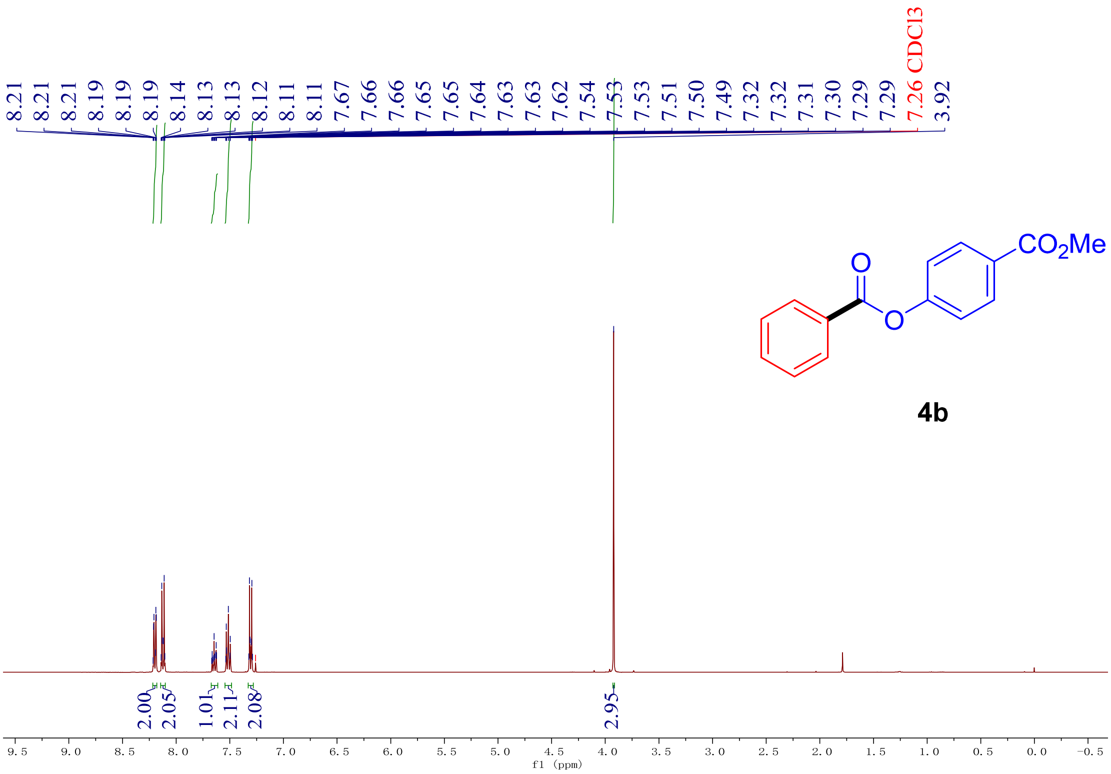

Figure S42. ^{13}C NMR spectrum of **4b** (100 MHz, $CDCl_3$)

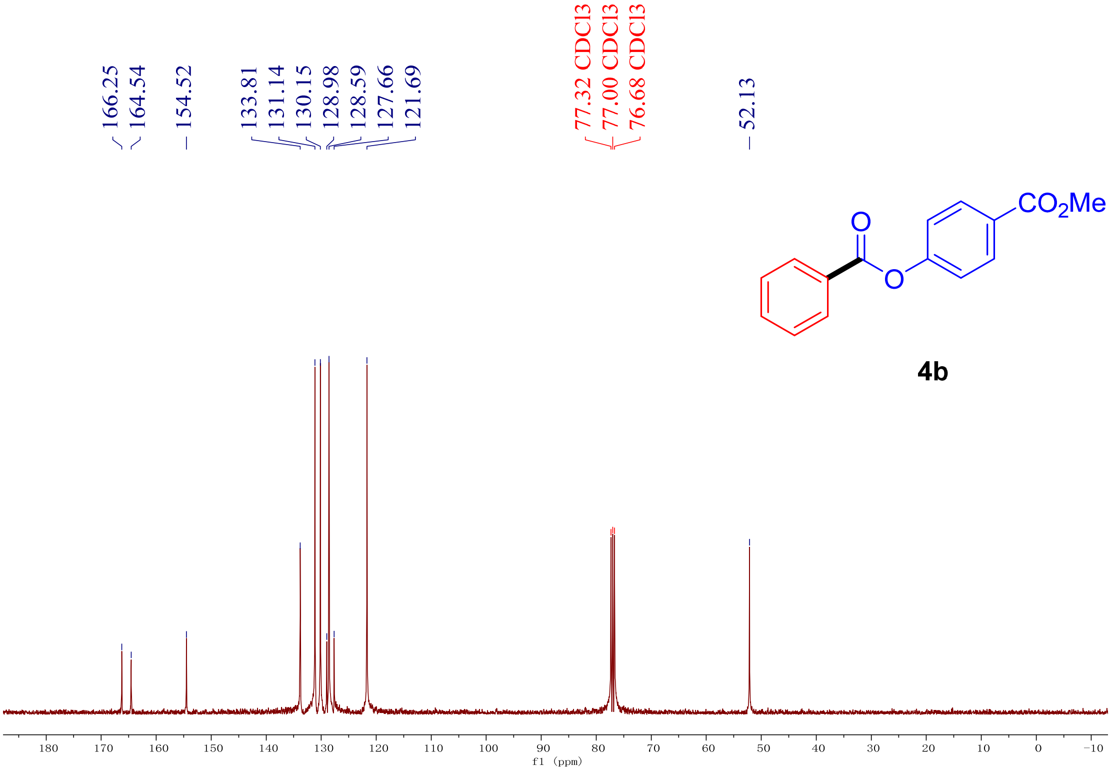

Figure S43. ^{1}H NMR spectrum of **4c** (400 MHz, $CDCl_3$)

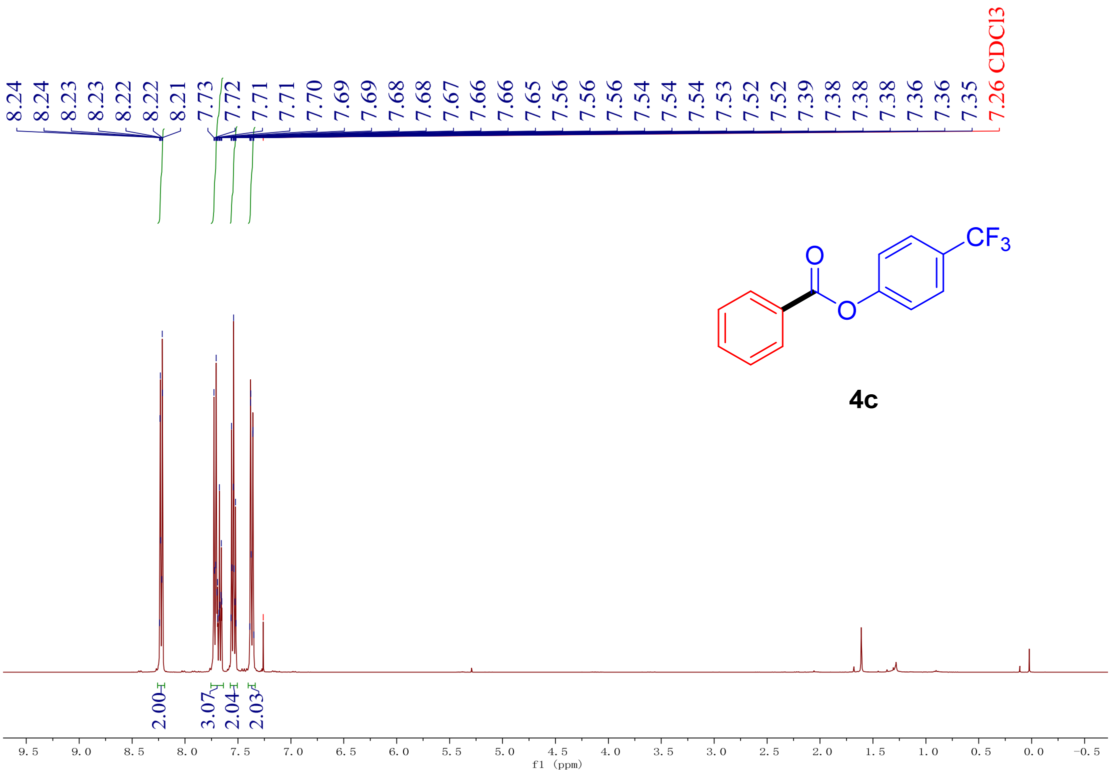

Figure S44. ^{13}C NMR spectrum of **4c** (100 MHz, $CDCl_3$)

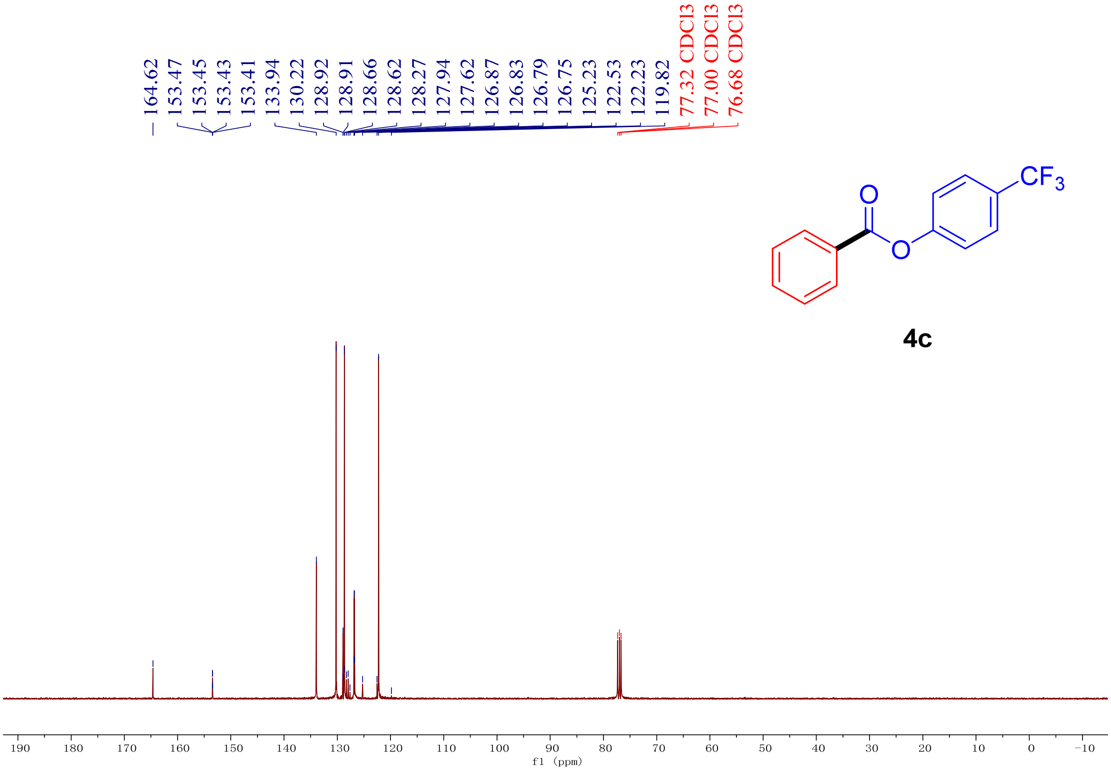

Figure S45. ^{19}F NMR spectrum of **4c** (376 MHz, $CDCl_3$)

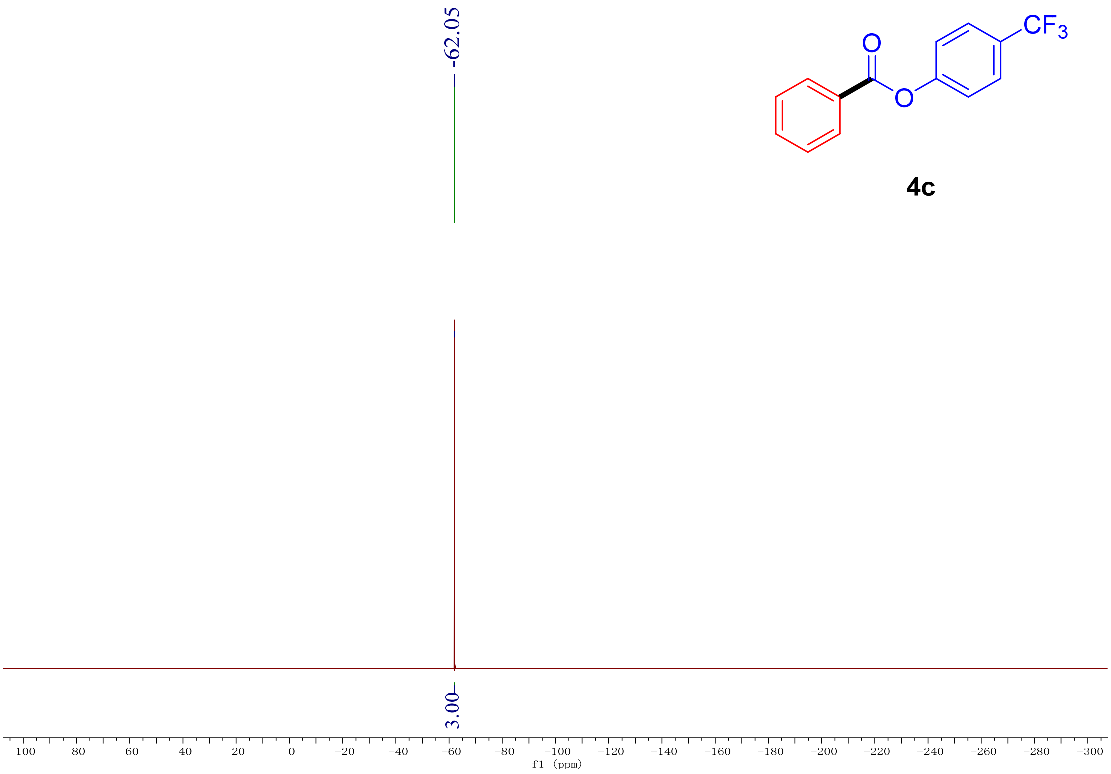

Figure S46. ^{1}H NMR spectrum of **4d** (400 MHz, $CDCl_3$)

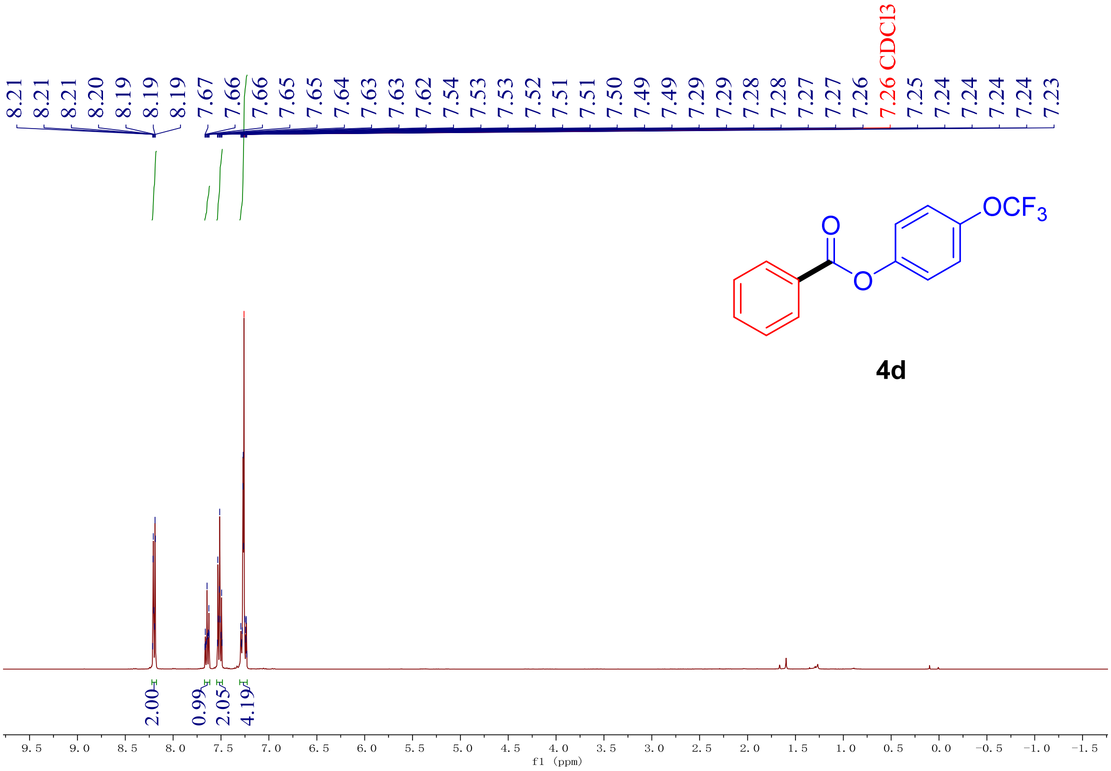

Figure S47. ^{13}C NMR spectrum of **4d** (100 MHz, $CDCl_3$)

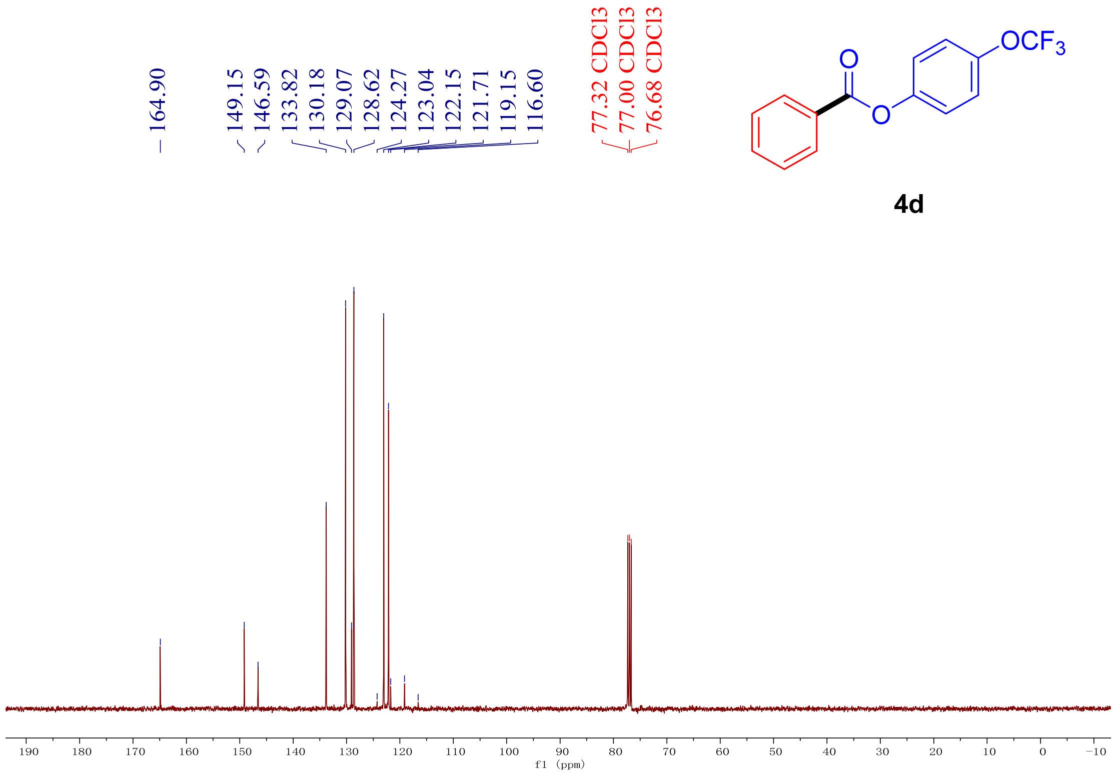

Figure S48. ^{19}F NMR spectrum of **4d** (376 MHz, $CDCl_3$)

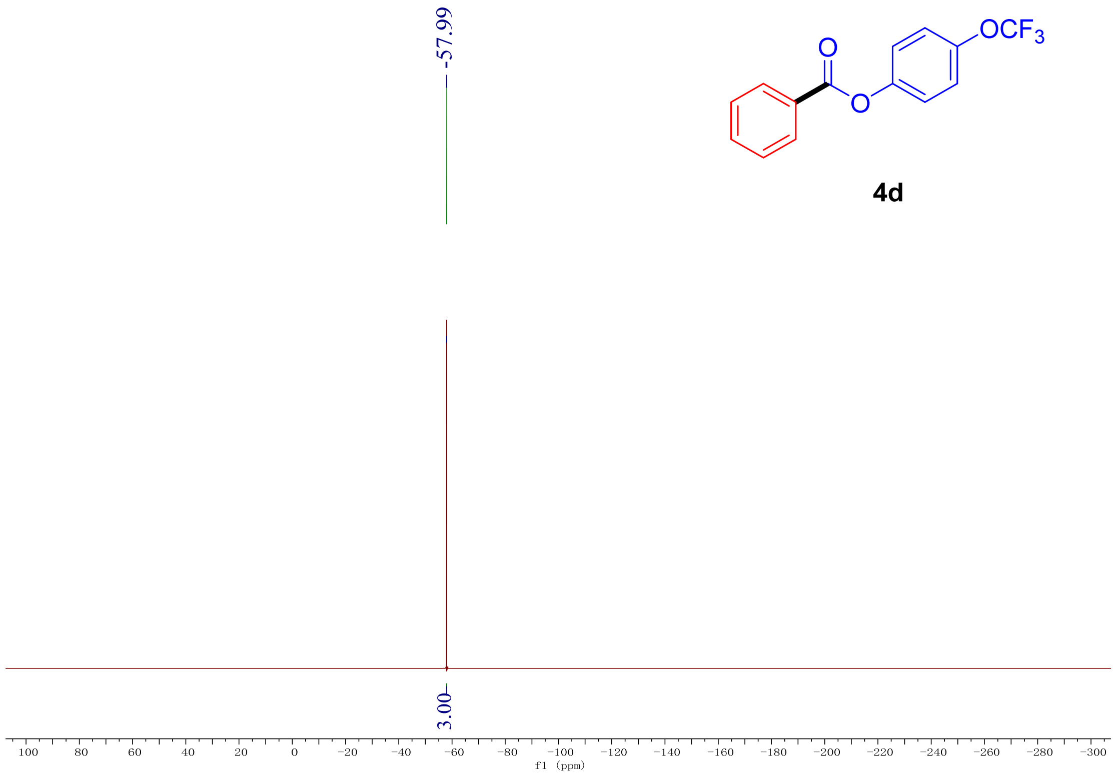

Figure S49. ^{1}H NMR spectrum of **4e** (400 MHz, $CDCl_3$)

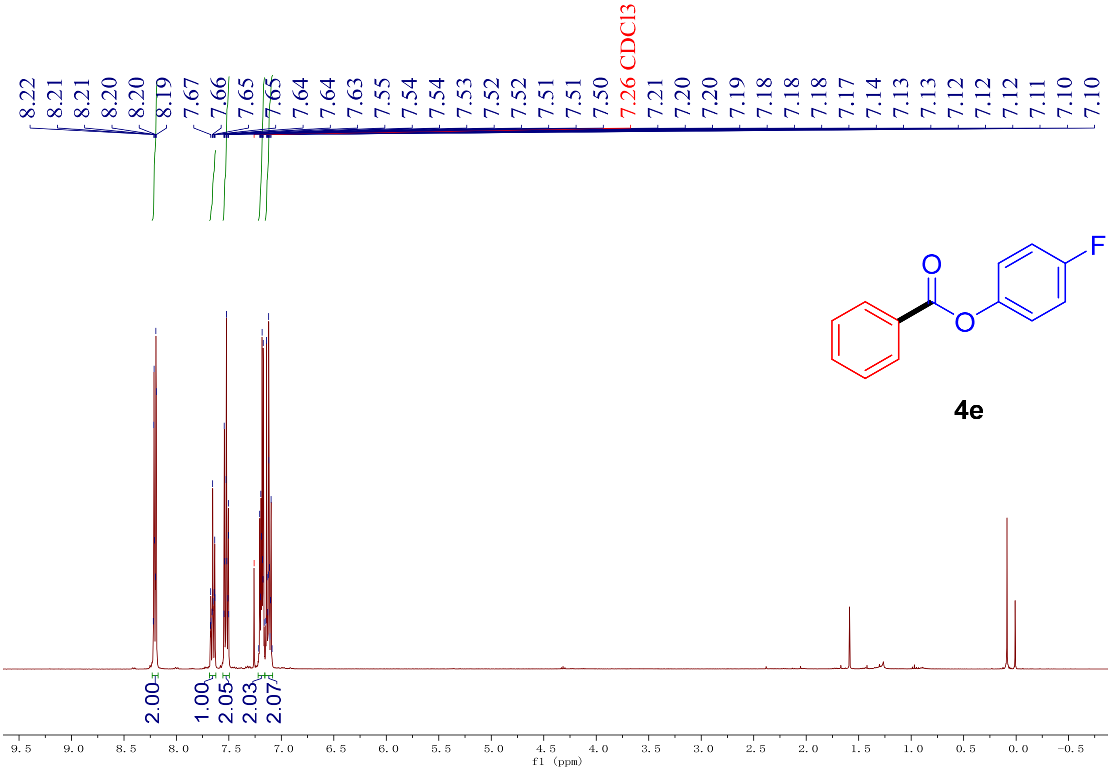

Figure S50. ^{13}C NMR spectrum of **4e** (100 MHz, $CDCl_3$)

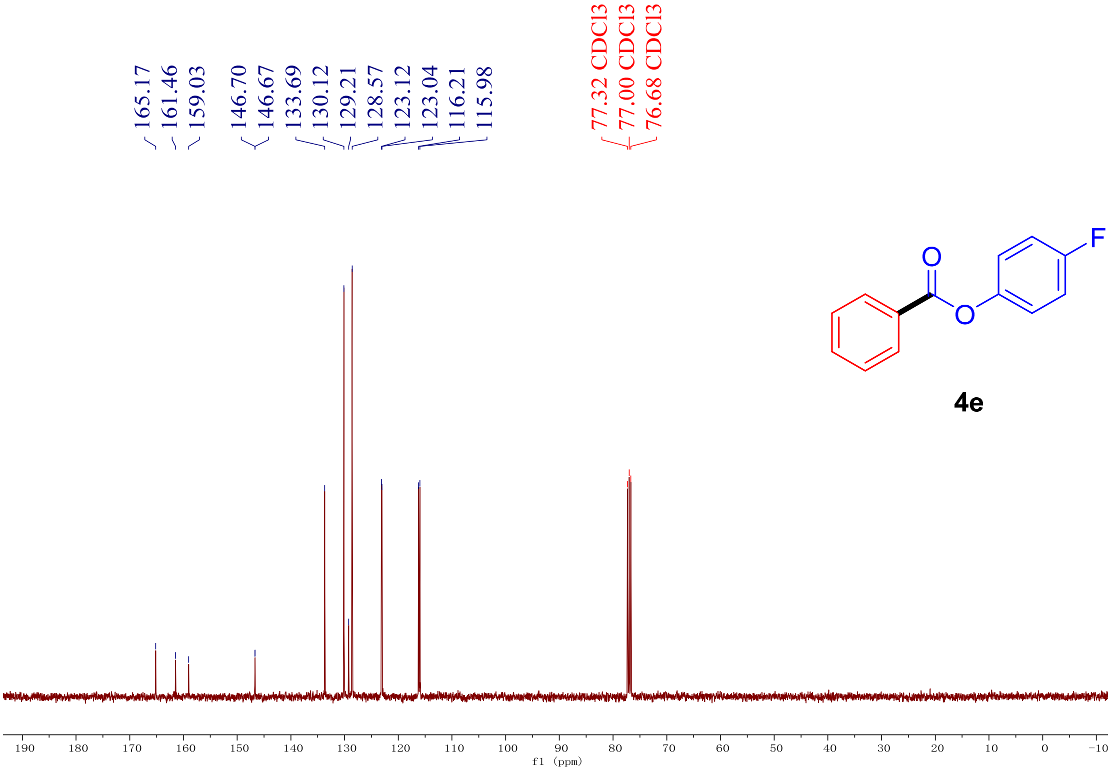

Figure S51. ^{19}F NMR spectrum of **4e** (376 MHz, $CDCl_3$)

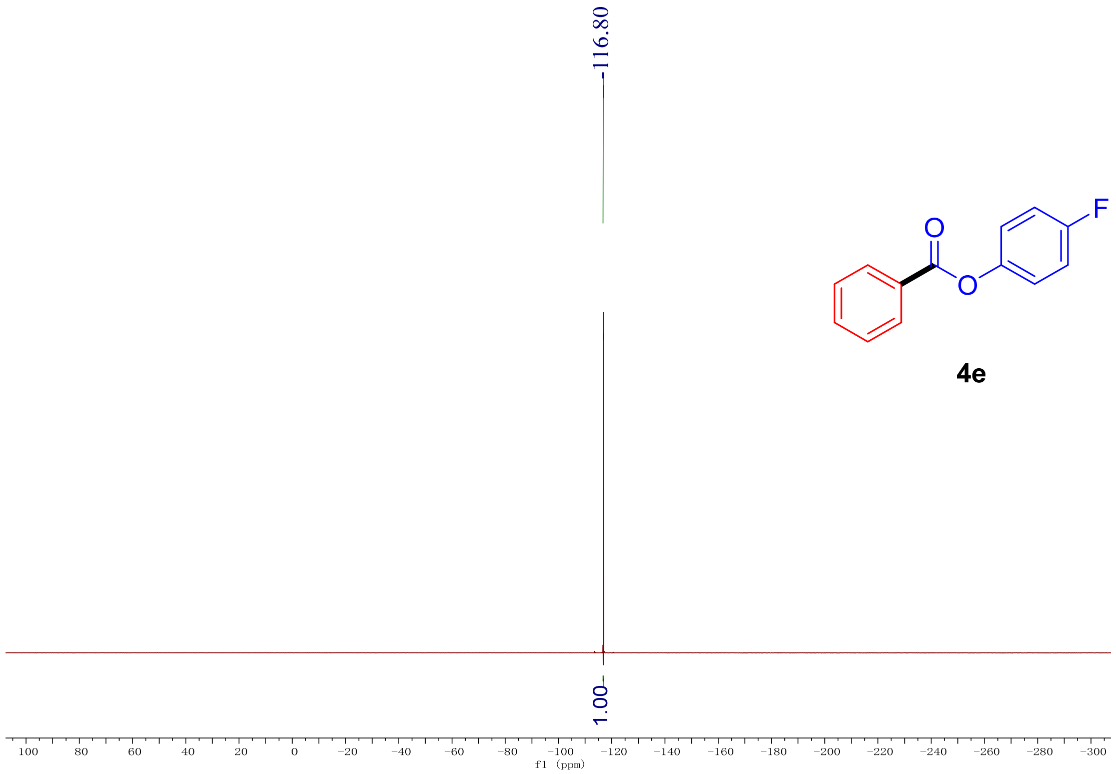

Figure S52. ^{1}H NMR spectrum of **4f** (400 MHz, $CDCl_3$)

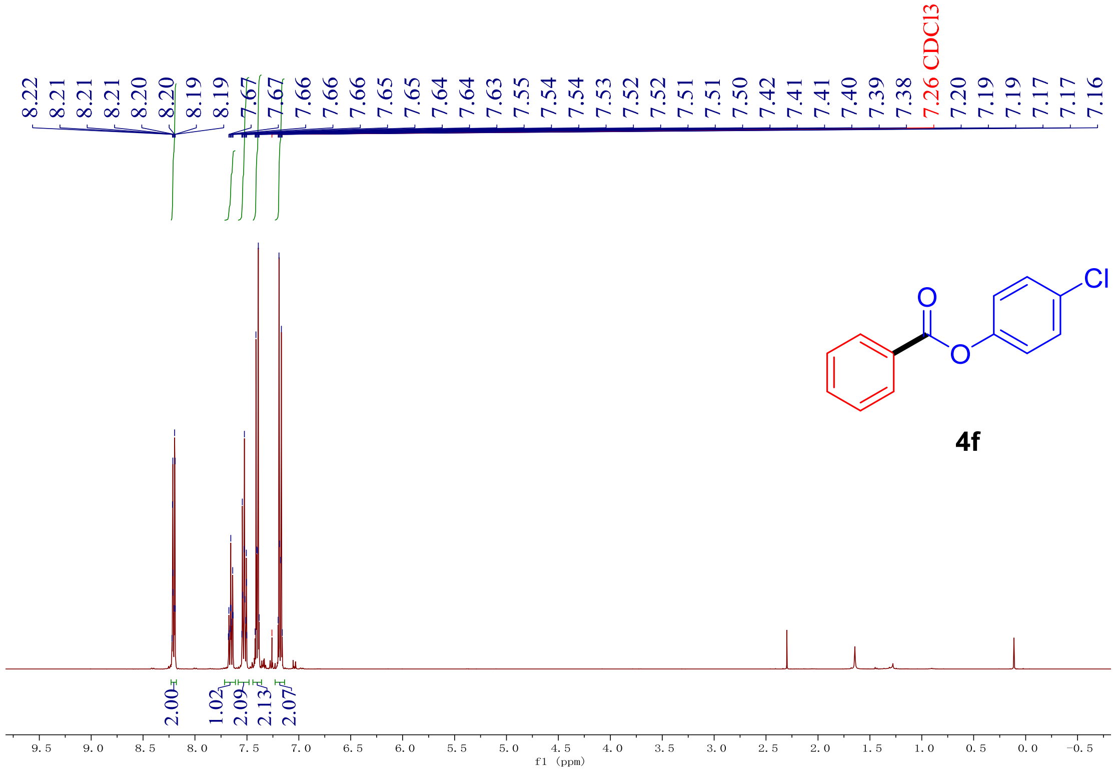

Figure S53. ^{13}C NMR spectrum of **4f** (100 MHz, $CDCl_3$)

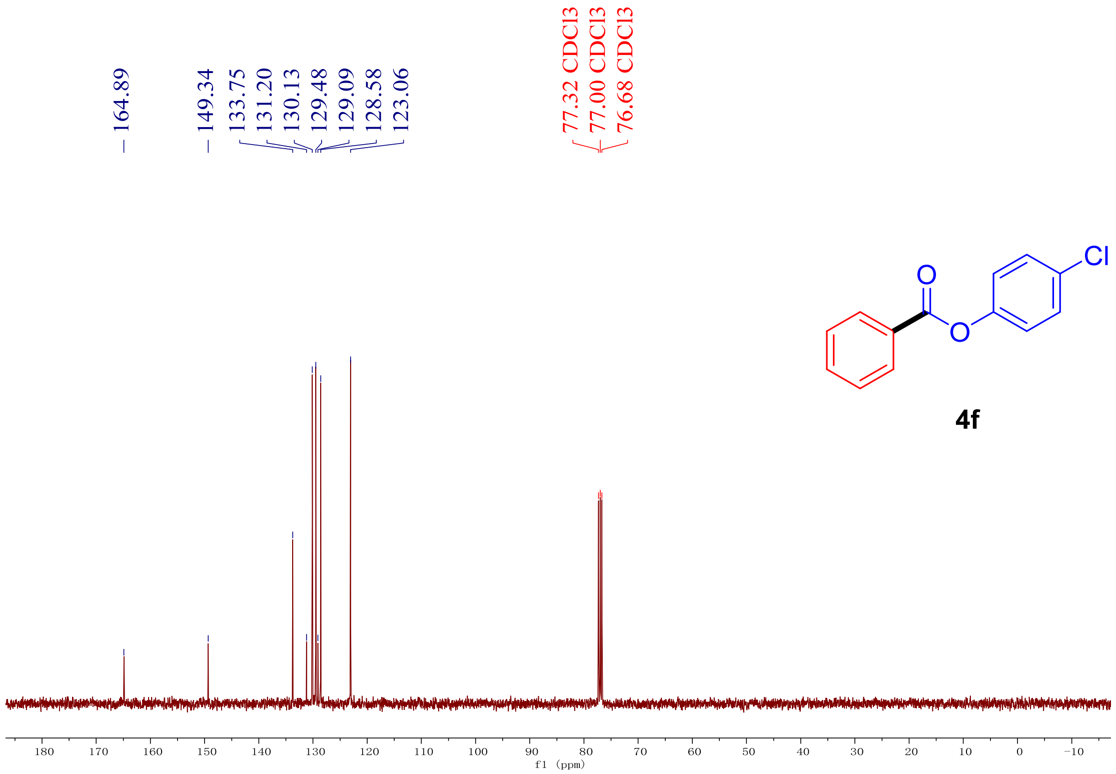

Figure S54. ^{1}H NMR spectrum of **4g** (400 MHz, $CDCl_3$)

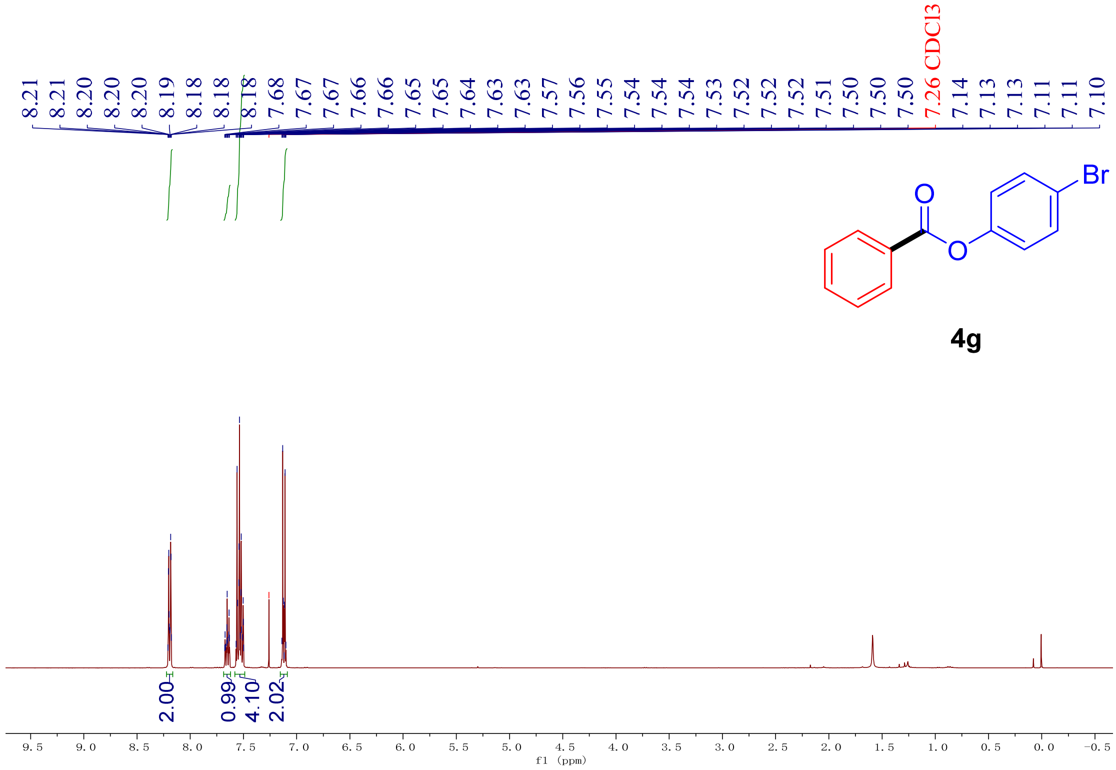

Figure S55. ^{13}C NMR spectrum of **4g** (100 MHz, $CDCl_3$)

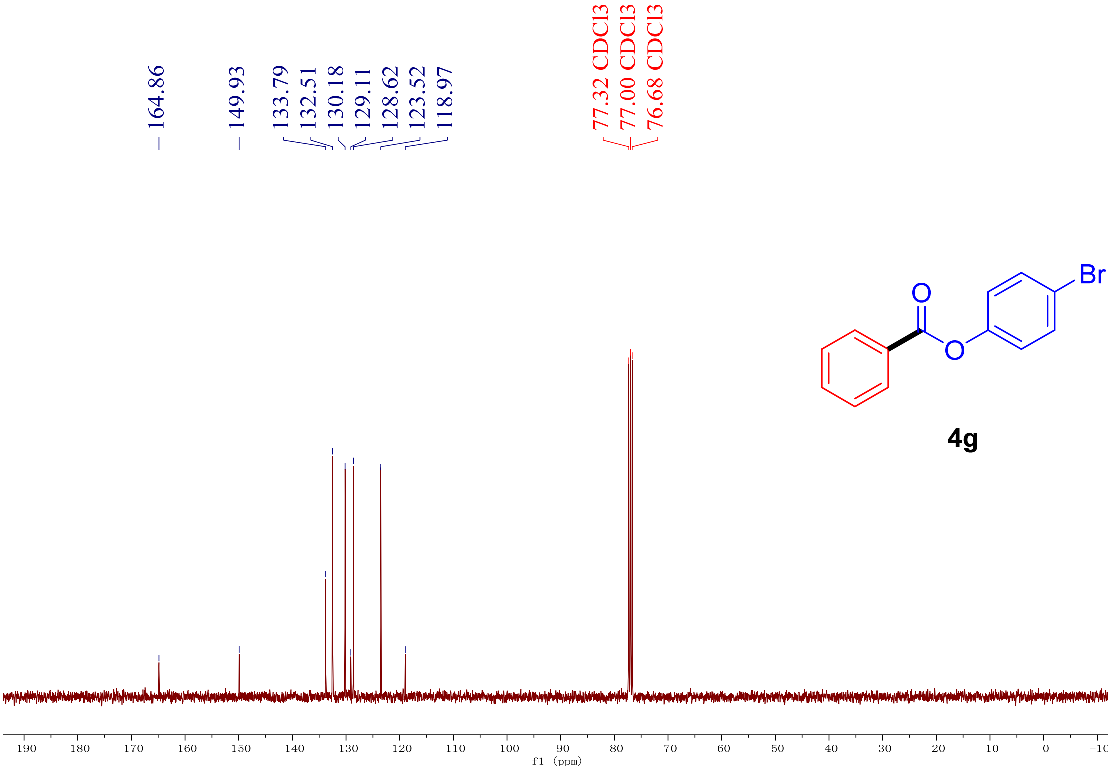

Figure S56. ^{1}H NMR spectrum of **4h** (400 MHz, $CDCl_3$)

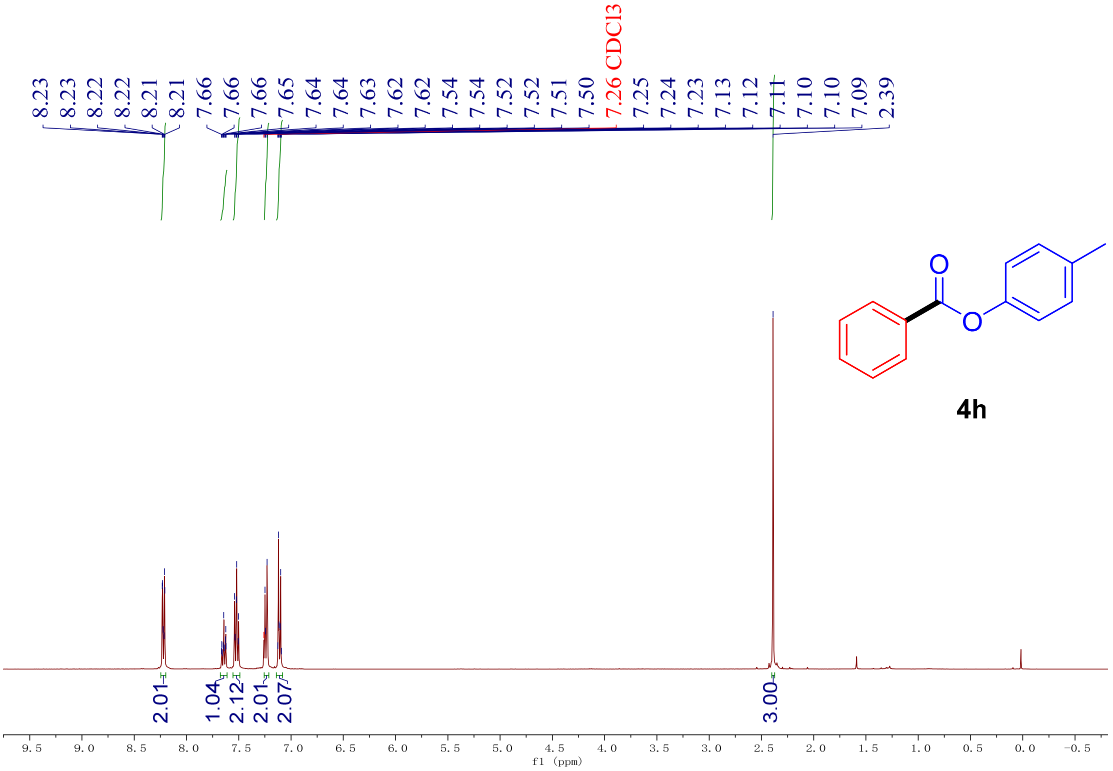

Figure S57. ^{13}C NMR spectrum of **4h** (100 MHz, $CDCl_3$)

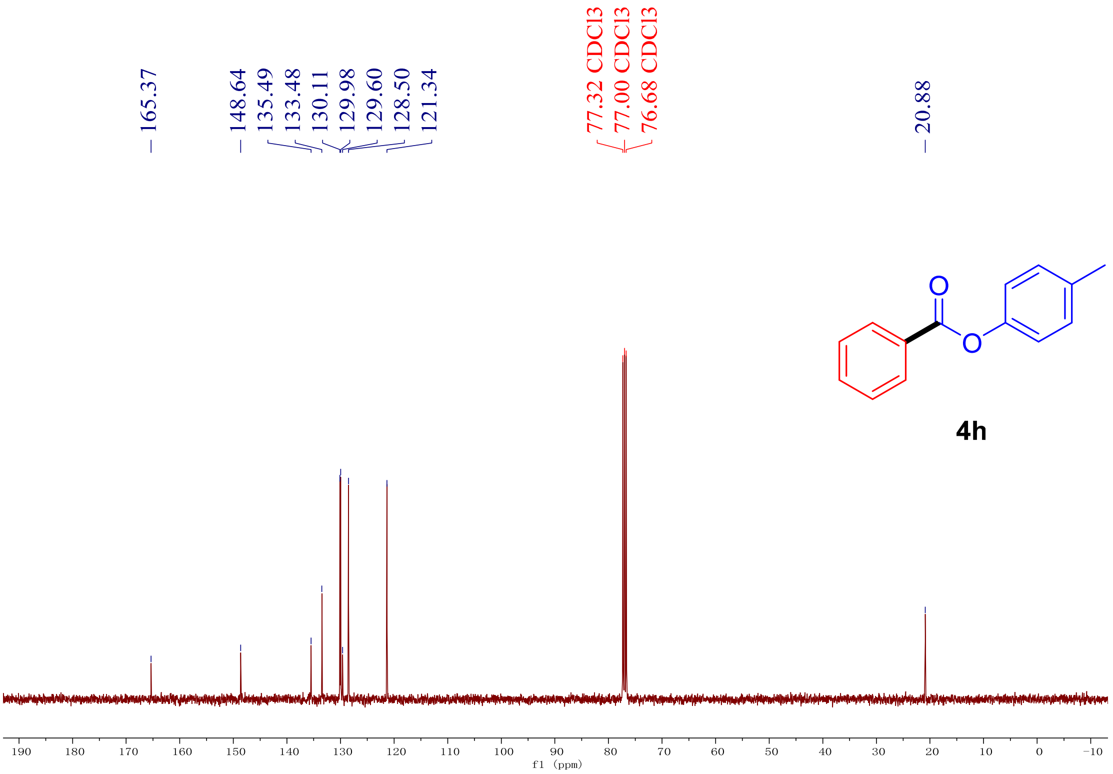

Figure S58. ^{1}H NMR spectrum of **4i** (400 MHz, $CDCl_3$)

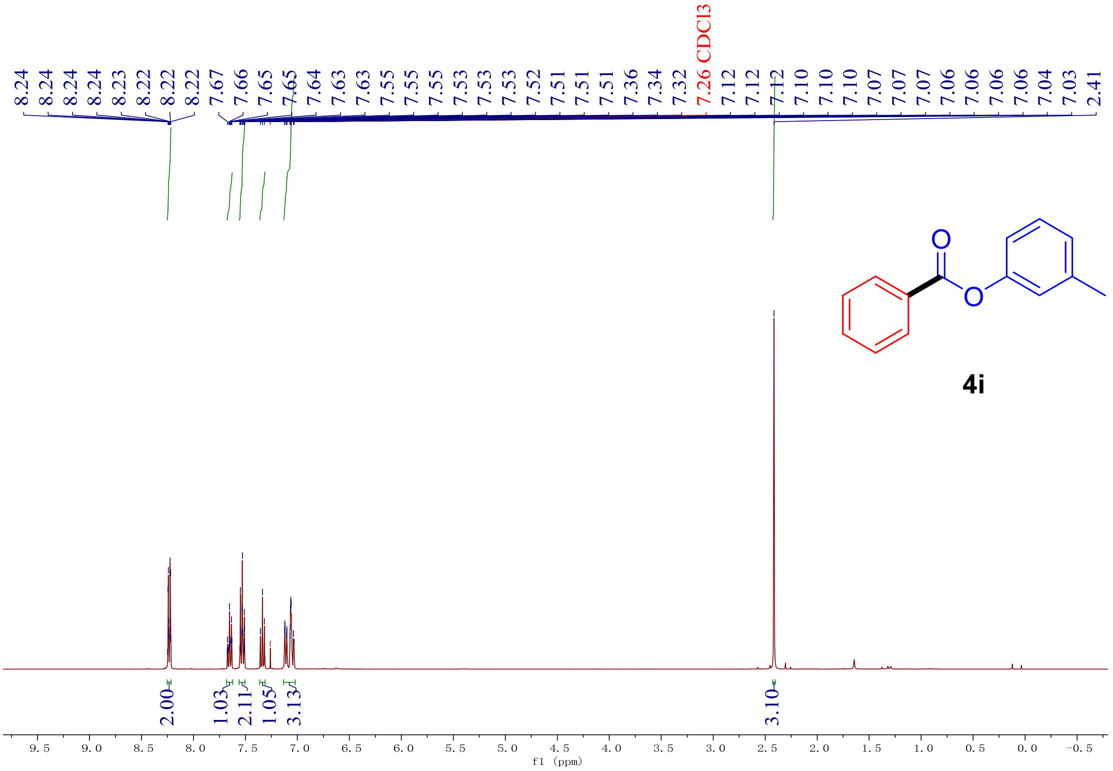

Figure S59. ^{13}C NMR spectrum of **4i** (100 MHz, $CDCl_3$)

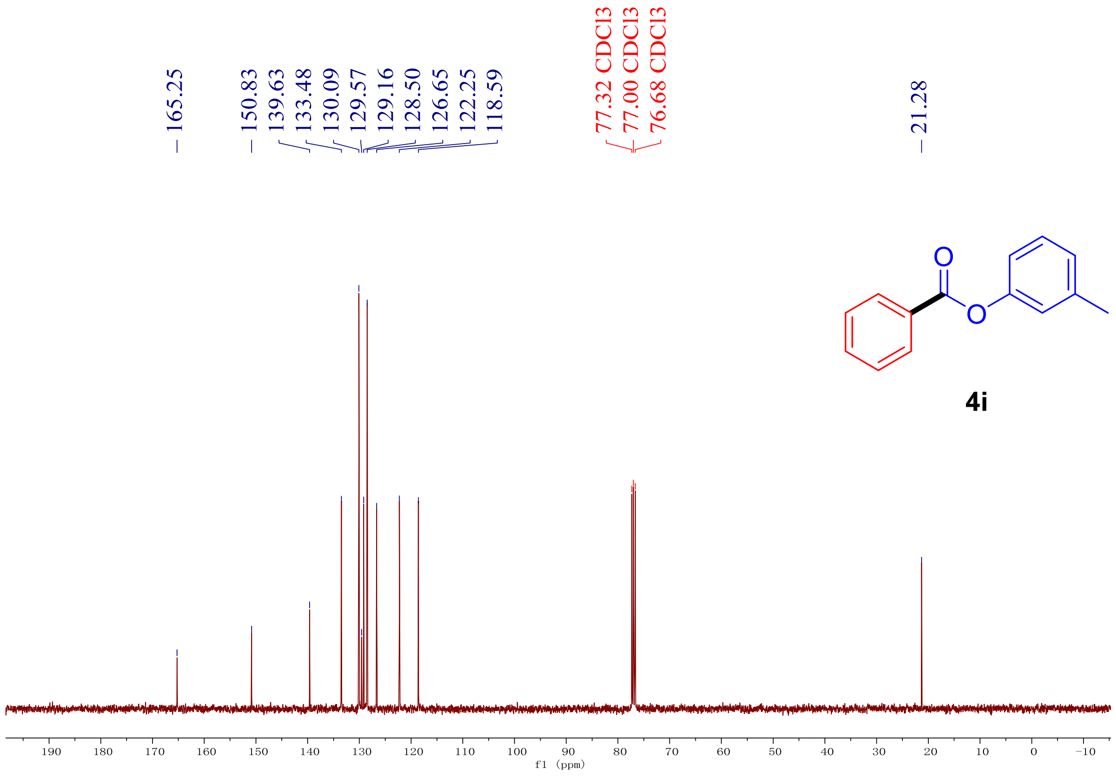

Figure S60. ^{1}H NMR spectrum of **4j** (400 MHz, $CDCl_3$)

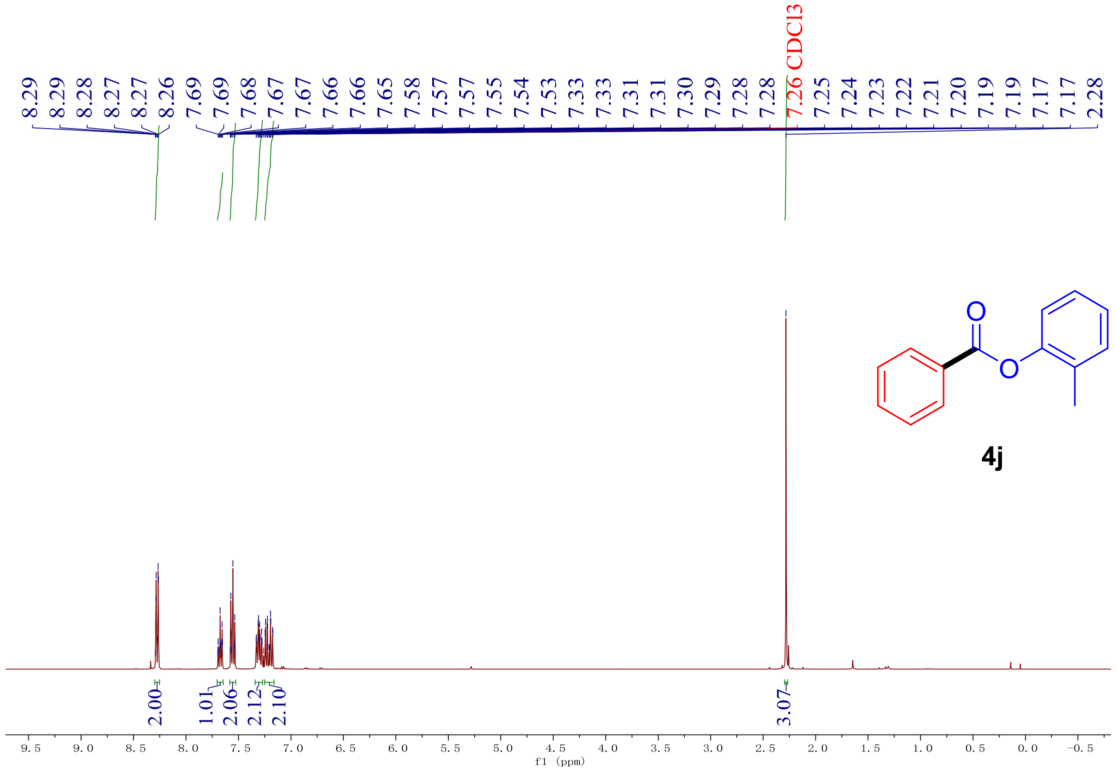

Figure S61. ^{13}C NMR spectrum of **4j** (100 MHz, $CDCl_3$)

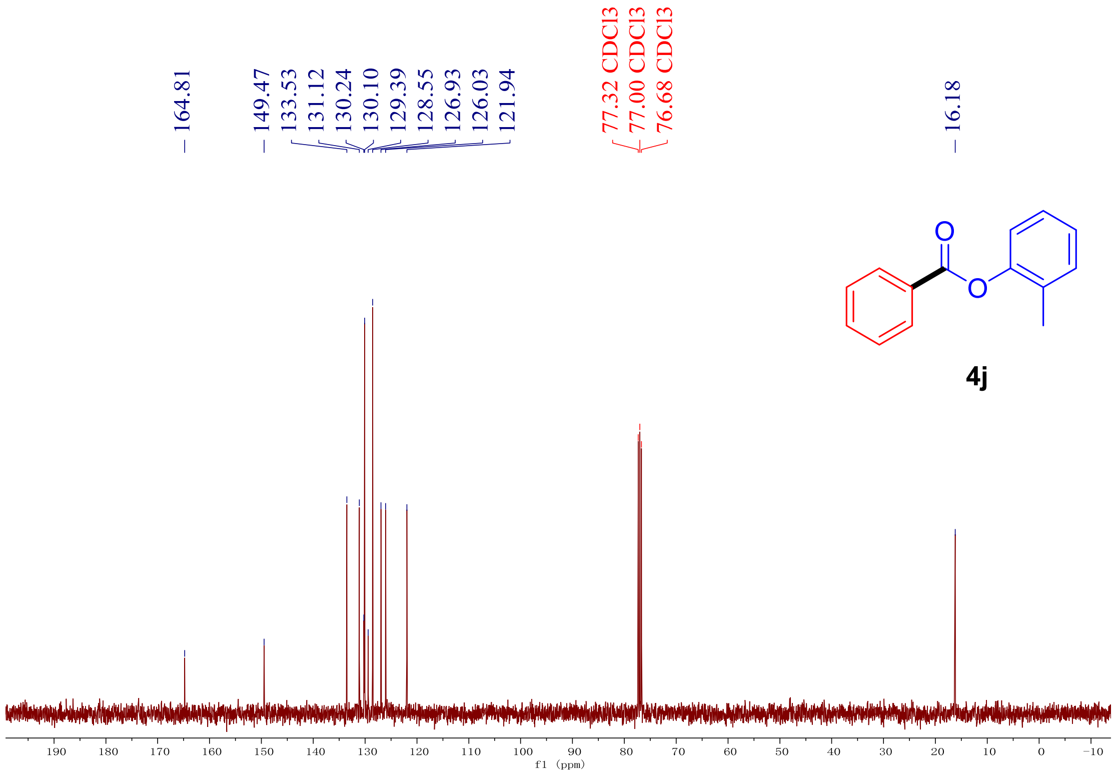

Figure S62. ^{1}H NMR spectrum of **4k** (400 MHz, $CDCl_3$)

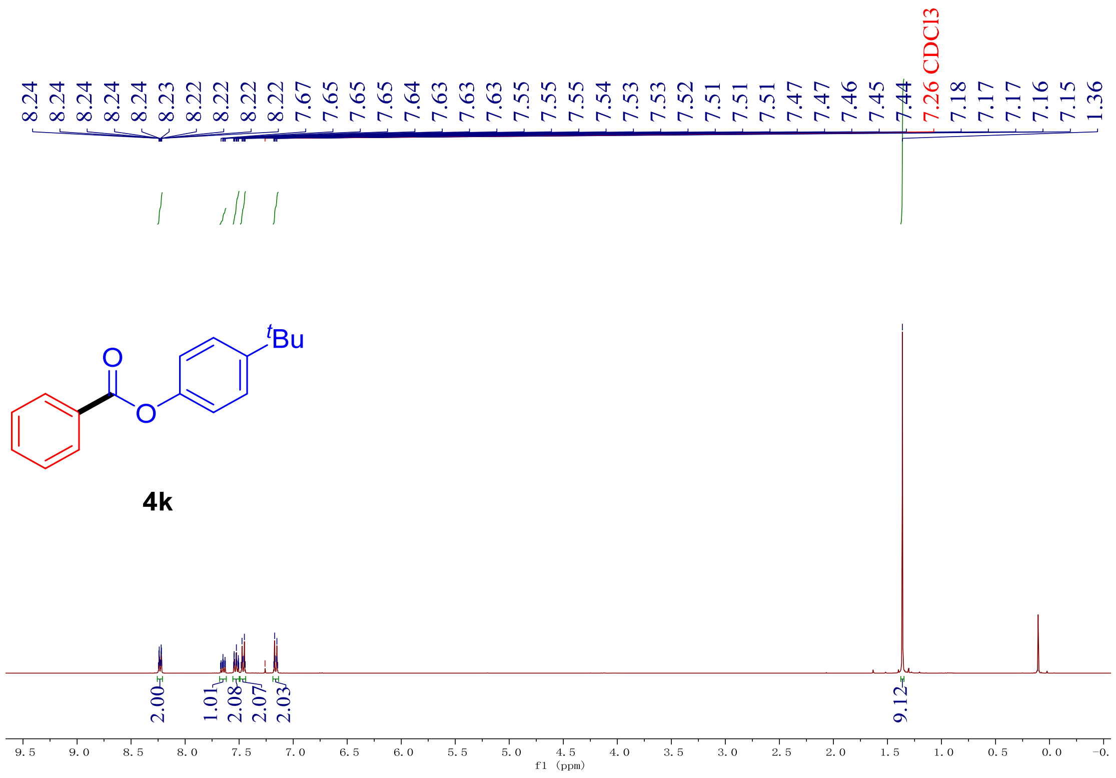

Figure S63. ^{13}C NMR spectrum of **4k** (100 MHz, $CDCl_3$)

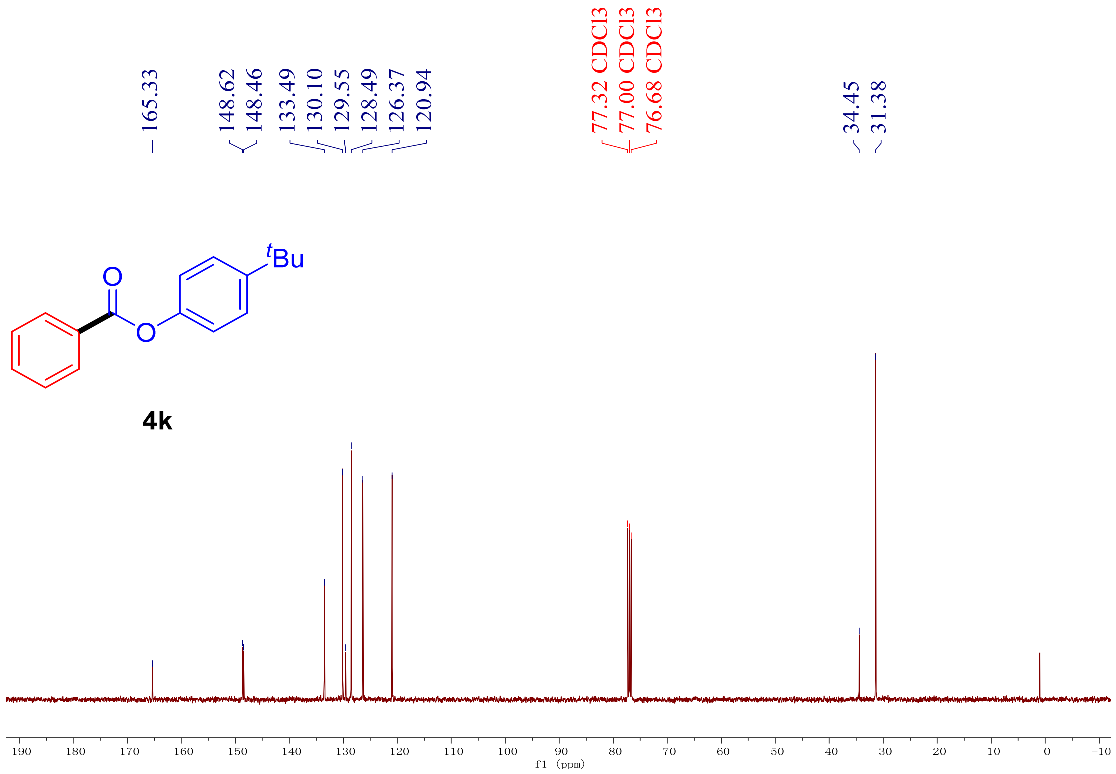

Figure S64. ^{1}H NMR spectrum of **4l** (400 MHz, $CDCl_3$)

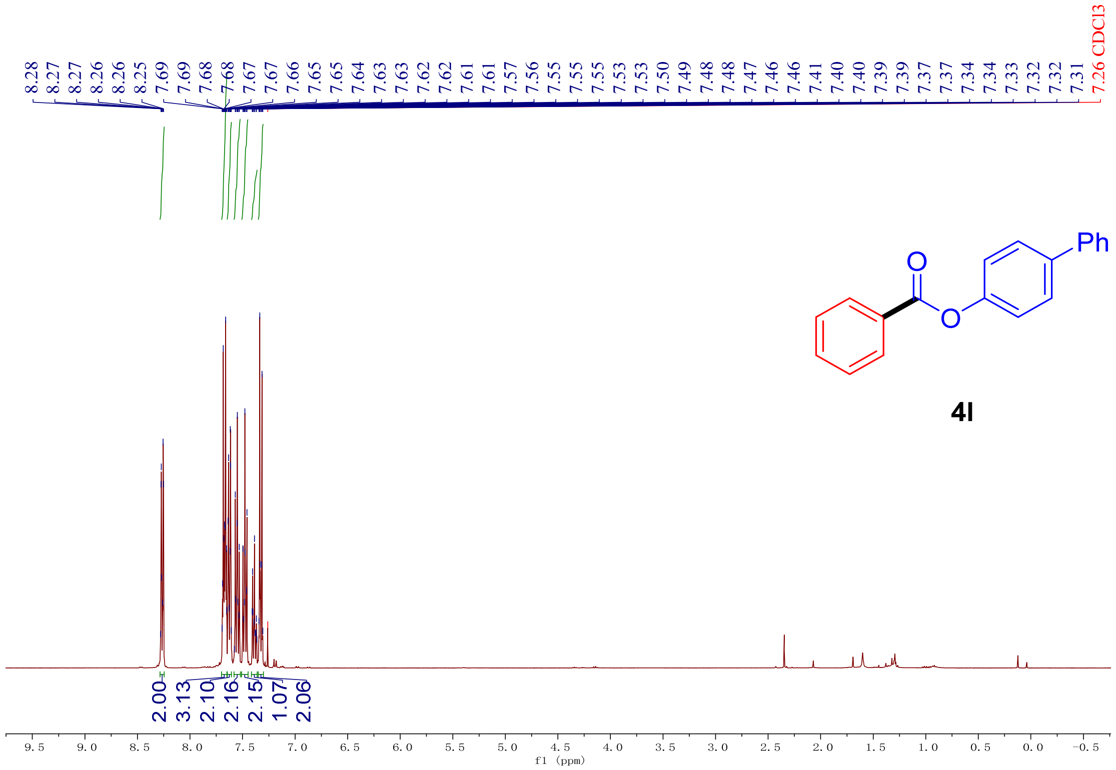

Figure S65. ^{13}C NMR spectrum of **4l** (100 MHz, $CDCl_3$)

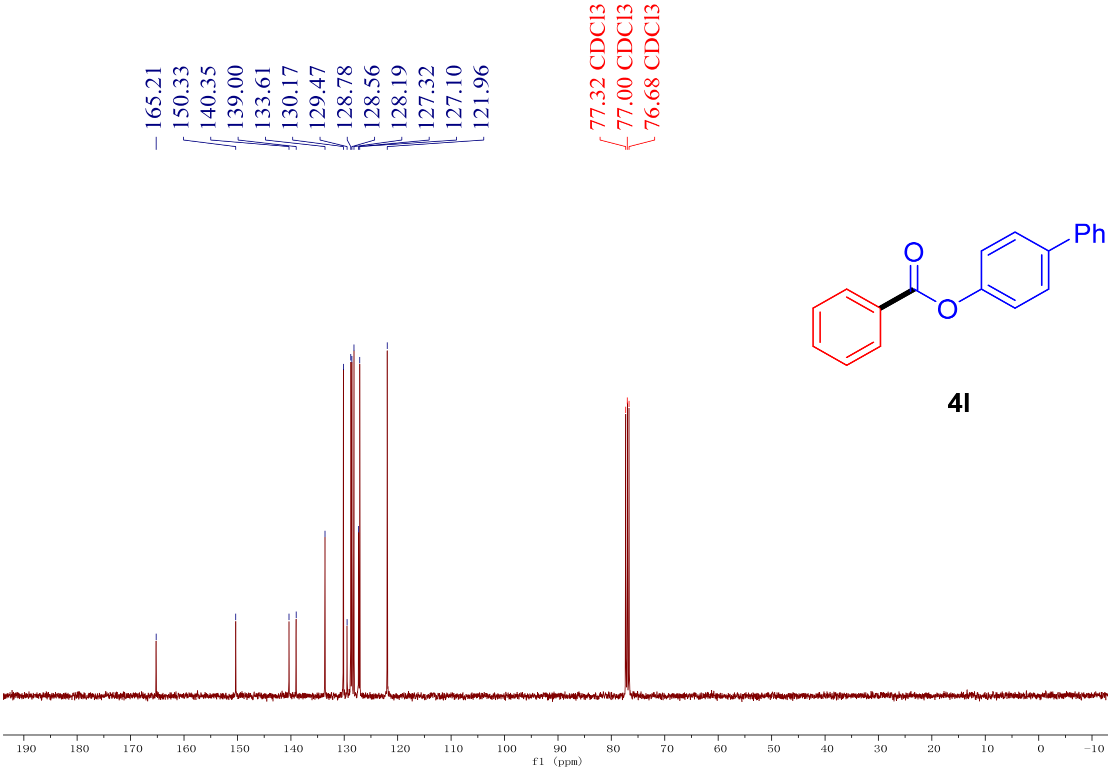

Figure S66. ^{1}H NMR spectrum of **4m** (400 MHz, $CDCl_3$)

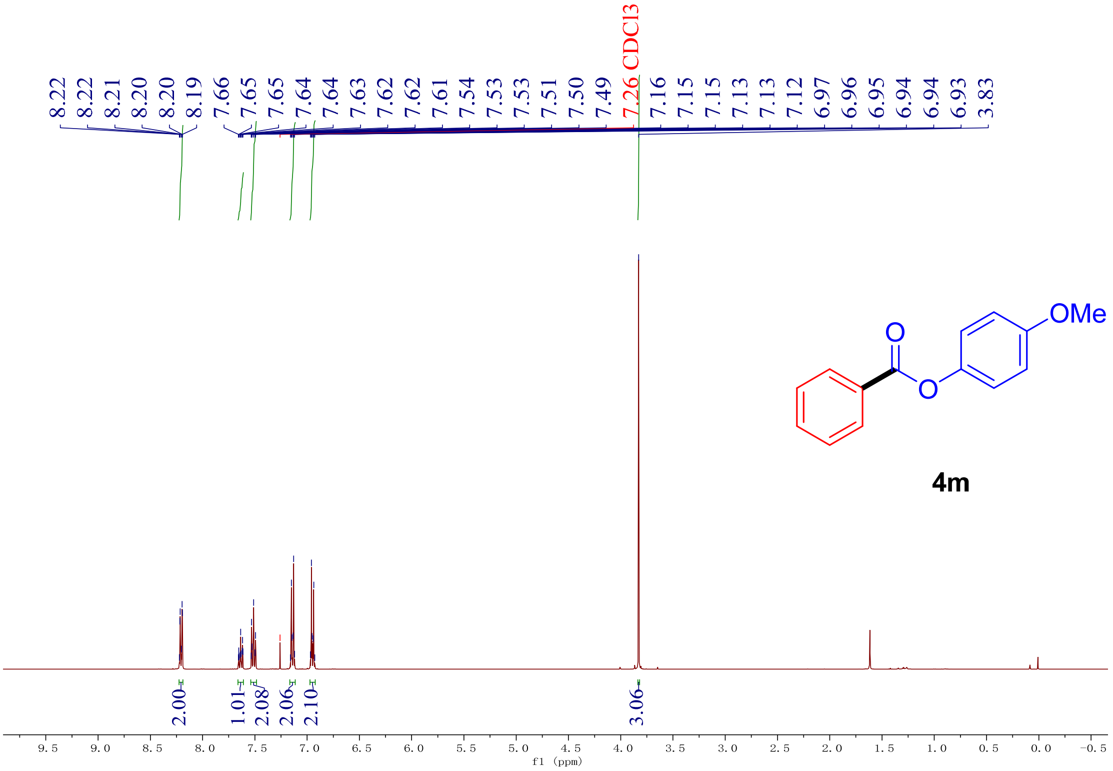

Figure S67. ^{13}C NMR spectrum of **4m** (100 MHz, $CDCl_3$)

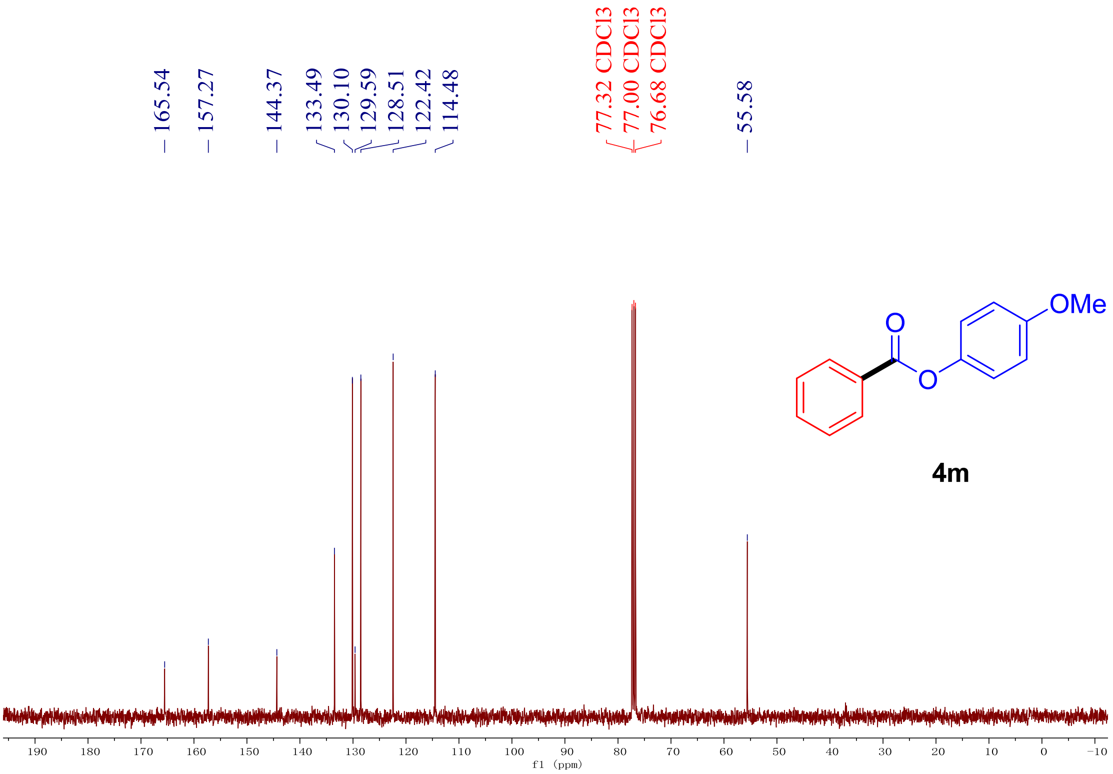

Figure S68. ^{1}H NMR spectrum of **4n** (400 MHz, $CDCl_3$)

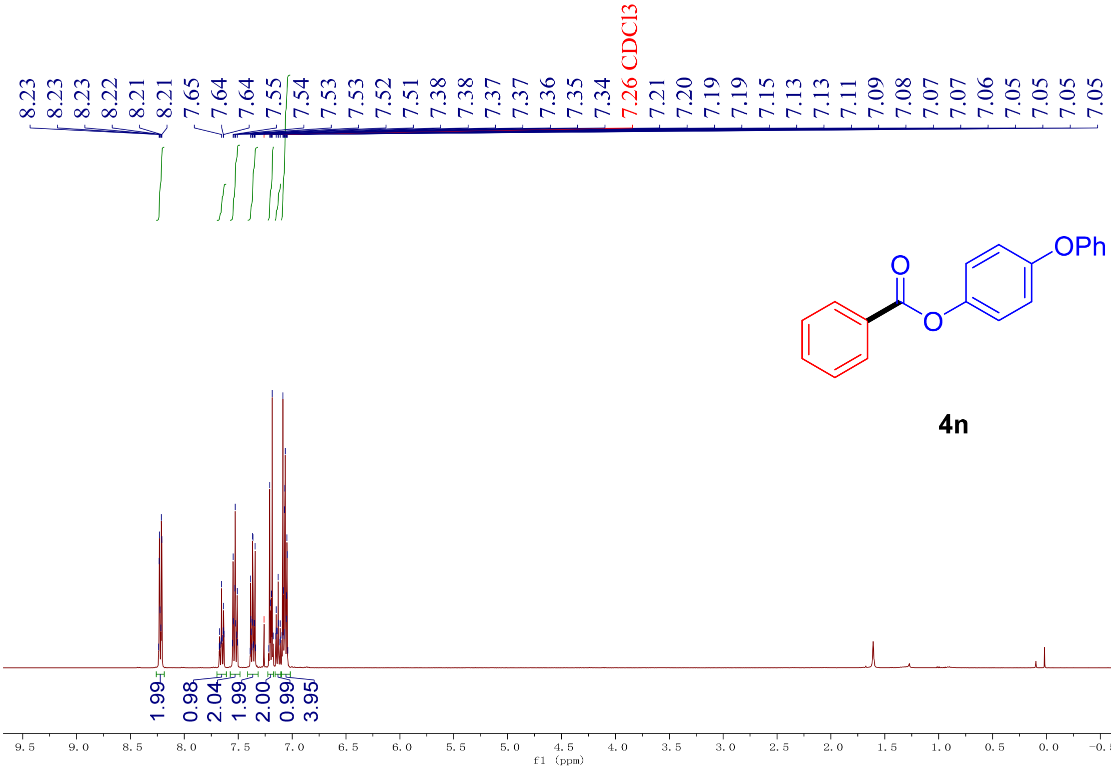

Figure S69. ^{13}C NMR spectrum of **4n** (100 MHz, $CDCl_3$)

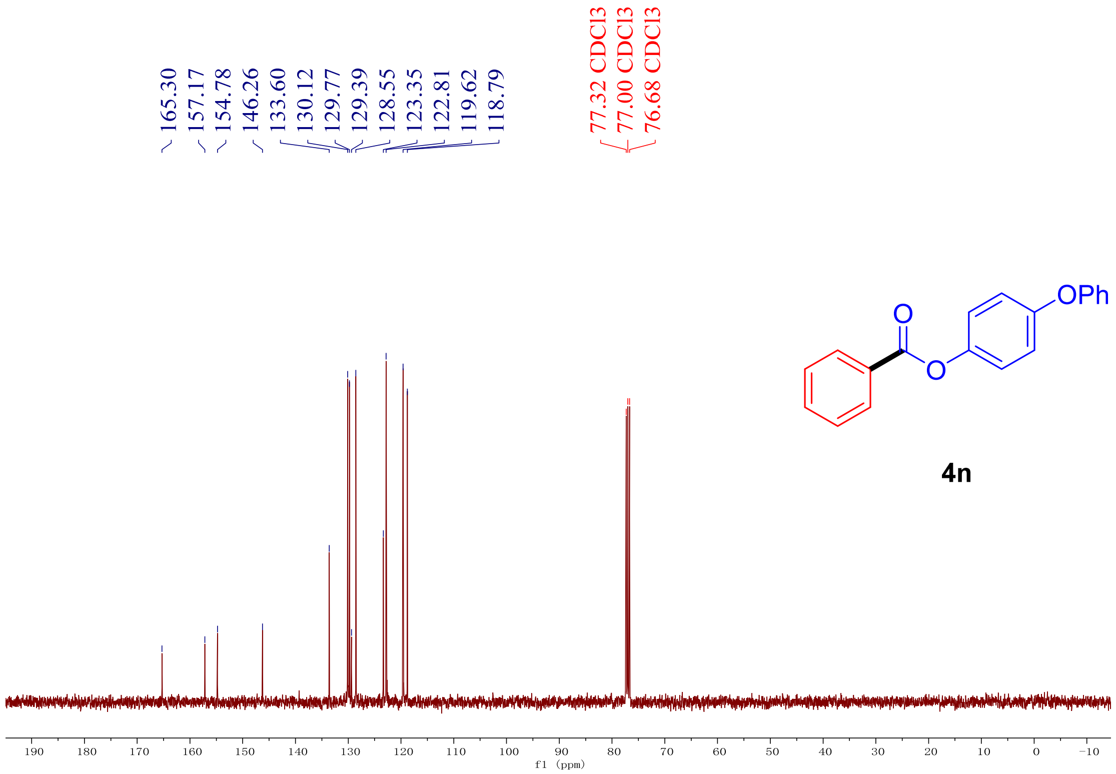

Figure S70. ^{1}H NMR spectrum of **4o** (400 MHz, $CDCl_3$)

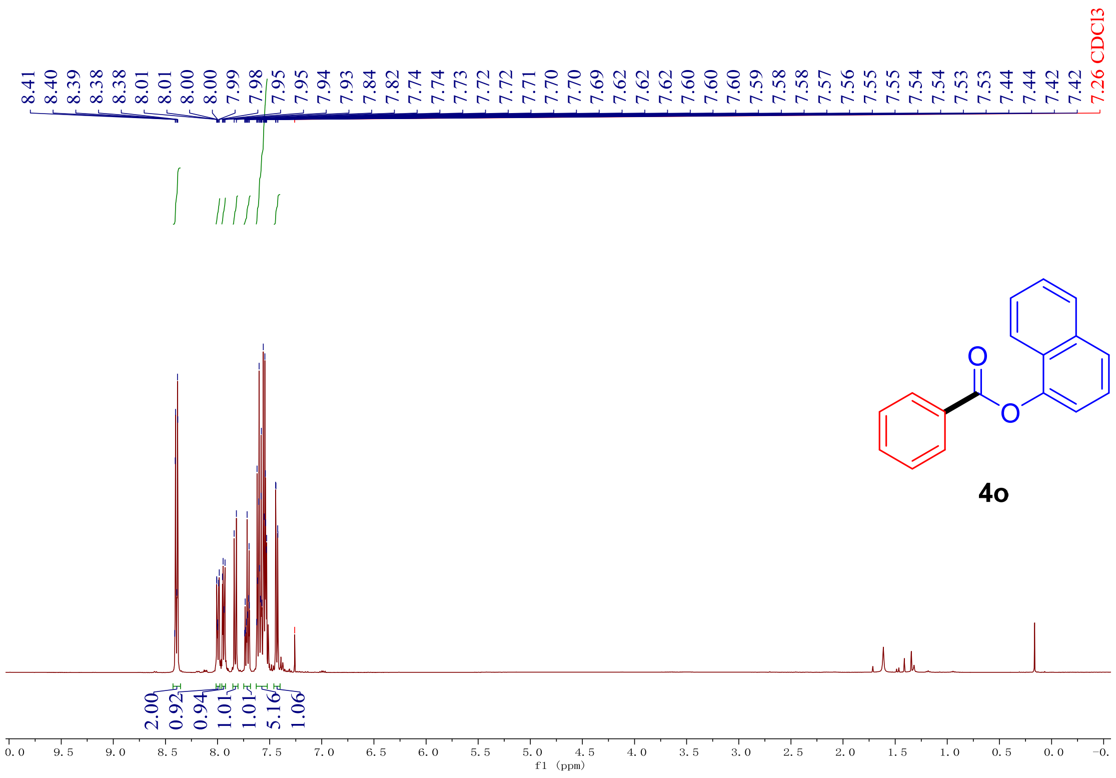

Figure S71. ^{13}C NMR spectrum of **4o** (100 MHz, $CDCl_3$)

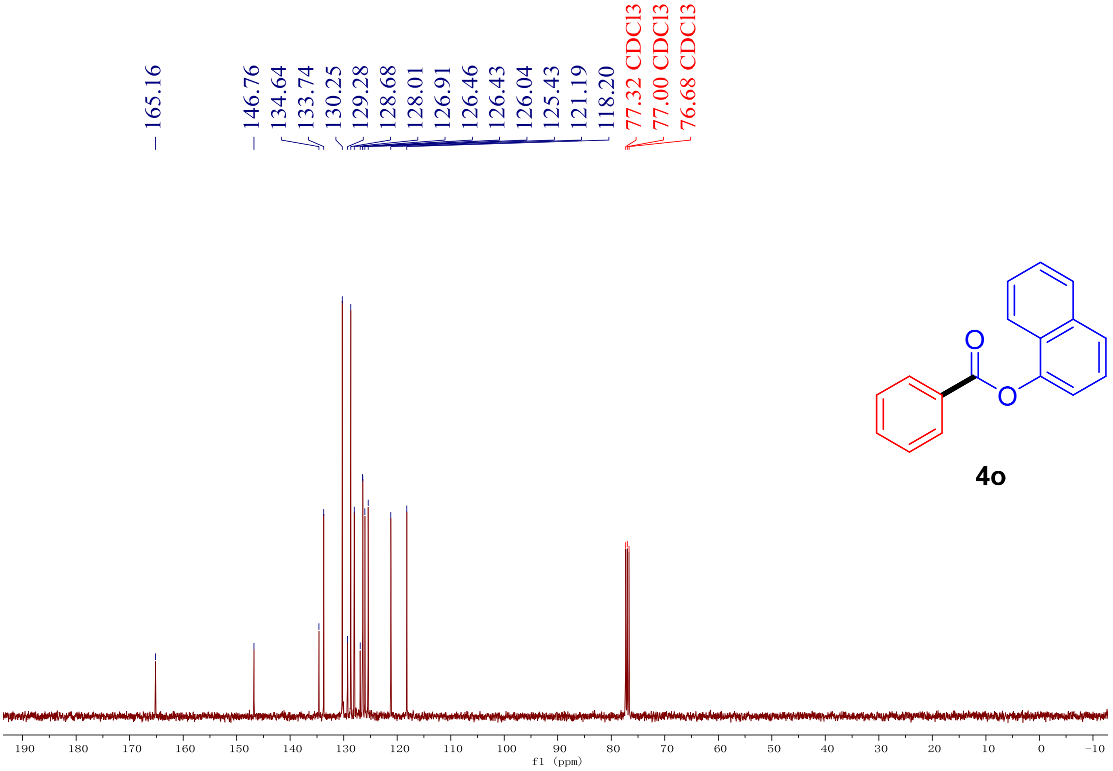

Figure S72. ^{1}H NMR spectrum of **4p** (400 MHz, $CDCl_3$)

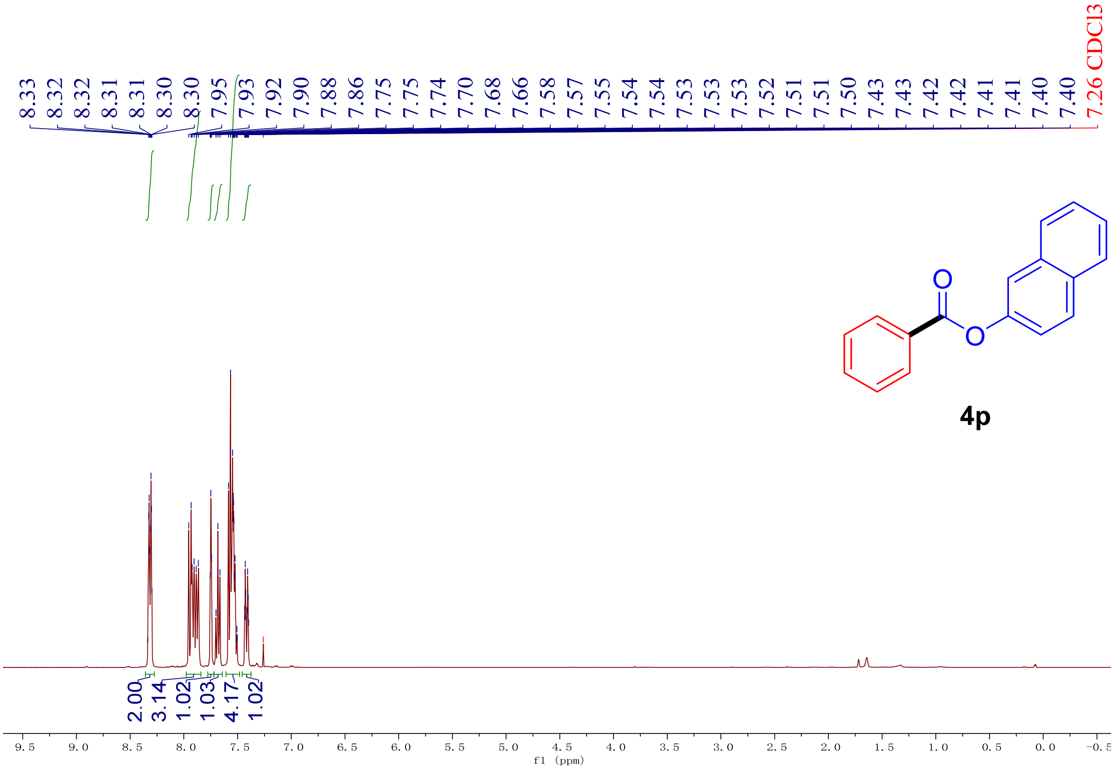

Figure S73. ^{13}C NMR spectrum of **4p** (100 MHz, $CDCl_3$)

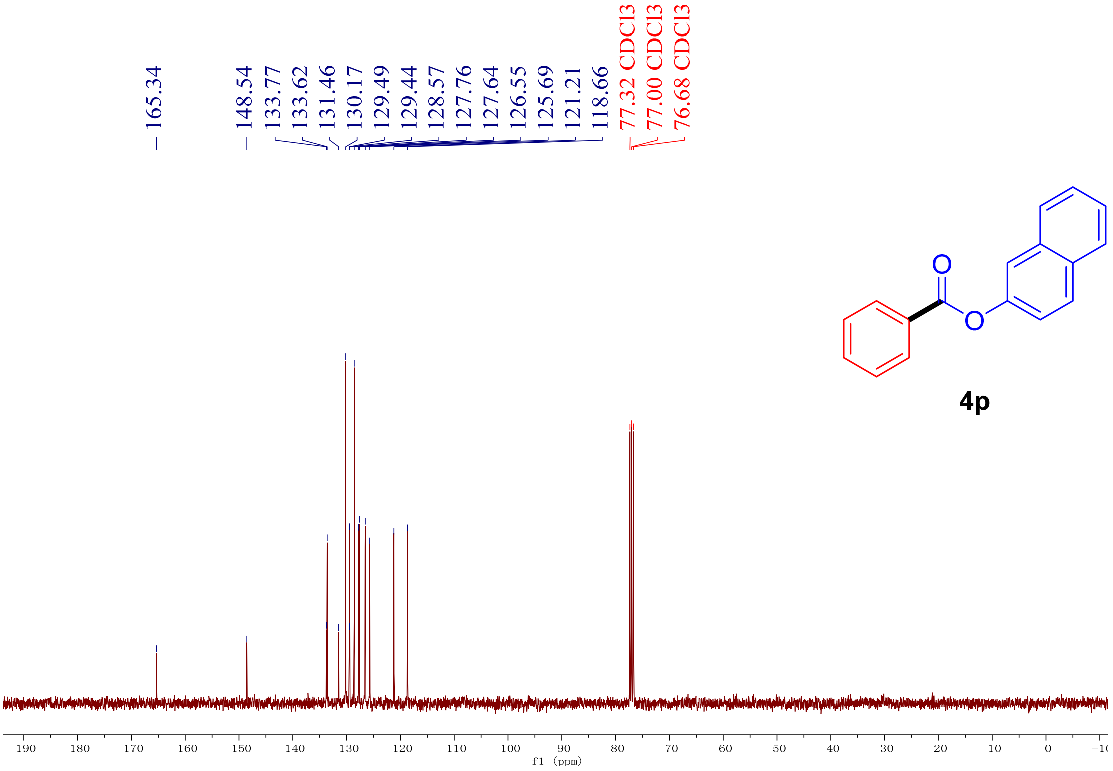

Figure S74. ^{1}H NMR spectrum of **4q** (400 MHz, $CDCl_3$)

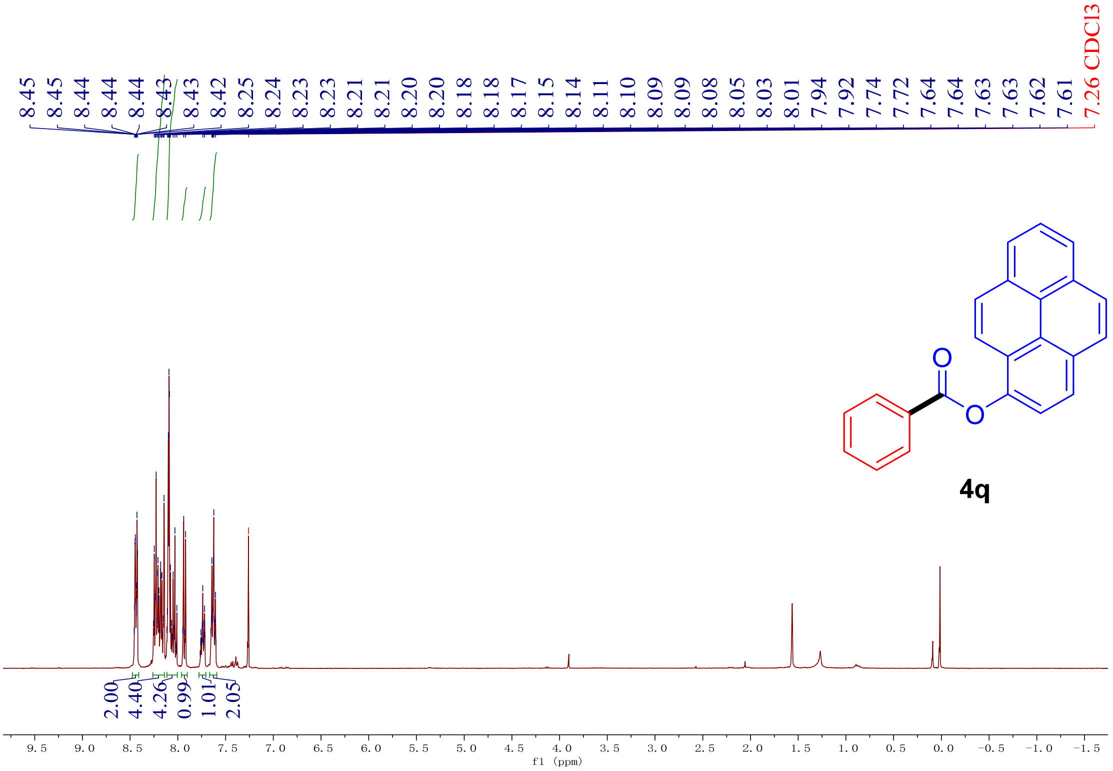

Figure S75. ^{13}C NMR spectrum of **4q** (100 MHz, $CDCl_3$)

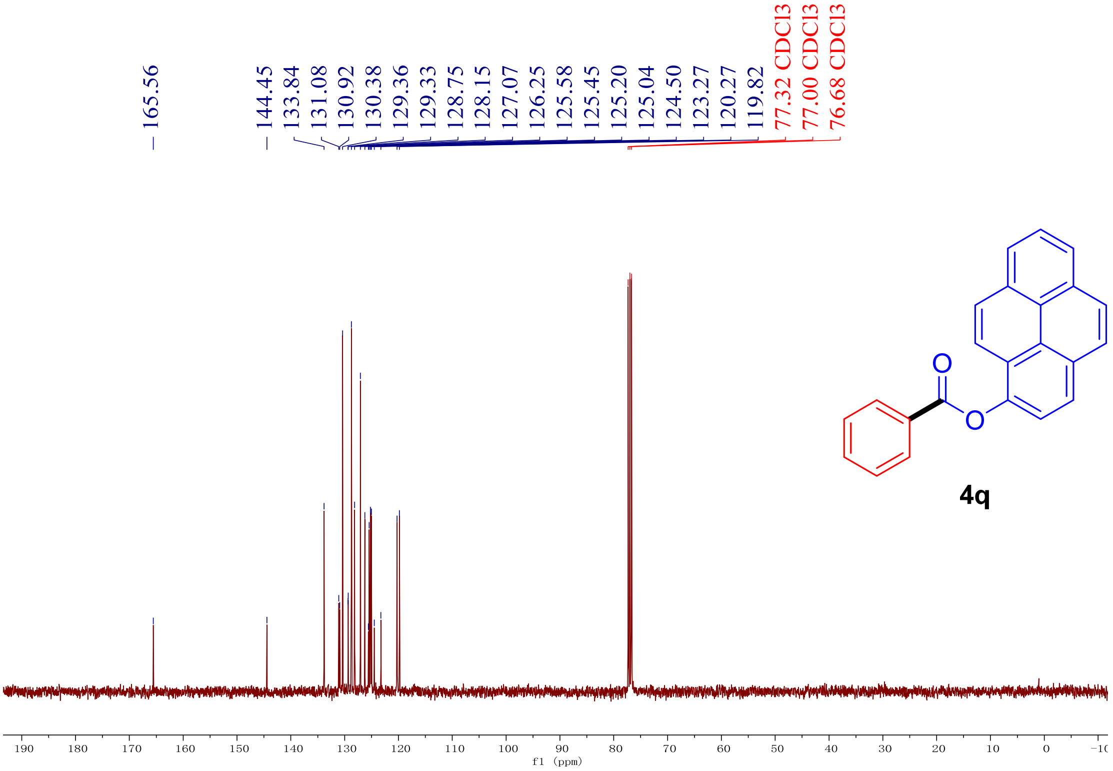

Figure S76. ^{1}H NMR spectrum of **4s** (400 MHz, $CDCl_3$)

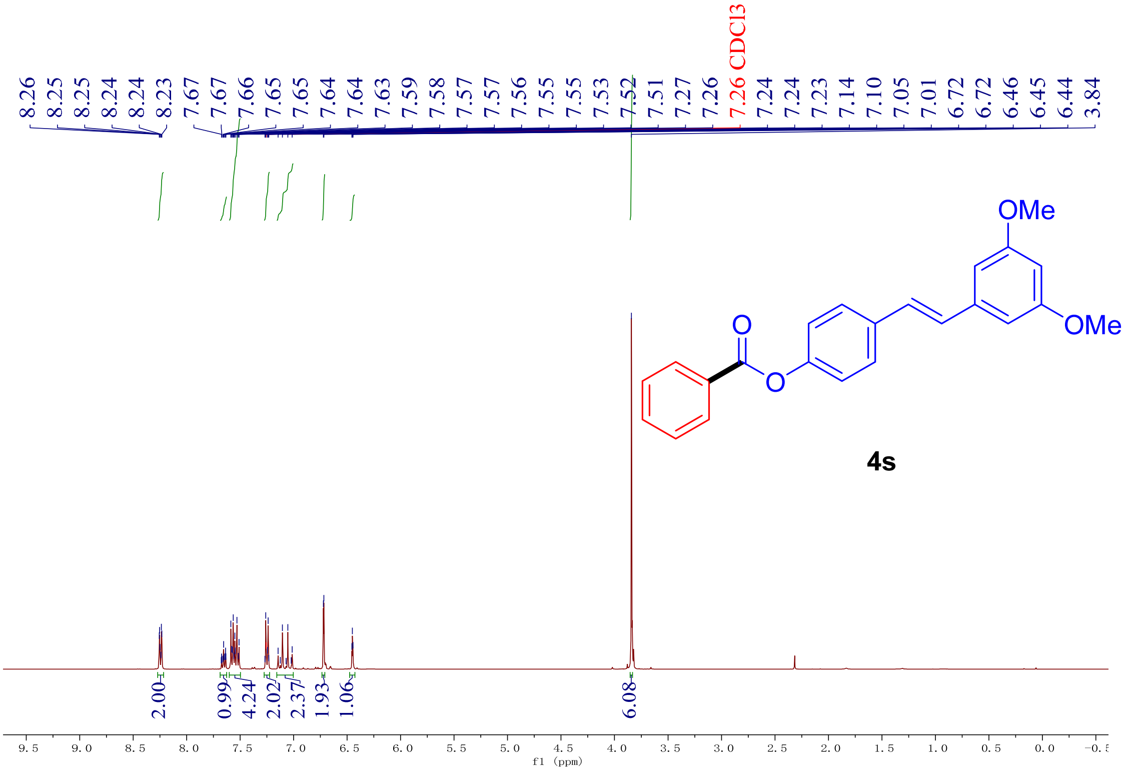

Figure S77. ^{13}C NMR spectrum of **4s** (100 MHz, $CDCl_3$)

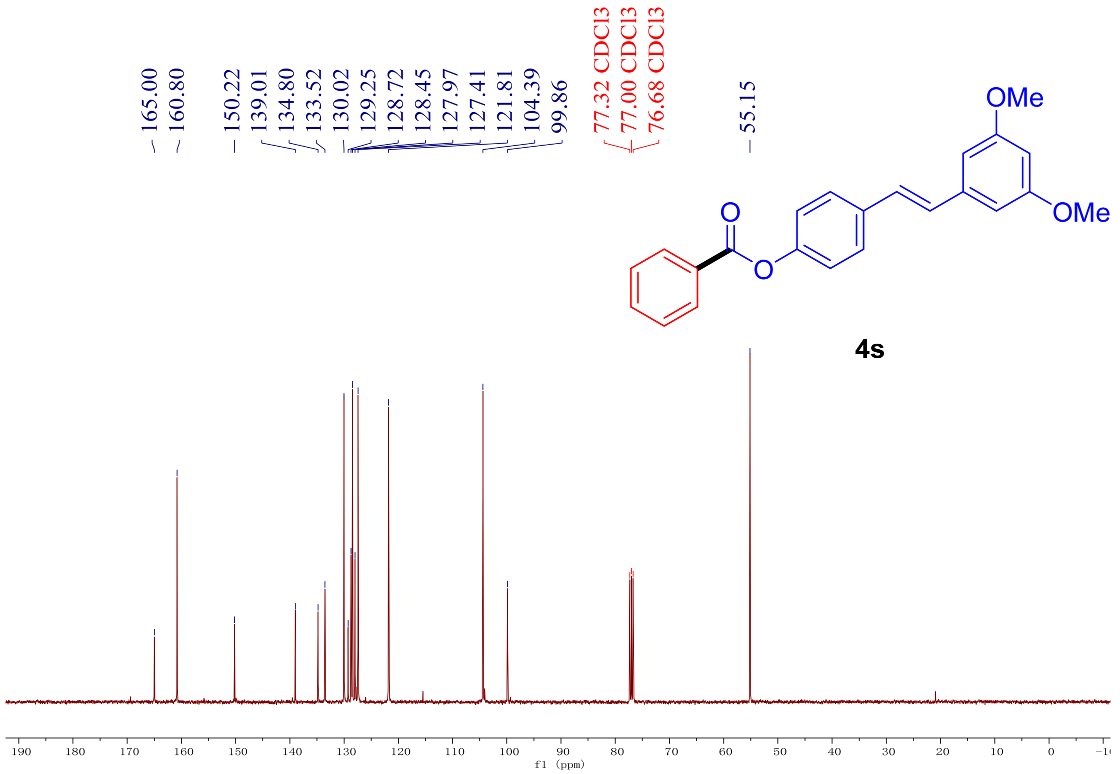

Supplement: Supplementary file 1 [file molecules-29-01991-s001.zip › molecules-2961060-supplementary.pdf]
